# Rational design of a topological polymeric solid electrolyte for high-performance all-solid-state alkali metal batteries

**DOI:** 10.1038/s41467-022-31792-5

**Published:** 2022-07-19

**Authors:** Yun Su, Xiaohui Rong, Ang Gao, Yuan Liu, Jianwei Li, Minglei Mao, Xingguo Qi, Guoliang Chai, Qinghua Zhang, Liumin Suo, Lin Gu, Hong Li, Xuejie Huang, Liquan Chen, Binyuan Liu, Yong-Sheng Hu

**Affiliations:** 1 Hebei Key Laboratory of Functional Polymer, School of Chemical Engineering and Technology, Hebei University of Technology, Tianjin, 300130 China; 2 Beijing National Laboratory for Condensed Matter Physics, Institute of Physics, Chinese Academy of Sciences, Beijing, 100190 China; 3 Yangtze River Delta Physics Research Center Co. Ltd, Liyang, 213300 China; 4 Center of Materials Science and Optoelectronics Engineering, University of Chinese Academy of Sciences, Beijing, 100190 China; 5 Huairou Division, Institute of Physics, Chinese Academy of Sciences, Beijing, 101400 China; 6 State Key Laboratory of Structural Chemistry, Fujian Institute of Research on the Structure of Matter, Chinese Academy of Sciences, Fuzhou, 350002 China; 7 Key Laboratory for Green Processing of Chemical Engineering of Xinjiang Bingtuan, School of Chemistry and Chemical Engineering, Shihezi University, Shihezi, 832003 China

**Keywords:** Batteries, Energy storage, Synthesis and processing, Energy, Materials for energy and catalysis

## Abstract

Poly(ethylene oxide)-based solid-state electrolytes are widely considered promising candidates for the next generation of lithium and sodium metal batteries. However, several challenges, including low oxidation resistance and low cation transference number, hinder poly(ethylene oxide)-based electrolytes for broad applications. To circumvent these issues, here, we propose the design, synthesis and application of a fluoropolymer, i.e., poly(2,2,2-trifluoroethyl methacrylate). This polymer, when introduced into a poly(ethylene oxide)-based solid electrolyte, improves the electrochemical window stability and transference number. Via multiple physicochemical and theoretical characterizations, we identify the presence of tailored supramolecular bonds and peculiar morphological structures as the main factors responsible for the improved electrochemical performances. The polymeric solid electrolyte is also investigated in full lithium and sodium metal lab-scale cells. Interestingly, when tested in a single-layer pouch cell configuration in combination with a Li metal negative electrode and a LiMn_0.6_Fe_0.4_PO_4_-based positive electrode, the polymeric solid-state electrolyte enables 200 cycles at 42 mA·g^−1^ and 70 °C with a stable discharge capacity of approximately 2.5 mAh when an external pressure of 0.28 MPa is applied.

## Abstract (web summary)

Solid-state polymer electrolytes are crucial for developing future rechargeable batteries, but they are still limited in performance. Here, the authors designed a topological polymeric solid electrolyte, enabling an all-solid-state high-voltage lithium metal pouch cell to cycle 200 times efficiently.

## Introduction

Among all secondary battery technologies, lithium-ion batteries (LIBs, abbreviation lists are in Supplementary Note 1) have experienced fierce competition but have eventually come to stand out by virtue of the best comprehensive performance. In recent years, solid-state batteries (SSBs) that exhibit relatively high energy densities and are safer than mainstream LIBs have been identified as the most forward-looking solution to meet the demanding requirements for next-generation batteries^1–3^. Poly(ethylene oxide)-based all-solid-state polymer electrolytes (PEO-ASPEs) have been widely studied for decades due to the nature of PEO (e.g., low glass transition temperature and good capability for dissolving lithium salts) and have been successfully equipped in electric vehicles of Bolloré group to afford efficient and safe power sources, which have been utilized in car-sharing services in several countries worldwide^4,5^. However, the progress of PEO-ASPE development is still hindered by their inherent limitations, among which poor high-voltage stability and low transference number are the main obstacles that hinder the broad applications of PEO-ASPEs^6–8^. Therefore, numerous target-oriented strategies have been proposed to overcome these disadvantages, including blending inorganic fillers^5,9,10^, blending organic materials^11,12^, adding topological polymers^13–15^, modifying the PEO matrix^16^, engineering a specific framework^17–19^, designing new salts^20,21^, and constructing stable interphases^22–24^. However, there are non-negligible limitations to such techniques upon utilization in practical circumstances, and a simple preparation strategy that can improve the comprehensive performance of PEO-ASPEs is urgently desired.

The following design principles can be considered to address these challenges. First, we chose to add a topological homopolymer to PEO-ASPEs based on considering the wide selection of functional monomers and preserving the existing advantages of PEO-ASPEs. Moreover, supramolecular self-assembly triggered by specific functional groups could introduce additional benefits, which have often been neglected^25^. 2,2,2-Trifluoroethyl methacrylate (TFEMA), which shows high stability (high thermal stability, chemical resistance, low highest occupied molecular orbital (HOMO) level) and abundant Li^+^ coordination groups (C=O, C-O, and C-F), could be a potentially suitable monomer to control the macromolecular structure and multifaceted tuning of material properties^26^. For topological structures, fluoropolymers with multi-arm and three-dimensional spherical structures are preferred and exhibit peculiar physical properties and electrochemical behaviors unattainable by simple linear homopolymers^27^. Cyclodextrin (*α*-type, *β*-type, and *γ*-type) reported by previous works has been demonstrated to be a suitable base material for post-functionalization or self-assembly, which could promote Li^+^ migration or realize other potential functions^28–33^. Here, the food additive *β*-cyclodextrin (*β*-CD) with 21 hydroxyl groups is selected as the “core” to obtain 21 functionalized arms, which is helpful to realize its high molecular size and high functionality. A more detailed introduction can be found in Supplementary Fig. 1 and Supplementary Note 2.

Here, we proposed a top-down design concept in which well-defined 21-arm fluoropolymers (21-*β*-CD-*g*-PTFEMA) with a postmodified *β*-CD core and 21 poly(2,2,2-trifluoroethyl methacrylate) (PTFEMA) arms were designed and synthesized through atom transfer radical polymerization (ATRP). The orthogonal test method determined the optimal composition of fluorine-rich macromolecule-containing all-solid-state polymer electrolyte (FMC-ASPE). Our research shed light on the formation and effects of supramolecular self-assembly between 21-*β*-CD-*g*-PTFEMA and PEO, which significantly improved the high-voltage stability and transference number (*t*_Li_^+^ = 0.88) to suppress the side reaction at the cathode side and dendrite growth at the Li metal side. Furthermore, the improved physical and electrochemical properties of the as-prepared FMC-ASPE also included high ionic conductivity, high toughness (2.7 times higher than PEO-ASPE), and high thermal stability. The synergistically improved comprehensive properties of FMC-ASPE promised significantly enhanced performances of all-solid-state lithium metal batteries (AS-LMBs). Based on these improvements, a high-voltage pouch cell (LiMn_0.6_Fe_0.4_PO_4_ (LMFP)|FMC-ASPE-Li|Li) shows a long cycling life (more than 200 cycles), and high safety was successfully assembled. Similar improvements were also realized in all-solid-state sodium metal batteries (AS-SMBs), demonstrating the flexibility of 21-*β*-CD-*g*-PTFEMA as a practical component for forward-looking PEO-based ASPEs.

## Results and discussion

**Fig. 1:**
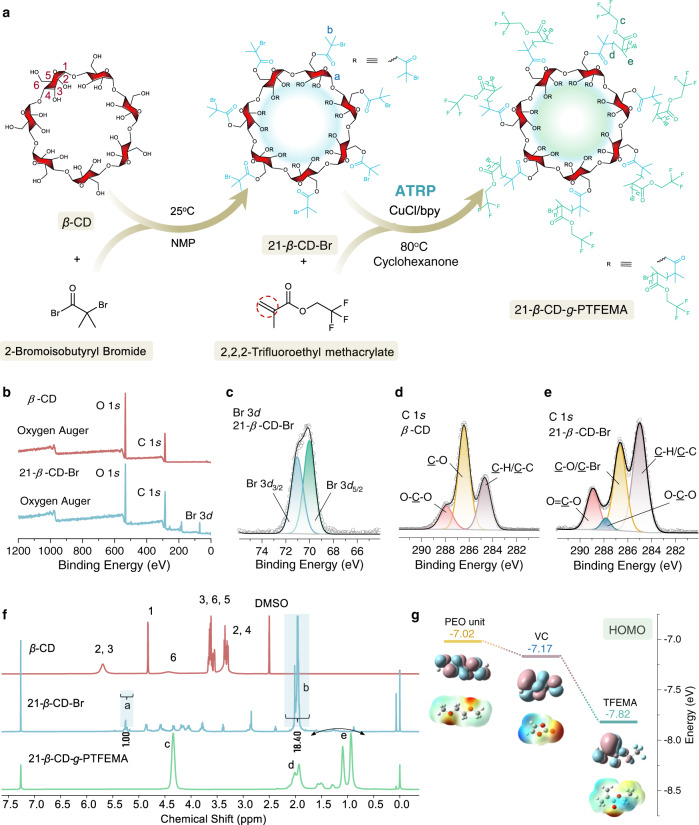
Synthetic route for 21-arm fluoropolymers (21-*β*-CD-*g*-PTFEMA) and corresponding physicochemical and theoretical characterizations. **a** Detailed synthetic scheme of 21-*β*-CD-*g*-PTFEMA. **b**–**e** X-ray photoelectron spectroscopy (XPS) results of *β*-CD and heptakis [2,3,6-tri-*O*-(2-bromo-2-methylpropionyl]-*β*-cyclodextrin (21-*β*-CD-Br): **b** full spectra of *β*-CD and 21-*β*-CD-Br, **c** Br 3*d* core level spectrum of 21-*β*-CD-Br, **d** C 1 *s* core level spectrum of *β*-CD, and **e** C 1 *s* core level spectrum of 21-*β*-CD-Br. **f** ^1^H nuclear magnetic resonance (^1^H NMR) spectra of *β*-CD, 21-*β*-CD-Br, and 21-*β*-CD-*g*-PTFEMA. **g** Density functional theory (DFT) analysis of the highest occupied molecular orbital (HOMO) and probability of electron cloud density distributions for different polymer units.

To prepare fluoropolymers using *β*-CD as a core via ATRP, it is crucial to introduce alkyl halides into *β*-CD, and the corresponding detailed synthetic scheme is shown in Fig. 1a. In this work, a tailor-made multifunctional macroinitiator, heptakis [2,3,6-tri-*O*-(2-bromo-2-methylpropionyl]-*β*-cyclodextrin (21-*β*-CD-Br), was synthesized via a one-step method between *β*-CD and 2-bromoisobutyryl bromide with anhydrous 1-methyl-2-pyrrolidone as the optimized solvent. This route demonstrated the highest efficiency, yield, and purity among the three synthesis routes reported thus far (Supplementary Fig. 2). All hydroxyl groups of *β*-CD were substituted with bromine groups, which were subsequently utilized as ATRP initiating sites for polymerizing the functional fluorene-containing methacrylate-type monomers. The complete esterification of the 21 hydroxyl groups was confirmed based on X-ray photoelectron spectroscopy (XPS, Fig. 1b–e and Supplementary Fig. 3), ^1^H nuclear magnetic resonance (NMR) (^1^H NMR, Fig. 1f), ^13^C NMR (Supplementary Fig. 4), Fourier transform infrared spectroscopy (FTIR, Supplementary Fig. 5), elemental analyses (Supplementary Table 1), and scanning electron microscopy with energy-dispersive X-ray spectrometry (SEM-EDS, Supplementary Fig. 6). More detailed results and discussions can also be found in Supplementary Note 3.

### Polymerization of fluoropolymers *via* ATRP

Tailor-made 21-*β*-CD-Br with multiple active sites was synthesized by an esterification reaction and acted as a highly efficient multifunctional macroinitiator for initiating the ATRP of monomers with double bonds. The fluorine-containing acrylate monomer TFEMA reduced the HOMO energy of the monomer and promoted the dissolution of lithium (sodium) salts, which depended on fluorine atoms and acrylate groups, respectively. Density functional theory (DFT) calculations were performed to gain deeper insight into the oxidation stability of the fluorinated monomer. As shown in Fig. 1g, compared to the widely studied polyether and polycarbonate ASPEs (traditional PEO and oxidation-resistant poly(vinylene carbonate) (PVC)), TFEMA has the lowest HOMO energy level (−7.82 eV), indicating a high antioxidation ability that is attributed to the strong electron-withdrawing effect of the fluorine atom. Based on this, a series of 21-*β*-CD-*g*-PTFEMA with high molecular weights were synthesized by ATRP using 21-*β*-CD-Br as a multifunctional macroinitiator and CuCl/bpy as the cocatalyst. The 21-*β*-CD-*g*-PTFEMA structure was unambiguously confirmed based on ^1^H NMR (Fig. 1f), ^19^F NMR (Supplementary Fig. 7), FTIR (Supplementary Fig. 8), XPS (Supplementary Fig. 9), SEM-EDS (Supplementary Fig. 10), and gel permeation chromatography (GPC) (Supplementary Fig. 11). More detailed results and discussions can also be found in Supplementary note 4.

### Preparation of FMC-ASPEs and design of the orthogonal experiment

The fluorine-rich macromolecule-containing all-solid-state polymer electrolyte, which includes 21-*β*-CD-*g*-PTFEMA, PEO, and lithium salt (sodium salt) for AS-LMBs (AS-SMBs) (FMC-ASPE-Li and FMC-ASPE-Na, respectively), was prepared through a conventional solution casting technique. Since FMC-ASPE based on 21-*β*-CD-*g*-PTFEMA was introduced, the performance was influenced by several empirical variables, resulting in the need for further experiments to screen the optimum ratio of each component in FMC-ASPEs. Therefore, there was an urgent need to design an orthogonal assay instead of an exhaustive method to comprehensively investigate various factors and levels and identify the optimal conditions with high efficiency (Tables 1, 2, Supplementary Figs. 12–41, Supplementary Tables 2, 3, and Supplementary Note 5 for details). Comprehensively, the optimal combination for the preparation of FMC-ASPE-Li involved a 21-*β*-CD-*g*-PTFEMA molecular weight of 900 kDa, a PEO-to-21-*β*-CD-*g*-PTFEMA mass ratio of 2: 1, and a LiTFSI-to-polymer mass ratio of 20%. The optimal combination for the preparation of FMC-ASPE-Na involved a 21-*β*-CD-*g*-PTFEMA molecular weight of 900 kDa, a PEO-to-21-*β*-CD-*g*-PTFEMA mass ratio of 4:1, and a NaTFSI-to-polymer mass ratio of 15%. Consequently, the two optimal combinations presented above were selected for the subsequent experiments on AS-LMBs and AS-SMBs. The commonalities between the two types of FMC-ASPEs for different metal batteries were the anion of the salts and the molecular weight of 21-*β*-CD-*g*-PTFEMA, indicating that the salts containing the TFSI^−^ anion and the 900 kDa molecular weight 21-*β*-CD-*g*-PTFEMA were conducive to the improvement in capacity retentions in high-voltage metal batteries^34^.

**Table 1:** The orthogonal experimental design and analysis for lithium metal batteries.

Factors and levels of the orthogonal L_4_ (2^3^) test			
Levels	Factors		
	A (M_n_, _GPC (21-*β*-CD-*g*-PTFEMA)_, g·mol^−1^)	B (*m*_PEO_: *m*_21-*β*-CD-*g*-PTFEMA_)	C (*m*_LiTFSI_: *m*_PEO+21-*β*-CD-*g*-PTFEMA_, %)
1	800k	4: 1	20
2	900k	2: 1	30

Evaluation indices of the orthogonal L_4_ (2^3^) test			
Orthogonal indices	Factors		
	A	B	C
K_1_ (%) ^(a)^	64.5	67.6	70.2
K_2_ (%) ^(a)^	73.8	70.7	68.1
R^(b)^	0.093	0.031	0.021
Order of Importance	A > B > C		
Optimal Level	A2	B2	C1
Optianl Composition	m_LiTFSI_: m_PEO + 21-*β*-CD-*g*-PTFEMA_ = 20% m_PEO_: m_21-*β*-CD-*g*-PTFEMA_ = 2: 1 M_n, GPC (21-*β*-CD-*g*-PTFEMA)_ = 900 kg mol^−1^ M_PEO_ = 600 kg mol^−1^		

^a^$${{{{{{\rm{K}}}}}}}_{{{{{{\rm{i}}}}}}}^{{{{{{\rm{F}}}}}}}=\frac{1}{2}(\sum {{{{{\rm{value}}}}}}\,{{{{{\rm{of}}}}}}\,{{{{{\rm{evalution}}}}}}\,{{{{{\rm{indexes}}}}}}\,{{{{{\rm{at}}}}}}\,{{{{{\rm{the}}}}}}\,{{{{{\rm{same}}}}}}\,{{{{{\rm{level}}}}}}\,{{{{{\rm{for}}}}}}\,{{{{{\rm{each}}}}}}\,{{{{{\rm{factor}}}}}})$$.

^b^${{{{{{\rm{R}}}}}}}^{{{{{{\rm{F}}}}}}}={\max} \{{{{{{{\rm{K}}}}}}}_{{{{{{\rm{i}}}}}}}^{{{{{{\rm{F}}}}}}}\}-{\min} \{{{{{{{\rm{K}}}}}}}_{{{{{{\rm{i}}}}}}}^{{{{{{\rm{F}}}}}}}\}$, where F stands for the different factors (A, B, and C) and i stands for the different levels (1, 2).

**Table 2:** The orthogonal experimental design and analysis for sodium metal batteries.

Factors and levels of the orthogonal L_9_ (3^4^) test				
Levels	Factors			
	A (Salts)	B (m_salt_: m_PEO + 21-*β*-CD-*g*-PTFEMA_, %)	C (m_PEO_: m_21-*β*-CD-*g*-PTFEMA_)	D (M_n, GPC (21-*β*-CD-*g*-PTFEMA)_, g·mol^−1^)
1	NaPF_6_	10	2: 1	100k
2	NaClO_4_	15	3: 1	800k
3	NaTFSI	20	4: 1	900k

Evaluation indices of the orthogonal L_9_ (3^4^) test				
Orthogonal indices	Factors			
	A	B	C	D
K_1_ (%) ^(a)^	61.4	58.4	44.5	60.5
K_2_ (%) ^(a)^	62.4	86.2	79.0	62.6
K_3_ (%) ^(a)^	83.7	63.0	84.1	84.4
R ^(b)^	0.223	0.278	0.396	0.239
Order of Importance	C > B > D > A			
Optimal Level	A3	B2	C3	D3
Optianl Composition	m_NaTFSI_: m_PEO + 21-*β*-CD-*g*-PTFEMA_ = 15% m_PEO_: m_21-*β*-CD-*g*-PTFEMA_ = 4: 1 M_n, GPC (21-*β*-CD-*g*-PTFEMA)_ = 900 kg mol^−1^ M_PEO_ = 600 kg mol^−1^			

^a^$${{{{{{\rm{K}}}}}}}_{{{{{{\rm{i}}}}}}}^{{{{{{\rm{F}}}}}}}=\frac{1}{3}(\sum {{{{{\rm{value}}}}}}\,{{{{{\rm{of}}}}}}\,{{{{{\rm{evalution}}}}}}\,{{{{{\rm{indexes}}}}}}\,{{{{{\rm{at}}}}}}\,{{{{{\rm{the}}}}}}\,{{{{{\rm{same}}}}}}\,{{{{{\rm{level}}}}}}\,{{{{{\rm{for}}}}}}\,{{{{{\rm{each}}}}}}\,{{{{{\rm{factor}}}}}})$$.

^b^${{{{{{\rm{R}}}}}}}^{{{{{{\rm{F}}}}}}}={\max} \{{{{{{{\rm{K}}}}}}}_{{{{{{\rm{i}}}}}}}^{{{{{{\rm{F}}}}}}}\}-\,{{\min}}\{{{{{{{\rm{K}}}}}}}_{{{{{{\rm{i}}}}}}}^{{{{{{\rm{F}}}}}}}\}$, where F stands for the different factors (A, B, C, and D) and i stands for the different levels (1, 2, and 3).

### Electrochemical, thermal, and mechanical stability tests and analysis

**Fig. 2:**
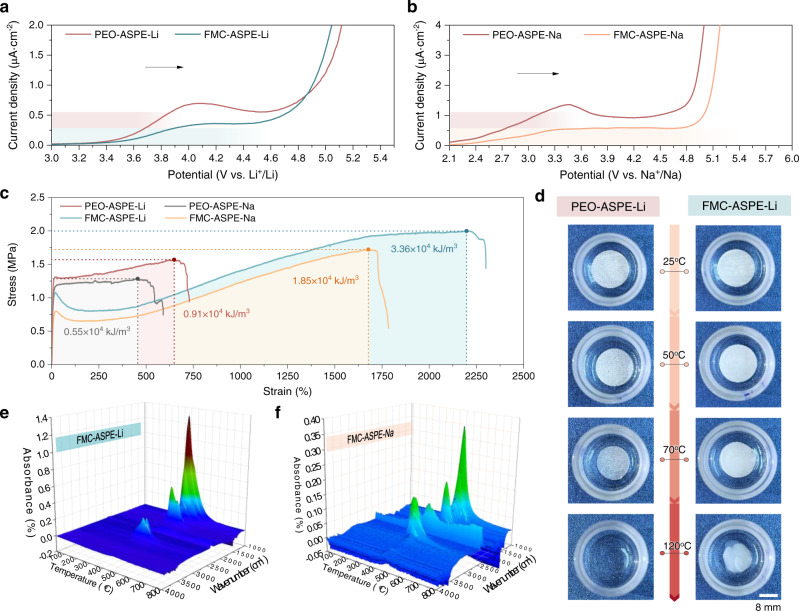
Electrochemical and thermal stability tests of the fluorine-rich macromolecule-containing and PEO-based polymer electrolytes. **a**, **b** Linear sweep voltammetry (LSV) test results for **a** FMC-ASPE-Li vs. PEO-ASPE-Li and **b** FMC-ASPE-Na vs. PEO-ASPE-Na. **c** The extension comparison of the four membranes. **d** Optical photographs of PEO-ASPE-Li and FMC-ASPE-Li heated to different temperatures. **e**, **f** Thermogravimetry-Fourier transform infrared spectroscopy (TG-FTIR) results for **e** FMC-ASPE-Li and **f** FMC-ASPE-Na.

The wide electrochemical stability window of ASPEs is essential for pursuing the high energy-density performances of AS-LMBs and AS-SMBs with high-voltage cathodes. Linear sweep voltammetry (LSV) tests were performed to monitor the electrochemical stabilities of the PEO-ASPEs and the FMC-ASPEs for AS-LMBs and AS-SMBs, respectively. The oxidation potential of PEO-ASPEs has been verified to be ~3.8 V *vs*. Li^+^/Li (or ~3.5 V vs. Na^+^/Na)^5,35,36^, according to which the corresponding current was chosen as the reference value of the oxidation potential for the FMC-ASPEs. The oxidation potentials of the FMC-ASPEs increased to ~4.7 V in the AS-LMBs and 5.0 V in the AS-SMBs, according to the LSV results shown in Fig. 2a, b; this increase indicated the suitability of the FMC-ASPEs for the high-voltage cathodes and was in good agreement with the HOMO analysis in Fig. 1g. The different oxidation stabilities of the FMC-ASPE-Li and FMC-ASPE-Na were mainly related to the different membrane compositions (salt concentration and the mass ratio between PEO and 21-*β*-CD-*g*-PTFEMA), which are fully discussed in the orthogonal experiment section. Furthermore, the DFT calculation results, which are shown in Supplementary Fig. 42 and Supplementary Table 4, demonstrate the formation of hydrogen bonds (bond energy ~−0.054 eV, bond length ~2.16 Å) between the C-F of the 21-*β*-CD-*g*-PTFEMA side chain and the O-H end of the PEO; such bonds could protect the PEO from undesired oxidation or reduction^37^. Altogether, the FMC-ASPEs with a wide electrochemical stability window matched the high-voltage cathodes, which was crucial in ensuring the enhancement of the energy-density performance of the AS-LMBs and AS-SMBs.

A freestanding ASPE membrane with high flexibility and mechanical strength is highly desirable for advanced compatibility with the electrodes and for providing safety. Figure 2c and Supplementary Table 5 summarize the mechanical performances of the PEO-ASPEs and the FMC-ASPEs for AS-LMBs and AS-SMBs, respectively. A tensile strength (*s*) of 1.6 MPa and elongation at break (*ε*_b_) of 649% were obtained for PEO-ASPE-Li. After adding 21-*β*-CD-*g*-PTFEMA, *s* and *ε*_b_ were enhanced to 2.0 MPa and 2198%, respectively. Based on this enhancement, the ASPE toughness was calculated, representing the strength and ductility of the fluoropolymers. Due to the high *s* and *ε*_b_ values achieved, the toughness of FMC-ASPE-Li reached 3.36 × 10^4^ kJ m^−4^, which was 3.7 times that of PEO-ASPE-Li. Likewise, the performance improvement in FMC-ASPE-Na was similar to that of FMC-ASPE-Li. High strength favors the suppression of Li dendrite growth, and high strain is a requirement for large-scale fabrication. The mechanism behind these prominent changes might be attributed to the inclusion of stiff groups in the fluoropolymers, where such stiff groups were assembled into a robust mechanical phase. In addition, supramolecular self-assembly between PEO and 21-*β*-CD-*g*-PTFEMA through noncovalent self-association produced desired FMC-ASPE toughness. This conclusion was supported by the DFT calculation results in Supplementary Fig. 43 and Supplementary Table 4, showing a substantial number of hydrogen bonds (bond energy ~−0.017 eV, bond length ~2.36 Å) between the C-F of 21-*β*-CD-*g*-PTFEMA and the C-H of PEO. Therefore, FMC-ASPE membranes exhibit good safety and commercial potential concerning their mechanical properties.

In addition to the mechanical properties, thermal stability is also notable for AS-LMB and AS-SMB safety. As shown in Fig. 2d, a thermal shrinkage comparison between the PEO-ASPE-Li and FMC-ASPE-Li membranes was carried out after heat treatment at different temperatures for 120 min. These two kinds of ASPE-Li membranes remained in their original shapes at 25 and 50 °C. Unfortunately, once the temperature increased above 70 °C, the PEO-ASPE-Li membrane incurred sudden and severe dimensional shrinkage and melted completely at 120 °C. In contrast, few consistent dimensional alterations occurred in the FMC-ASPE-Li membrane even when the temperature reached 120 °C. Furthermore, similar phenomena occurred for the FMC-ASPE-Na membrane (Supplementary Fig. 44). To further explore the thermostability of the FMC-ASPE membranes, thermogravimetry-Fourier transform infrared spectroscopy (TG-FTIR) was utilized (Fig. 2e, f and Supplementary Figs. 45,46). The results revealed that with the addition of 21-*β*-CD-*g*-PTFEMA, the FMC-ASPEs for either the AS-LMBs or AS-SMBs underwent complete decomposition starting at 400 °C, and the apparent mass loss was also demonstrated based on the TGA results in Supplementary Figs. 47, 48. The improved thermal stability also benefitted from the formation of hydrogen bonds, in addition to the high-temperature-stable nature of PTFEMA. Based on the results, the thermal stability of the FMC-ASPE membranes was verified; this stability could ensure the steady operation of AS-LMBs (AS-SMBs) under high-temperature conditions.

**Fig. 3:**
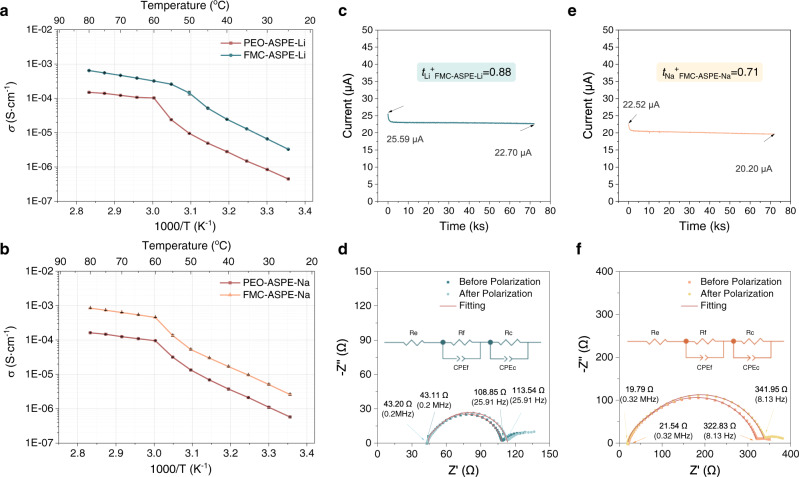
Ion conduction properties of the FMC-ASPE-Li/Na and PEO-ASPE-Li /Na membranes. **a**, **b** Ionic conductivity-temperature functions of **a** FMC-ASPE-Li vs. PEO-ASPE-Li and **b** FMC-ASPE-Na vs. PEO-ASPE-Na. **c**–**f** Cation transference number test results of FMC-ASPE-Li and FMC-ASPE-Na. Polarization (POL) result (**c**) and fitted electrochemical impedance spectra (EIS) before and after the POL (**d**) of FMC-ASPE-Li, POL result (**e**), and fitted EIS before and after POL (**f**) of FMC-ASPE-Na.

The temperature-dependent ionic conductivities of the PEO-ASPEs and the FMC-ASPEs for AS-LMBs and AS-SMBs, respectively, were recorded as a function of temperature through the alternating current impedance method. As shown in Fig. 3a, b, the addition of 21-*β*-CD-*g*-PTFEMA has a positive effect on the ionic conductivity over the whole temperature range from 25 to 80 °C (Supplementary Tables 6, 7). Moreover, the ionic conductivity reached 6.43 × 10^−4^ S cm^−1^ and 8.43 × 10^−4^ S cm^−1^ at 80 °C for FMC-ASPE-Li and FMC-ASPE-Na, respectively. Indeed, the introduction of amorphous 21-*β*-CD-*g*-PTFEMA minimized the crystalline region. Furthermore, except for the C-O-C group of PEO, 21-*β*-CD-*g*-PTFEMA provided several additional ion conduction pathways through the C=O, C-O-C, and C-F polar groups to enhance the ionic conductivity of the FMC-ASPEs^38^. According to the DFT calculation results in Supplementary Figs. 49–52 and Supplementary Table 8, the absorption between the abovementioned polar groups and Li^+^ was confirmed, indicating an abundant and diverse Li^+^ coordination environment synergistically enhanced Li^+^ transfer. Notably, although the adsorption energy of the C=O···Li^+^ coordination (−0.683 eV) of 21-*β*-CD-*g*-PTFEMA was higher than that of the C-O···Li^+^ coordination (−0.457 eV) of PEO, the coordination number of the former (~3) was widely demonstrated to be lower than that of the latter (~5), indicating that the C=O group provided better Li^+^ conductivity than the C-O-C group of PEO^39,40^.

**Fig. 4:**
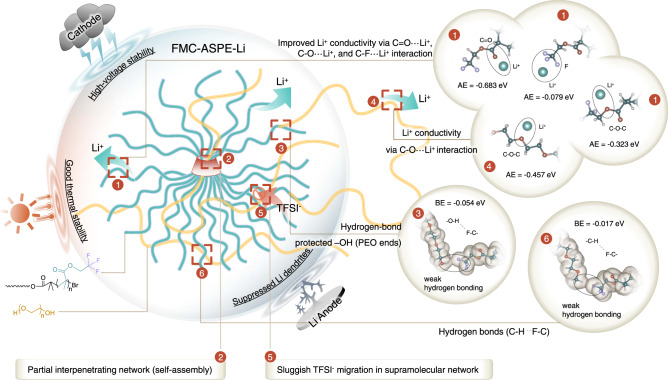
Schematic of the interactions in the FMC-ASPE-Li membrane. The macromolecule–macromolecule and macromolecule–ion interactions are highlighted with red squares and serial numbers (Nos. 1–6) to supplement the DFT calculation results. Four polar groups are demonstrated to synergistically improve Li^+^ movement (higher Li^+^ conductivity and higher Li^+^ transfer number). In addition, two kinds of weak hydrogen bonds in FMC-ASPE-Li are beneficial for lowering crystallinity, improving mechanical strength, protecting the end hydroxyl of PEO from oxidation/reduction, hindering TFSI^−^ anion motivation, and improving the thermal stability of the polymer electrolyte. The adsorption energy (AE) between the polar groups and Li^+^ and the bond energy (BE) of the weak hydrogen bonds are annotated in the figure.

*t*_Li_^+^ (*t*_Na_^+^) is a crucial parameter for ASPEs because a low *t*_Li_^+^(*t*_Na_^+^) results in an increase in the electrode polarization and number of overgrown Li dendrites. The *t*_Li_^+^(*t*_Na_^+^) of ASPEs was measured through chronoamperometry combined with electrochemical impedance spectroscopy. Figure 3c exhibits the time-dependent response of the direct-current polarization of FMC-ASPE-Li, and Fig. 3d shows the impedance spectra before and after chronoamperometry. The impedance spectrum fitting details are summarized in Supplementary Tables 9, 10, and the calculation details are included in Supplementary Table 11. Likewise, the FMC-ASPE-Na results are depicted schematically in Fig. 3e, f, and the detailed parameters are summarized in Supplementary Tables 12–14. The calculated *t*_Li_^+^ of FMC-ASPE-Li was 0.88, and the *t*_Na_^+^ of FMC-ASPE-Na was ~0.71. Figure 4 summarizes the interactions in the FMC-ASPE-Li membrane to provide an intuitive understanding of the supermolecular interactions and Li^+^ migration mechanism in FMC-ASPE.

**Fig. 5:**
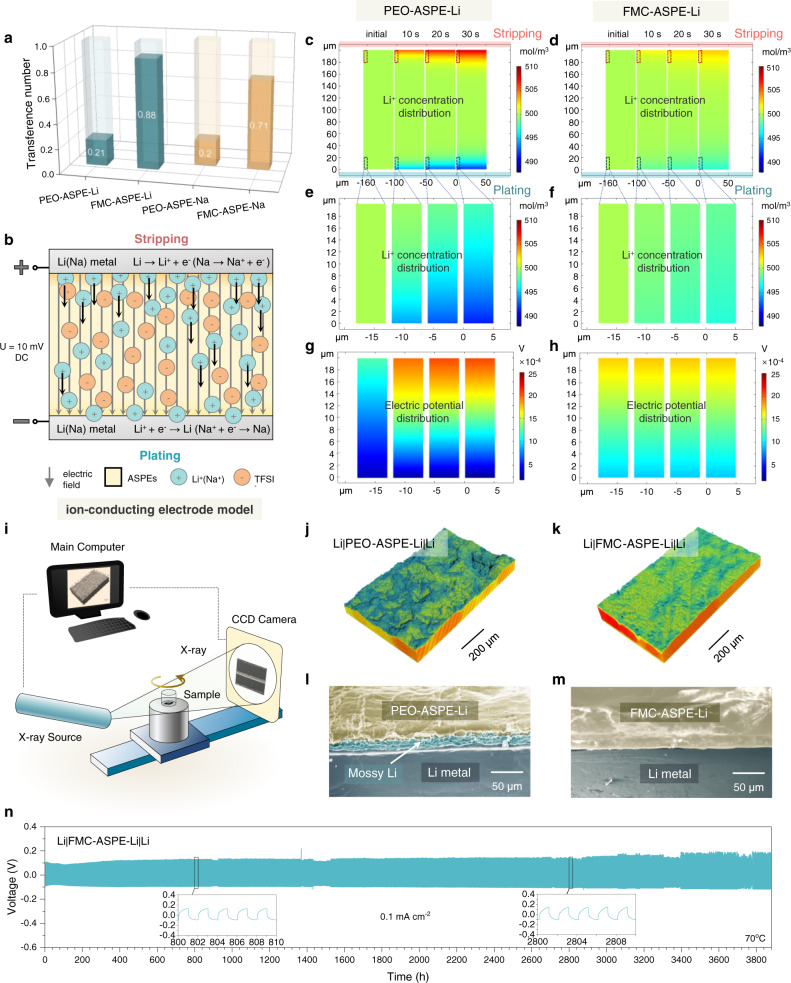
Investigation of the lithium- and sodium- ion transference number via electrochemical measurements and simulations. **a** Comparison of the transference number before and after the addition of 21-*β*-CD-*g*-PTFEMA. **b** The physical model used for the finite-element method simulations (FEMSs). **c**–**h** The Li^+^ concentration distribution obtained from FEMS results of the **c** whole region of PEO-ASPE-Li membrane, **d** whole region of FMC-ASPE-Li membrane, **e** near-region of the Li-plating side in the PEO-ASPE-Li membrane, and **f** near-region of the Li-plating side in the FMC-ASPE-Li membrane; the electric potential distribution obtained from FEMS results near the region of the Li-plating side in the **g** PEO-ASPE-Li membrane and **h** FMC-ASPE-Li membrane. **i** Schematic diagram of the working principle of X-ray tomography. **j**, **k** 3D reconstructions of the **j** PEO-ASPE-Li|Li and **k** FMC-ASPE-Li|Li interfaces after ten plating/stripping cycles, showing significantly different roughnesses. **l**, **m** Cross-sectional SEM images of **l** Li|PEO-ASPE-Li|Li and **m** Li|FMC-ASPE-Li|Li cells after cycling. **n** Cycling performance of the Li|FMC-ASPE-Li|Li symmetrical cell at a current density of 0.1 mA cm^−2^ at 70 °C.

The determined *t*_Li_^+^ of FMC-ASPE-Li (0.88) was ~4.4 times larger than that for PEO-ASPE-Li (*t*_Li_^+^ = 0.21, see Fig. 5a, Supplementary Fig. 53, and Supplementary Table 11). This improvement trend was also pronounced for FMC-ASPE-Na. The calculated *t*_Na_^+^ value of FMC-ASPE-Na (0.71) was 3.5 times larger than that for PEO-ASPE-Na (*t*_Na_^+^ = 0.2, see Fig. 5a, Supplementary Fig. 54, and Supplementary Table 14), and the values were comparable to those of the “single-ion” conductors^41^. As a bottleneck limiting the high-rate performance, the transference number of cations is related to the migration capability of both cations and anions. In this work, the high *t*_Li_^+^ and *t*_Na_^+^ values of FMC-ASPEs are related to macromolecule–ion and macromolecule–macromolecule interactions, which improve cation migration and limit anion migration. On the one hand, a rich diversity of Li^+^ (Na^+^) coordination (C=O···Li^+^ (Na^+^), C-O···Li^+^ (Na^+^), and C-F···Li^+^ (Na^+^)) is helpful to increase the Li^+^ (Na^+^) conductivity, as discussed above. On the other hand, the hydrogen bonds (O-H···F-C and C-H···F-C) and self-assembly phenomenon in FMC-ASPE contribute to the formation of a complex cross-linked network, which could significantly hinder the migration of TFSI^−^ anions, as shown in Fig. 4. Furthermore, the in situ formed hydrogen bonds could also provide additional migration routes for Li migration.

To further quantitatively evaluate the interfacial stability regulated by the high *t*_Li_^+^ (*t*_Na_^+^) and explore the concentration gradients induced by ion diffusion under an applied electric field, the concentration distribution of Li^+^ (Na^+^) and electric potential distribution of the system were simulated via finite-element method simulations (FEMSs) using COMSOL Multiphysics. The “ion-conducting electrode” model was chosen, as illustrated in Fig. 5b; this model approximates the symmetrical Li|ASPE-Li|Li (Na|ASPE-Na|Na) cell more closely than the “ion-blocking electrode” model^42^. The detailed simulation methods and parameters are presented in Supplementary Tables 15–18. As shown in Fig. 5c–f and Supplementary Fig. 55, FMC-ASPE-Li presented smaller concentration gradients of Li^+^ during plating and stripping than PEO-ASPE-Li. Clearly, the addition of 21-*β*-CD-*g*-PTFEMA to PEO-ASPE-Li improved the homogenous environment from FMC-ASPE-Li to the surface of Li metal; this improvement effectively changed the large electric field distribution, tuned the lithium deposition, and prevented lithium dendrite growth (Fig. 5g, h). The same trend was common in the simulation of FMC-ASPE-Na (PEO-ASPE-Na) and observed in Supplementary Fig. 56. These results were consistent with Chazalviel’s model^43^ and the “Sand’s time” equation^44^ (Supplementary Note 6).

In addition to the simulation results, to compare the morphology differences between the FMC-ASPE-Li|Li and PEO-ASPE-Li|Li interfaces, we carried out non-destructive X-ray tomography tests (Fig. 5i–k), and scanning electron microscopy (SEM, Fig. 5l, m) on the Li|FMC-ASPE-Li|Li and Li|PEO-ASPE-Li|Li symmetrical cells stopped after ten plating/stripping cycles (0.1 mA cm^−2^, 0.1 mAh cm^−2^). As shown in Fig. 5j, severe Li dendrite growth (mossy Li) was found at the PEO-ASPE-Li|Li interface, showing heterogeneous nucleation and undulating morphology For the FMC-ASPE-Li|Li interface, shown in Fig. 5k, almost no Li dendrites were observed, indicating uniform plating/stripping and mechanically suppressed growth. Similar results could also be found in the cross-sectional SEM images (Fig. 5l, m), showing a mossy Li layer thickness of ~20 μm at the PEO-ASPE-Li|Li interface and quiet flat FMC-ASPE-Li|Li interface. The significant morphology differences between the two interfaces further demonstrated the advanced Li^+^ transference capability and mechanical strength of the FMC-ASPE-Li membrane.

The long-term electrochemical cycling stability of FMC-ASPE-Li with high *t*_Li_^+^ was further assessed based on experimental validation of the results obtained from symmetrical cells. Figure 5n shows the cycling performance of Li|FMC-ASPE-Li|Li symmetrical cells at a current density of 0.1 mA cm^−2^ at 70 °C, exhibiting long cycling stability for more than 3800 h with a nearly constant polarization voltage of 0.1 V. There appeared to be no short circuit phenomenon, suggesting effective inhibition of dendritic lithium formation. As mentioned above, the high cation transference number of FMC-ASPE-Li was ascribed to the presence of specific domains with high-affinity supramolecular interactions.

### Battery testing of the polymeric solid-state electrolyte in various full lithium and sodium metal cell configurations

**Fig. 6:**
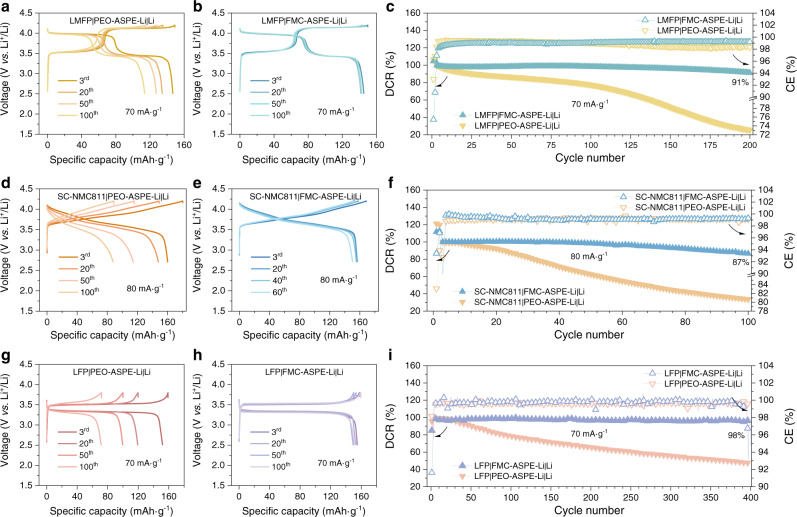
Li-ion storage performance of the all-solid-state lithium metal batteries with different cathodes and all-solid-state polymer electrolytes (CR2032 coin cell, 70 °C). **a**, **b** Comparison of the charge/discharge voltage profiles of **a** LMFP|PEO-ASPE-Li|Li and **b** LMFP|FMC-ASPE-Li|Li full cells. **c** Comparison of discharge capacity retention (DCR) and coulombic efficiency (CE) of LiMn_0.6_Fe_0.4_PO_4_ (LMFP)|PEO-ASPE-Li|Li and LMFP|FMC-ASPE-Li|Li full cells; **d**, **e** Comparison of the charge/discharge voltage profiles of **d** single-crystal LiNi_0.8_Mn_0.1_Co_0.1_O_2_ (SC-NMC811)|PEO-ASPE-Li|Li and **e** SC-NMC811|FMC-ASPE-Li|Li full cells. **f** Comparison of DCR and CE of SC-NMC811|PEO-ASPE-Li|Li and SC-NMC811|FMC-ASPE-Li|Li full cells. **g**, **h** Comparison of the charge/discharge voltage profiles of **g** LiFePO_4_ (LFP)|PEO-ASPE-Li|Li and **h** LFP|FMC-ASPE-Li|Li full cells. **i** Comparison of DCR and CE of LFP|PEO-ASPE-Li|Li and LFP|FMC-ASPE-Li|Li full cells. The mass of the positive electrode active material was used for calculating the specific capacity and specific current.

**Fig. 7:**
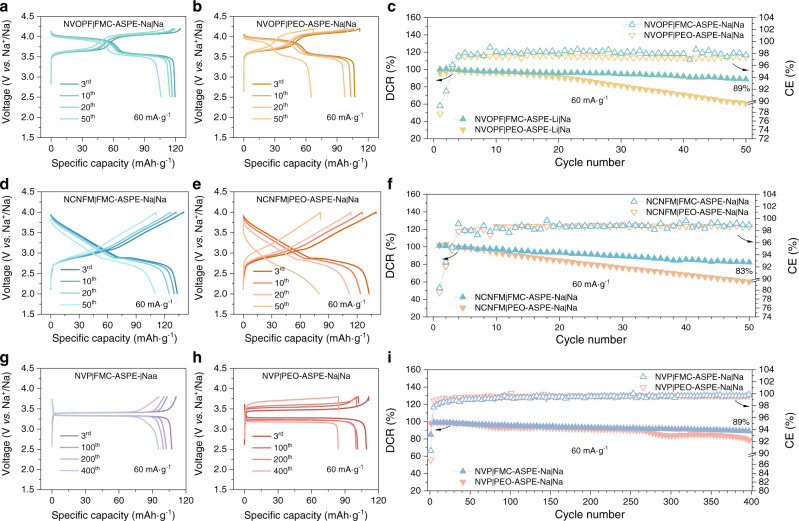
Na-ion storage performance of the all-solid-state sodium metal batteries with different cathodes and all-solid-state polymer electrolytes (CR2032 coin cell, 80 °C). **a**, **b** Comparison of the charge/discharge voltage profiles of **a** Na_3_(VOPO_4_)_2_F (NVOPF)|PEO-ASPE-Na|Na and **b** NVOPF|FMC-ASPE-Na|Na full cells. **c** Comparison of DCR and CE of NVOPF|PEO-ASPE-Na|Na and NVOPF|FMC-ASPE-Na|Na full cells; **d**, **e** Comparison of the charge/discharge voltage profiles of **d** NaCu_1/9_Ni_2/9_Fe_1/3_Mn_1/3_O_2_ (NCNFM)|PEO-ASPE-Na|Na and **e** NCNFM|FMC-ASPE-Na|Na full cells. **f** Comparison of DCR and CE of NCNFM|PEO-ASPE-Na|Na and NCNFM|FMC-ASPE-Na|Na full cells. **g**, **h** Comparison of the charge/discharge voltage profiles of **g** Na_3_V_2_(PO_4_)_3_ (NVP)|PEO-ASPE-Na|Na and **h** NVP|FMC-ASPE-Na|Na full cells. **i** Comparison of DCR and CE of NVP|PEO-ASPE-Na|Na and NVP|FMC-ASPE-Na|Na full cells. The mass of the positive electrode active material was used for calculating the specific capacity and specific current.

To further verify the electrochemical performance of the FMC-ASPEs, LMFP|FMC-ASPE-Li|Li, single-crystal LiNi_0.8_Co_0.1_Mn_0.1_O_2_ (SC-NMC811)|FMC-ASPE-Li|Li, LiFePO_4_ (LFP)|FMC-ASPE-Li|Li, Na_3_(VOPO_4_)_2_F (NVOPF)|FMC-ASPE-Na|Na, NaCu_1/9_Ni_2/9_Fe_1/3_Mn_1/3_O_2_ (NCNFM)|FMC-ASPE-Na|Na, and Na_3_V_2_(PO_4_)_3_ (NVP)|FMC-ASPE-Na|Na full coin cells were assembled and tested (Figs. 6,7).

High-voltage LMFP|FMC-ASPE-Li|Li cells (70 mA g^−1^, 2.5–4.2 V, 70 °C) with an average discharge voltage of ~3.7 V exhibited a discharge capacity retention of 91% after 200 cycles, as shown in Fig. 6a–c. In addition, the cycling stability of the high-voltage SC-NMC811 cathode was also improved, and SC-NMC811|FMC-ASPE-Li|Li cells showed 87% discharge capacity retention (80 mA g^−1^, 2.7–4.2 V, 70 °C) after 100 cycles (Fig. 6d–f). For the LFP|FMC-ASPE|Li full cells, steady cycling stability of 98% after 400 cycles (70 mA g^−1^, 2.5–3.8 V, 70 °C) was obtained, as shown in Fig. 6g–i, demonstrating the long-term stability of the Li metal anode during repeated stripping and plating, both chemically and mechanically. Similar improvements were also found for the AS-SMBs with FMC-ASPE-Na, as shown in Fig. 7. The discharge capacity retention of the high-voltage NVOPF|FMC-ASPE-Na|Na (60 mA g^−1^, 2.5–4.2 V, 80 °C), NCNFM|FMC-ASPE-Na|Na (60 mA g^−1^, 2.0–4.0 V, 80 °C) full cells were improved from 61 to 89% and 60 from to 83%, respectively, as shown in Fig. 7a–f. Furthermore, the NVP|FMC-ASPE-Na|Na full cells (Fig. 7g–i) showed cycling stability of 89% after 400 charge and discharge cycles (60 mA g^−1^, 2.5–3.8 V, 80 °C). The good electrochemical energy storage performance of coin cells with FMC-ASPEs motivated us to explore the behavior of single-layer lab-scale pouch cells (56.25 cm^2^) further.

**Fig. 8:**
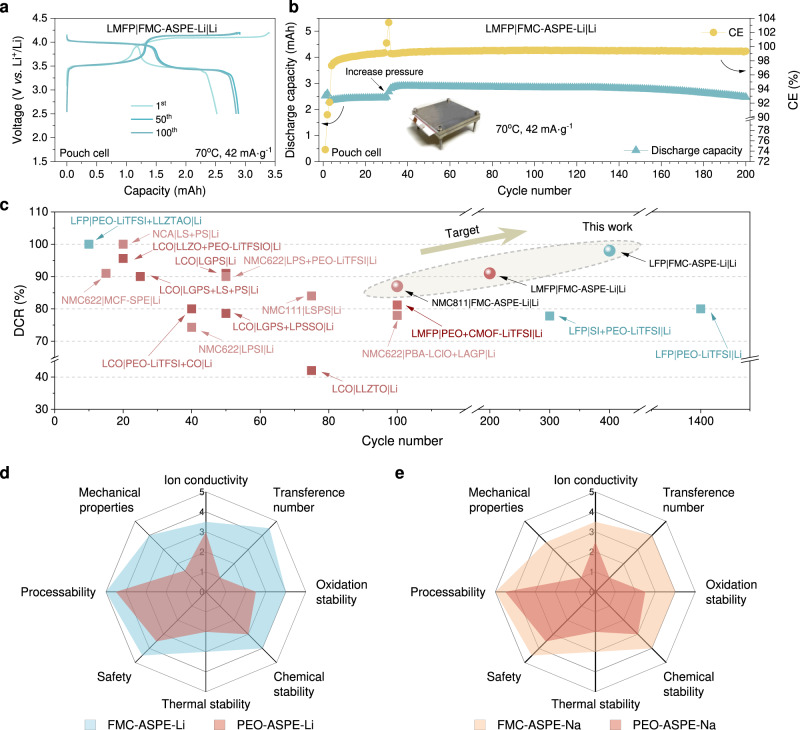
Cycling performance of lithium metal pouch cells with the LMFP cathode and FMC-ASPE-Li. **a**, **b** Charge/discharge voltage profiles **a** for different cycles and corresponding **b** Discharge capacity with the cycle number of the LMFP|FMC-ASPE-Li|Li pouch cell, with the photo of the test fixture inserted. The mass of the positive electrode active material was used for calculating the specific current. The initial pressure applied was 0.63 × 10^−3^ MPa, and the increased pressure after the screws was 0.28 MPa after 30 cycles. **c** Comparison of cycling performance of different all-solid-state lithium batteries (LiCoO_2_ (LCO), Li(Ni_0.6_Co_0.2_Mn_0.2_)O_2_ (NCM622), Li(Ni_0.3_Co_0.3_Mn_0.3_)O_2_ (NCM111), Li(Ni_0.8_Co_0.15_Al_0.05_)O_2_ (NCA), poly(ethylene oxide) (PEO), LiFePO_4_ (LFP), LiMn_0.6_Fe_0.4_PO_4_ or LiMn_0.85_Fe_0.15_PO_4_ (LMFP), poly(1,4-butylene adipate) (PBA), single-ion conductors (SI), lithium bis(trifluoromethanesulfonyl)imide (LiTFSI), LiClO_4_ (LClO), Li_10_SiP_2_S_12_ (LSPS), Li_10_GeP_2_S_12_ or Li_3.15_Ge_0.15_P_0.85_S_4_ (LGPS), Li_2_S (LS), P_2_S_5_ (PS), Li_3_P_1-x_Sb_x_S_4-2.5x_O_2.5x_ (LPSSO), Li_7_P_2_S_8_I (LPSI), Li_1.3_Al_0.3_Ti_1.7_(PO_4_)_3_ (LLZTO), Al-Li_6.75_La_3_Zr_1.75_Ta_0.25_O_12_ (LLZTAO), *β*-Li_3_PS_4_ (LPS), main-chain fluorinated solid polymer electrolytes (MCF-SPE), CMOF, D-UiO-66-NH_2_, CeO_2_ (CO). The corresponding references are listed in Supplementary Table 19). **d**, **e** Radar plots of the performance properties of different ASPEs.

To demonstrate the commercialization prospects of FMC-ASPEs, pouch cells with LMFP cathodes and lithium metal anodes were assembled and schematically illustrated in Supplementary Figs. 57–59. As shown in Fig. 8a, the LMFP|FMC-ASPE-Li|Li pouch cell (design capacity of 2.8 mAh) was cycled in the voltage range of 2.5–4.2 V (42 mA g^−1^, 70 °C). The pouch cell showed stable cycling performance over 200 cycles (Fig. 8b), exhibiting ~2.47 mAh at the 200th cycle and an average coulombic efficiency of ~99.4%. External pressure was applied to the pouch cell to improve the electrode/all-solid-state electrolyte contact and increase the internal stress to suppress lithium dendrite growth. Increasing the external pressure (0.63 × 10^−3^ MPa for the first 30 cycles and then 0.28 MPa) helped achieve the design capacity and long cycling life. The LMFP|FMC-ASPE-Li|Li pouch cell could still power a LED-based device after 200 cycles, as shown in Supplementary Fig. 57c, d. Better cycling was achieved by modifying the cathode/ASPE interface, for instance, by coating inorganic electrolytes (Li_1.5_Al_0.5_Ti_1.5_(PO_4_)_3_, Li_7_La_3_Zr_2_O_12_, etc.), as introduced in Supplementary Figs. 60–67 and Supplementary Note 7. To further assess the safety of the pouch cell, various tests under harsh conditions were conducted, including bending deformation, folding, nail penetration, and cutting. After such tests, the pouch cell still delivered a stable current to power a light-emitting diode with a bright green color, as represented in Supplementary Fig. 68. Moreover, no abnormalities, such as smoking or fire evolution, occurred during such abusive operations, and the open-circuit voltage did not show a significant drop after all the safety tests (4.16 vs. 3.98 V). Advanced cycling performance of high-voltage AS-LMBs using FMC-ASPE-Li could also be confirmed compared with other different reported results, as shown in Fig. 8c and Supplementary Table 19. The performance properties of FMC-ASPEs and PEO-ASPEs are summarized in radar plots of the comprehensive performance, as depicted in Fig. 8d, e. The all-in-one strategy also exhibits advantages over other common strategies, as compared and discussed in Supplementary Table 20 and Supplementary Note 8.

In summary, we have successfully engineered a well-defined multi-arm fluoropolymer, 21-*β*-CD-*g*-PTFEMA, based on *β*-CD through ATRP. In addition to the advanced natures of PTFEMA and PEO, the introduced supramolecular interactions (arising from C=O, C-O, C-F, O-H, and C-H bonds) and multi-arm topological structures were validated to be critical factors for improving the abovementioned properties beyond two mutually independent macromolecules. This work not only enriches the library of polyfunctional ASPEs for next-generation energy storage technology but also contributes to an ingenious design concept for constructing domain-specific and highly functional material systems.

## Methods

### Materials for the synthesis of 21-*β*-CD-*g*-PTFEMA

All reagents were purchased from commercial suppliers and used without further purification unless otherwise stated. All manipulations involving air- and/or water-sensitive compounds were carried out with the standard Schlenk and vacuum line techniques or glove box techniques under an argon atmosphere (H_2_O and O_2_ < 0.1 ppm). *β*-Cyclodextrin (*β*-CD; 99%, Tokyo Chemical Industry Co., Ltd. (TCI)) was recrystallized twice in deionized water, dried in vacuo at 80 °C for 24 h, and then stored in an amber wide mouth packer in a glove box before use. 2,2,2-Trifluoroethyl methacrylate (TFEMA; 99%, Acros (Acros Organics, Geel, Belgium)) was washed with a 2 wt% aqueous solution of sodium hydroxide and then with double-distilled water several times until neutralization to remove the inhibitor (4-methoxyphenol, MEHQ). The organic layer was collected and dried over anhydrous magnesium sulfate before vacuum distillation before use. Copper (I) chloride (CuCl; ≥99.995%, Sigma-Aldrich) was purified by stirring in glacial acetic acid at 80 °C for 12 h, filtrated, rinsed with cold ethanol and diethyl ether in an argon environment for 10 min successively, and dried in vacuum at 35 °C for 48 h. 2-Bromoisobutyryl bromide (BIBB; 98%, TCI), 2,2′-bipyridyl (bpy; 99%, TCI), aluminum oxide (200–300 mesh, Shanghai Aladdin Biochemical Technology Co., Ltd. (Aladdin)), and sodium bicarbonate (NaHCO_3_; ≥99.8%, Aladdin) were used as received by the suppliers. *n*-Hexane (97%, Aladdin) and 1-methyl-2-pirrolidone anhydrous (NMP; 99.5%, Alfa Aesar (Alfa Aesar, Wardhill, MA)) were used without further purification. Cyclohexanone (>99.5%, Adamas-beta) was initially decolored by active carbon for 2 h and then stirred with calcium hydride (CaH_2_; 95%, Aladdin) overnight before distillation under reduced pressure before use. Other solvents were purchased from Aladdin and refluxed over desiccants under argon before use: dichloromethane (CH_2_Cl_2_) from CaH_2_.

### Synthetic procedures of 21-*β*-CD-*g*-PTFEMA

*β*-CD (2 g, 1.762 mmol) was completely dissolved in 17 mL anhydrous NMP and cooled to 0 °C in an ice bath under argon. BIBB (13.72 mL, 111.006 mmol) was dissolved in anhydrous NMP (13 mL) at 0 °C and then added dropwise to the *β*-CD solution with magnetic stirring over a period of 1 h at 0 °C under argon. After this addition, the reaction temperature was maintained at 0 °C for 2 h and then warmed to ambient temperature, after which the reaction was allowed to continue for 2 days at 25 °C in a water bath. The pale-yellow solution obtained was concentrated in a vacuum oven for 24 h. The resulting syrup-like product was diluted with 100 mL CH_2_Cl_2_ and then washed sequentially with saturated NaHCO_3_ aqueous solution (3 × 100 mL) and deionized water (3 × 100 mL). The organic layers were dried over anhydrous Na_2_SO_4_ overnight. The solution collected by filtration was evaporated under reduced pressure and then crystallized in cold *n*-hexane to produce a pale-yellow precipitate. After filtering, the final product was dried in a vacuum oven at 40 °C for 24 h. A clean and dry Schlenk flask was charged with TFEMA (8.5 mL, 0.059 mol), bpy (69.7 mg, 0.446 mmol), and cyclohexanone (8.5 mL). The flask was deoxygenated by three freeze-pump-thaw cycles. The flask was filled with argon during the final cycle before CuCl (14.7 mg, 0.149 mmol) was quickly added to the frozen mixture. The flask was sealed with a glass stopper, evacuated, and back-filled with argon three times before immersion in an oil bath at 80 °C. Finally, the argon-purged multifunctional macroinitiator 21-Br-*β*-CD (30.2 mg, 7.08 mmol) was injected into the reaction system via a syringe through the side arm of the Schlenk flask. After 12 h, the Schlenk flask was cooled in an ice-water mixture and exposed to air. The resulting solution was diluted with CH_2_Cl_2_ and filtered through a column filled with neutral alumina to remove the copper complex. After the solvent was condensed by rotary evaporation, the polymer was purified by dissolution and precipitation twice with CH_2_Cl_2_ and cold n-hexane and dried at 40 °C in vacuum for 2 days.

### Characterization of 21-*β*-CD-*g*-PTFEMA

Proton nuclear magnetic resonance (^1^H NMR), carbon nuclear magnetic resonance (^13^C NMR), and fluorine nuclear magnetic resonance (^19^F NMR) spectroscopy were performed on Bruker Avance NEO 600 NMR spectrometers. Fourier Transform Infrared Spectra (FTIR) were obtained on a Bruker Vertex 70 v spectrometer at a resolution of 4 cm^−1^ (16 scans collected) in KBr pellets. Elemental analyses for C, H, and N were carried out using a Vario EL cube elemental analyser. X-ray photoelectron spectroscopy (XPS) measurements were recorded on a Thermo Scientific ESCALAB 250Xi using monochromated Kα X-rays (1486.6 eV), and all the binding energies were obtained in XPS spectra and calibrated using the C 1 *s* peak at 284.6 eV. X-ray powder diffraction (XRD) measurements were conducted using a Bruker AXS D8 diffractometer with Cu Kα radiation (λ = 0.1538 nm). The molecular weight and polydispersity of the polymer were determined by gel permeation chromatography (GPC) on a PL-GPC 220 system (Polymer Laboratory Co. Ltd., UK) equipped with two linear Mixed-B columns (column size 300 × 7.5 mm) and a refractive index detector at 40 °C, using dimethyl formamide as the eluent at a flow rate of 1.0 mL min^−1^ and poly(methyl methacrylate) as the calibration standard. Thermogravimetric analysis (TGA) was performed using a TA Instruments Q 600 thermogravimetric analyzer at a heating rate of 10 °C min^−1^ from 50 to 500 °C under nitrogen with a flow rate of 100 mL min^−1^. The powder morphology and elementary analysis were obtained by field-emission scanning electron microscopy (FE-SEM) on a Hitachi S-4800 with implemented energy-dispersive X-ray spectroscopy (EDX).

### Materials for preparing all-solid-state polymer electrolytes

Polyethylene oxide (PEO; approximate M_w_ = 600,000 g mol^−1^, Acros (Acros Organics, Geel, Belgium)), bis(trifluoromethanesulfonyl)imide lithium salt (LiTFSI, 99%, Acros (Acros Organics, Geel, Belgium)), bis(trifluoromethanesulfonyl)imide sodium salt (NaTFSI, > 98.0%, Tokyo Chemical Industry Co., Ltd. (TCI)), sodium hexafluorophosphate (NaPF_6_, 99%, Tokyo Chemical Industry Co., Ltd. (TCI)), and sodium perchlorate (NaClO_4_, 99%+, Acros (Acros Organics, Geel, Belgium)) were dried under a high vacuum (−0.1 MPa) for 24 h at 55 °C and then stored in a Schott Duran bottle in a glove box before use. Acetonitrile (99.9%, Acros (Acros Organics, Geel, Belgium)).

### Preparing solid-state polymer electrolytes

The composite polymer electrolytes for AS-LMBs and AS-SMBs were prepared through a conventional solution casting technique. For AS-LMBs, first, 21-*β*-CD-*g*-PTFEMA with different molecular weights was mixed with PEO and LiTFSI at a predetermined ratio in acetonitrile in a glove box under an argon atmosphere. All the materials were weighed by a METTLER TOLEDO ME55 with an accuracy of 0.01 mg. Second, the solution was stirred at 55 °C until all powders were dissolved completely. Then, the homogeneous solution was degassed, cast onto a Teflon mold, and left to evaporate the acetonitrile slowly for 24 h at 25 °C. Finally, the membrane was formed and dried under vacuum at 55 °C for 24 h (−0.1 MPa) to completely remove residual acetonitrile. The thickness of the membrane was measured by a thickness gauge and determined to be ~200 μm. The ratios of 21-*β*-CD-*g*-PTFEMA, PEO, and LiTFSI were applied according to the orthogonal experimental design for AS-LMBs. For AS-SMBs, the preparation procedures of the composite polymer electrolyte were identical to those for AS-LMBs. For the design of the orthogonal experiment for AS-SMBs, in addition to the same factors as the AS-LMBs, another influencing factor, the type of sodium salt (NaTFSI, NaClO_4,_ and NaPF_6_), was also introduced.

### Orthogonal experimental design for FMC-ASPE-Li and FMC-ASPE-Na

Based on the orthogonal test method for FMC-ASPE-Li preparation, the studied factors included the molecular weight of 21-*β*-CD-*g*-PTFEMA (A), the mass ratio of PEO-to-21-*β*-CD-*g*-PTFEMA (B), and the mass ratio of LiTFSI to polymer (m_polymer_ = m_PEO_ + m_21-*β*-CD-*g*-PTFEMA_) (C), where two levels for each factor were selected. By designing the L4 (2^3^) orthogonal test, the number of experiments was effectively reduced to four compared to eight experiments for the exhaustive method, as shown in Supplementary Fig. 12. The factors and levels for the orthogonal test are listed in Table 1. Polycrystalline NMC622 was chosen as thecathode material to further rapidly establish the optimal conditions because its surface area can accelerate impedance growth and capacity decay. Based on this approach, CR2032-type coin cells were assembled with a Li metal anode, polycrystalline NMC622, and FMC-ASPE-Li prepared according to the orthogonal test design. The capacity retention after 30 cycles was used as the criterion for screening the optimal level. The pooled capacity retention results are summarized in Supplementary Figs. 13–21 and Supplementary Table 2; these results are also combined with the evaluation indices of the orthogonal L4 (2^3^) test analysis and shown in Supplementary Fig. 23 and Table 1. Based on these analyses, the optimal level was confirmed to be A_2_B_2_C_1_, and the order of importance was determined to be A > B > C. In addition to retaining the same factors as the orthogonal experimental design for AS-LMBs, another factor, the type of salt, was also introduced in preparation for the FMC-ASPE-Na. Concomitantly, the number of levels for each factor increased to three. A more integrated L9 (3^4^) orthogonal array was designed with four factors, and three levels are listed in Table 2. Only nine groups of experiments should be performed directed by an orthogonal array, but 81 groups of experiments must be carried out using an exhaustive method. To investigate the influence of FMC-ASPE-Na on the coulombic efficiency of Na-metal anodes, NCNFM|FMC-ASPE-Na|Na full cells were tested. By combining the analysis of the results with evaluation indices of the orthogonal L9 (3^4^) test, which are summarized in Table 2 Supplementary Table 3, the optimal level was A_3_B_2_C_3_D_3_, and the order of importance was C > D > B > A.

### Characterization of solid-state polymer electrolytes

Attenuated total reflection-Fourier transform infrared spectroscopy (ATR-FTIR) was performed on a Bruker Vertex 70 v spectrometer to characterize the presence of specific chemical groups in the polymer electrolyte films. X-ray photoelectron spectroscopy (XPS) measurements were recorded on a Thermo Scientific ESCALAB 250Xi using monochromated Kα X-rays (1486.6 eV), and all the binding energies were obtained in XPS spectra were calibrated using the C 1 *s* peak at 284.6 eV. X-ray powder diffraction (XRD) measurements were conducted using a Bruker AXS D8 diffractometer with Cu Kα radiation (λ = 0.1538 nm). Field-emission scanning electron microscopy (FE-SEM) on a Hitachi S-4800 with implemented energy-dispersive X-ray spectroscopy (EDX) was used to provide information on the surface and cross-section of polymer electrolyte films. Thermogravimetric analysis (TGA) was performed using a TA Instruments Q 600 thermogravimetric analyzer at a heating rate of 10 °C min^−1^ from 50 to 500 °C under nitrogen with a flow rate of 100 mL min^−1^. Thermogravimetry-Fourier transform infrared spectroscopy (TG-FTIR) analysis was carried out under a nitrogen atmosphere from 40 to 800 °C at a heating rate of 10 °C min^−1^ by using a PerkinElmer STA 6000 thermal analyzer. X-ray tomography was tested using a Bruker Skyscan 1272 (Germany). The voltage was set as 25 kV and the current as 190 μA. The resolution of the X-ray tomography was 1 μm. The CTAn and CTVox software were used for the analysis and image rendering.

### Materials for electrode preparation and coin cell assembly

Poly(vinylidene fluoride) (PVDF; approximate M_w_ = 534,000 g mol^−1^, Sigma-Aldrich) was dried under a high vacuum (−0.1 MPa) for 24 h at 55 °C before use. 1-Methyl-2-pyrrolidinone (NMP; 99.5%, Alfa Aesar (Alfa Aesar, Wardhill, MA)), conductive carbon black (Super-P, TIMCAL), LiMn_0.6_Fe_0.4_PO_4_C_0.251_ (LMFP, Guangdong Canrd New Energy Technology Co., Ltd.), polycrystalline LiNi_0.6_Mn_0.2_Co_0.2_O_2_ (PC-NMC622, Beijing Welion New Energy Technology Co., Ltd.), single-crystal LiNi_0.6_Mn_0.2_Co_0.2_O_2_ (SC-NMC622, Beijing Welion New Energy Technology Co., Ltd.), single-crystal LiNi_0.8_Mn_0.1_Co_0.1_O_2_ (SC-NMC811, Hefei KeJing Materials Technology Co., Ltd.), LiFePO_4_ (LFP, Beijing Welion New Energy Technology Co., Ltd.), NaCu_1/9_Ni_2/9_Fe_1/3_Mn_1/3_O_2_ (NCNFM, HiNa Battery Technology Co., Ltd.), Na_3_(VOPO_4_)_2_F (NVOPF, synthesized by sol-gel methods), Na_3_V_2_(PO_4_)_3_ (NVP, Hefei KeJing Materials Technology Co., Ltd.) were used as cathode materials. Lithium metal foil (99.95%, 0.25 mm thickness, *Φ*12 mm, China Energy Lithium Co., Ltd.) and sodium dry sticks (99%, Alfa Aesar (Alfa Aesar, Wardhill, MA)) were stored in a glove box under an argon atmosphere and used without further purification. The dry sodium rod was carefully cut off the protective layer coated on the surface to expose the fresh sodium layer. A small piece of sodium was rolled into a sodium foil with a thickness of 0.25 mm and then punched into circular sodium foils with a diameter of 12 mm before use.

### Preparing electrodes

The binders were added to NMP to prepare a uniform polymer binder solution with a concentration of 0.5 g mL^−1^ and stored in a dry box before use. The electrodes were prepared using active materials, Super-P, and polymer binder in a predetermined weight ratio of 8: 1: 1. First, the active material and Super-P were added into a mortar and ground for 1 h. Second, the preprepared polymer binder solution was added and ground for 30 min to make a uniform slurry. The slurry was coated on a flat, clean aluminum foil (≥99.35%, 0.16 mm thickness, Hefei KeJing Materials Technology Co., Ltd.) with a doctor blade and dried in a convection oven at 55 °C for 6 h. The loading mass of the active material on Al foil was typically 1–2 mg cm^−2^ if not specifically stated. A higher loading mass of 3–4 mg cm^−2^ was also applied, labeled in the corresponding figure captions. Then, the electrode sheets were punched into circular electrodes with a diameter of 8 mm and dried again overnight at 120 °C in a vacuum oven to remove residual solvents and moisture. The thickness of the dry electrodes was approximately 30–60 μm, varying from different cathode materials and loading masses.

### Characterization of electrochemical performance

All configurations of CR2032-type coin cells were assembled in an argon-filled glove box (H_2_O < 0.1 ppm, O_2_ < 0.1 ppm).

#### Ionic conductivity

The lithium ionic conductivities and sodium ionic conductivities of the polymer electrolytes were measured by electrochemical impedance spectroscopy (EIS) at the open circuit potential using a perturbation signal of 10 mV (potentiostatic mode) in the frequency range of 4 to 100 MHz (68 data points) by a Zahner IM6e from 25 to 80 °C in a climate chamber (ESPEC MT3065, temperature accuracy <0.1 °C). The polymer electrolyte was sandwiched between two stainless steel (SS, 16 mm) blocking electrodes with an SS|SPE|SS configuration. Before the conductivity measurements, the coin cells were first heated at 80 °C for 12 h to form a stable contact and were kept at each test temperature (from 25 to 80 °C) for 2 h to reach thermal equilibrium in a climate chamber. The ionic conductivity (*σ*) was calculated according to the following Eq. (1):

1 $$\sigma =\frac{L}{S\cdot R}$$

where *L* (cm) represents the thickness of the polymer electrolyte, *S* (cm^2^) symbolizes the area of the polymer electrolyte, and *R* (Ω) represents the bulk ohmic resistance obtained by EIS.

#### Ion transference number

The lithium-ion transference number (*t*_Li_^+^) of the polymer electrolyte was derived by combining AC impedance and DC polarization techniques using Li|ASPE-Li|Li and a symmetrical coin cell at 70 °C in a climate chamber. Before measurement, the coin cell was first heated at 70 °C for 12 h to form a stable interface between the polymer electrolyte and electrode. EIS before and after polarization was acquired at the open circuit potential using a perturbation signal of 10 mV in the frequency range of 4 to 100 MHz, with the same procedure as the ionic conductivity test. Impedance data were fitted and analyzed by using the electrochemical impedance software Zview (Scribner Associates Inc.) in the frequency range of 2 MHz to 10 Hz. *t*_Li_^+^ was calculated according to the following Eq. (2):

2 $${t}_{{{{{{{\rm{Li}}}}}}}^{+}}=\frac{{I}_{{{{{{\rm{s}}}}}}}R{b}_{{{{{{\rm{s}}}}}}}(\varDelta V-{I}_{{{{{{\rm{o}}}}}}}{R}_{{{{{{\rm{o}}}}}}})}{{I}_{{{{{{\rm{o}}}}}}}R{b}_{{{{{{\rm{o}}}}}}}(\varDelta V-{I}_{{{{{{\rm{s}}}}}}}{R}_{{{{{{\rm{s}}}}}}})}$$

where Δ*V* is the applied polarization voltage of 10 mV; *I*_o_ and *I*_s_ represent the current before and after polarization; *R*_o_ and *R*_s_ stand for the initial and final impedance of the cell of the polarization process, respectively; and *Rb*_o_ and *Rb*_s_ are the initial and final resistances of the electrolyte.

The measurement procedure for the sodium-ion transference number (*t*_Na_^+^) of ASPE is the same as that for the *t*_Li_^+^ of the polymer electrolyte except for the temperature of the measurement. First, the coin cell was heated at 80 °C for 12 h to form a stable interface between the polymer electrolyte and electrode in a climate chamber.

#### Electrochemical window

The electrochemical stability of the polymer electrolyte for AS-LMBs and AS-SMBs was investigated by linear sweep voltammetry (LSV) on a Chenhua CHI800D electrochemical workstation (Shanghai, China). The Li|ASPE-Li|SS coin cell was measured over a positive potential range from open circuit potential to 6.0 V at a scan rate of 0.1 mV s^−1^ at 70 °C in a climate chamber. The Na|ASPE-Na|SS coin cell was measured at 80 °C in a climate chamber, and the measurement procedure for AS-SMBs was the same as that for the AS-LMBs.

#### Symmetrical cells

The interfacial characteristics of the polymer electrolyte and lithium (sodium) anode were detected by assembling a symmetrical coin cell configuration of Li|ASPE-Li|Li (Na|ASPE-Na|Na) and estimated using a LAND battery system (CT3001A, LANHE, China) at a constant current density of 0.1 mA cm^−2^ for 0.5 h charging and discharging. The test temperature was 70 °C for AS-LMBs and 80 °C for AS-SMBs in a climate chamber. The lithium dendrite growth inside the symmetrical cells was tested using X-ray tomography (Bruker Skyscan 1272, Germany). The authors would like to thank Ding Hao from Shiyanjia Lab (www.shiyanjia.com) for the X-ray tomography.

#### Cycling Performance

The cycling performance was conducted on a LAND CT3001A testing system using the LMFP|ASPE-Li|Li coin cell within a potential range of 2.5–4.2 V (vs. Li^+^/Li), the NMC622|ASPE-Li|Li and NMC811|ASPE-Li|Li coin cells both within a potential range of 2.7–4.2 V (vs. Li^+^/Li), the LFP|ASPE-Li|Li coin cell within a potential range of 2.5–3.8 V (vs. Li^+^/Li), the NCNFM|ASPE-Na|Na coin cell within a potential range of 2.0–4.0 V (vs. Na^+^/Na), NVOPF|ASPE-Na|Na coin cell within a potential range of 2.5–4.2 V (vs. Na^+^/Na), and the NVP|ASPE-Na|Na coin cell both within a potential range of 2.5–3.8 V (vs. Na^+^/Na). Before measurement, each coin cell was first heated at 70 °C (vs. Li^+^/Li) or 80 °C (vs. Na^+^/Na) for 10 h to form a stable interface between the polymer electrolyte and electrodes. The temperature of testing was 70 °C (vs. Li^+^/Li) or 80 °C (vs. Na^+^/Na) in a climate chamber. The specific current for the cycling test refers to the mass of the active materials. The thickness of the polymeric electrolytes was ~160 μm. The average discharge voltage was calculated by dividing the discharge energy by discharge capacity.

### Materials for electrode preparation of LATP-coated SC-NMC622 and coin cell assembly

Poly(vinylidene fluoride) (PVDF; approximate M_w_ = 534,000 g mol^−1^, Sigma-Aldrich) was dried under a high vacuum (−0.1 MPa) for 24 h at 55 °C before use. 1-Methyl-2-pyrrolidinone (NMP; 99.5%, Alfa Aesar (Alfa Aesar, Wardhill, MA)), conductive carbon black (Super-P, TIMCAL), single-crystal LiNi_0.6_Mn_0.2_Co_0.2_O_2_ (SC-NMC622, Beijing Welion New Energy Technology Co., Ltd.), Li_1.5_Al_0.5_Ti_1.5_(PO_4_)_3_ (LATP; 99%, Beijing Welion New Energy Technology Co., Ltd.) and lithium metal foil (99.95%, 0.25 mm thickness, *Φ*12 mm, China Energy Lithium Co., Ltd.) were stored in a glove box under an argon atmosphere and used without further purification.

### Synthesis of LATP-coated SC-NMC622

First, the LATP was ground by a pulverizer to reduce the particle size to hundreds of nanometers. Then, 1 wt% LATP and SC-NMC622 powder were added to the mortar. After grinding for 1 h, the resulting precursor was heated in air at 600 °C for 4 h to obtain the final LATP-modified SC-NMC622 material (denoted as LATP@SC-NMC622).

### Electrode preparation of LATP-coated SC-NMC622

PVDF was added to NMP to prepare a uniform PVDF binder solution with a concentration of 0.5 g mL^−1^ and stored in a dry box before use. The LATP-coated SC-NMC622 electrode was prepared by mixing active materials, Super-P, and polymer binder in a weight ratio of 8:1:1. First, the LATP-coated SC-NMC622 material and Super-P were added into a mortar and ground for 1 h. Second, the preprepared PVDF binder solution was added and ground for 30 min to make a uniform slurry. The slurry was coated on a flat, clean aluminum foil with a doctor blade and dried in a convection oven at 55 °C for 6 h. Then, the electrode sheets were punched into circular electrodes with a diameter of 8 mm and dried overnight at 120 °C in a vacuum oven to remove residual solvents and moisture. The thickness of the dry electrode was ~50 μm.

### Materials for fabricating pouch cell

Poly(vinylidene fluoride) (PVDF; approximate M_w_ = 534,000 g mol^−1^, Sigma-Aldrich) was dried under a high vacuum (−0.1 MPa) for 24 h at 55 °C before use. 1-Methyl-2-pyrrolidinone (NMP; 99.5%, Alfa Aesar (Alfa Aesar, Wardhill, MA)), conductive carbon black (Super-P, TIMCAL), LiMn_0.6_Fe_0.4_PO_4_ (LMFP, Guangdong Canrd New Energy Technology Co., Ltd.), lithium foil (99.95%, China Energy Lithium Co., Ltd.), copper foil (25-μm thickness, Shenzhen Kejing Star Technology Co. Ltd.).

### Fabrication and test of pouch cell

For the cathode side, the LMFP cathode was chosen for assembling the pouch cell (cathode material: carbon: binder = 8: 1: 1 in weight). The electrode sheets of LMFP were cut into a predetermined size, as shown in Supplementary Fig. 58. The effective area was 14 cm^2^ (4 cm × 3.5 cm). The preparation of the polymer electrolyte was the same as the preparation method mentioned above, and the optimal composition was selected as A_2_B_2_C_1_ according to the orthogonal experimental results. The ASPE membrane was cut into a rectangle of 5.5 cm × 5.5 cm, as shown in Supplementary Fig. 58. The mass loading of the electrode was about 1.4 mg cm^−2^. The thickness of the dry electrodes was ~40 μm. The thickness of the polymeric electrolyte was about ~150 μm. For the anode side, ultrathin Li films (4.7 cm × 4.5 cm rectangle with a thickness of approximately 200 μm) obtained by physically pressing pristine Cu foil (25 μm) were employed as anodes for LMFP|FMC-ASPE-Li|Li pouch cells. The pouch cells were assembled and sealed in a laminated aluminum film bag according to the schematics in Supplementary Fig. 57a. The cycling performance of the LMFP|FMC-ASPE-Li|Li pouch cell was conducted on a LAND CT3001A testing system within a potential range of 2.5–4.2 V (vs. Li^+^/Li) at a test temperature of 70 °C in a climate chamber under external pressure (the initial pressure applied was 0.63 × 10^−3^ MPa, and the increased pressure after tightening the screws was 0.28 MPa.). The fixture consists of two smooth stainless steel plates (10 cm × 10 cm) and four long screws (with nuts), which could be used to exert and adjust the pressure on the pouch cell by adjusting the distance between the two plates. Before the cycling test, the pouch cell was first heated at 70 °C for 10 h to form a stable interface between the polymer electrolyte and electrode and then cycled at 70 °C in a climate chamber. After cycling, the LMFP|FMC-ASPE-Li|Li pouch cell was connected to a LED by fine aluminum wires at 25 °C, as shown in Supplementary Fig. 57c, d. The LED was fixed in a lantern made of FMC-ASPE-Li membrane (Supplementary Fig. 57b) and raised by an astronaut model.

### Finite-element simulation of ASPEs for AS-LMBs and AS-SMBs

The macrohomogeneous model was used to investigate the salt distribution in both lithium metal symmetrical cells (Li|PEO-ASPE-Li|Li, Li|FMC-ASPE-Li|Li) and Na-metal symmetrical cells (Na|PEO-ASPE-Na|Na, Na|FMC-ASPE-Na|Na). Focusing on the transient nature of the distribution, we started with a material balance Eq. (3) for components inside the electrolyte, assuming no production:

3 $$\frac{\partial {c}_{{{{{{\rm{i}}}}}}}}{\partial t}=-\nabla \cdot {N}_{{{{{{\rm{i}}}}}}}$$

where *c*_i_ is the concentration of species and *N*_*i*_ is the flux density of species. In an electrolyte system, the flux density can be described with migration, diffusion, and convection terms, as shown in Eq. (4):

4 $${N}_{{{{{{\rm{i}}}}}}}=-{z}_{{{{{{\rm{i}}}}}}}{u}_{{{{{{\rm{i}}}}}}}{{{{{\rm{F}}}}}}{c}_{{{{{{\rm{i}}}}}}}\nabla \varPhi -{D}_{{{{{{\rm{i}}}}}}}\nabla {c}_{{{{{{\rm{i}}}}}}}+{c}_{{{{{{\rm{i}}}}}}}v$$

where *z*_i_ is the charge number of the species, *u*_i_ is the mobility of the species, F is Faraday’s constant, *Φ* is the potential, *D*_i_ is the diffusion coefficient of the species, and *v* is the fluid velocity. With the electroneutrality equation, the salt concentration profile Eq. (5) along the x-direction in the absence of convection is given by

5 $$\frac{{{{{{\rm{d}}}}}}c}{{{{{{\rm{d}}}}}}t}=\frac{{{{{{\rm{d}}}}}}}{{{{{{\rm{d}}}}}}x}\left(D\frac{{{{{{\rm{d}}}}}}c}{{{{{{\rm{d}}}}}}x}\right)-\frac{i{{{{{\rm{d}}}}}}{t}^{+}}{{{{{{\rm{Fd}}}}}}x}$$

with two boundary conditions, as shown in Eqs. (6) and (7):

6 $$-D{\frac{\partial c}{\partial x}\Big|}_{x=0}=\frac{1-{t}^{+}}{{{{{{\rm{F}}}}}}}i$$

7 $$-D{\frac{\partial c}{\partial x}\Big|}_{x={{{{{\rm{L}}}}}}}=\frac{1-{t}^{+}}{{{{{{\rm{F}}}}}}}i$$

where *D* is the Fickian diffusion coefficient, *i* is the current density, and *t*^+^ is the transference number. This work was solved using a Li metal battery (Na-metal battery) interface in COMSOL Multiphysics. We applied a voltage of 10 mV for all cells for 30 s. The solid electrolyte thickness and width were set to 200 μm and 50 µm for all cells. The initial salt concentration was set to 500 mol m^−3^ for AS-LMBs and mol m^−3^ for SMBs. The diffusion coefficients were calculated by using true materials and are summarized in Supplementary Tables 15–18. The Nernst–Einstein Eq. (8) calculates the ionic conductivity:

8 $$\sigma =D\frac{n{q}^{2}}{{{{{{\rm{K}}}}}}T}$$

where K is the Boltzmann constant, *q* is the charge of the charge carriers, *D* is the diffusion coefficient, and *T* is the temperature. The following Eq. (9) can distinguish the cation and anion diffusion coefficients:

9 $${t}_{{{{{{\rm{cation}}}}}}}=\frac{{\sigma }_{{{{{{\rm{cation}}}}}}}}{\sigma }$$

### DFT calculations

The Gaussian 16 program was employed for DFT calculations. Geometry optimization was performed at the B3LYP-D3BJ/6-311 G(d,p) level, and the frontier orbital levels and isosurfaces were obtained at the same level.

All first-principles calculations were performed within the Vienna Ab Initio Simulation Package (VASP)^45,46^ based on density functional theory (DFT). The projected augmented wave (PAW)^47^ potentials were used to address the electronic exchange–correlation interaction along with the GGA functional in the parameterization of the Perdew Burke and Ernzerhof (PBE) pseudopotential^48^. A plane wave representation for the wave function with cut-off energy of 500 eV was applied. Geometry optimizations were performed using conjugate gradient minimization until all the forces acting on ions were less than 0.01 eV Å^−1^ per atom. The K-point mesh with a spacing of *ca*. 0.03 Å^−1^ was adopted. Crystal structures were built using VESTA software^49^. The PEO (C_5_H_12_O_3_) and PTFEMA (C_6_H_9_O_2_F_3_) molecules were placed in the unit cell with *a* = 20 Å, *b* = 10 Å, and *c* = 10 Å for geometry optimizations. The hydrogen bond between PEO (C_5_H_12_O_3_) and PTFEMA (C_6_H_9_O_2_F_3_) was calculated in the unit cell with *a* = 20 Å, *b* = 20 Å, and *c* = 20 Å. The adsorption energy *E*(Li) of Li is defined as Eq. (10):

10 $$E({{{{{\rm{Li}}}}}})=E({{{{{\rm{M}}}}}}+{{{{{\rm{Li}}}}}})-{{{{{\rm{E}}}}}}({{{{{\rm{M}}}}}})-E({{{{{\rm{Li}}}}}})$$

where *E*(M + Li), *E*(M), and *E*(Li) are the energies of PEO-Li or PTFEMA-Li, PEO or PTFEMA, and a single Li atom, respectively.

The energy of the hydrogen bond is calculated using Eq. (11):

11 $$E({{{{{\rm{hydrogen}}}}}}\,{{{{{\rm{bond}}}}}})=E({{{{{\rm{M1}}}}}}+{{{{{\rm{M2}}}}}})-E({{{{{\rm{M1}}}}}})-E({{{{{\rm{M2}}}}}})$$

where *E*(M1 + M2), *E*(M1), and *E*(M2) are the energies of PEO-PTFEMA, PEO, and PTFEMA, respectively.

## Supplementary information

Supplementary Information

## Data Availability

The data that support the plots within this paper and other findings of this study are available from the corresponding author on reasonable request. Source data are provided with this paper.

## Source data

Source Data
