# Supplementary material for: Rational design of a topological polymeric solid electrolyte for high-performance all-solid-state alkali metal batteries
Source: Nat Commun. 2022 Jul 19;13:4181. doi: 10.1038/s41467-022-31792-5 (PMC9296621; doi:10.1038/s41467-022-31792-5)
Supplement: Supplementary file 1 — Supplementary Information [file 41467_2022_31792_MOESM1_ESM.pdf]

Supplementary Information

Rational design of a topological polymeric solid electrolyte for high-performance all-solid-state alkali metal batteries

Yun Su[1,2,3], Xiaohui Rong[2,3,4,5*], Ang Gao[2,4], Yuan Liu[2,4], Jianwei Li[6], Minglei Mao[2,5], Xingguo Qi[2], Guoliang Chai[6], Qinghua Zhang[2,3], Liumin Suo[2,3,4,5], Lin Gu[2,4], Hong Li[2,3,4,5], Xuejie Huang[2,3,4,5], Liquan Chen[2,3,4,5], Binyuan Liu[1,7*] and Yong-Sheng Hu[2,3,4,5,*]

[1] *Hebei Key Laboratory of Functional Polymer, School of Chemical Engineering and Technology, Hebei University of Technology, Tianjin 300130, China.*

[2] *Beijing National Laboratory for Condensed Matter Physics, Institute of Physics, Chinese Academy of Sciences, Beijing 100190, China.*

[3] *Yangtze River Delta Physics Research Center Co. Ltd, Liyang 213300, China.*

[4] *Center of Materials Science and Optoelectronics Engineering, University of Chinese Academy of Sciences, Beijing 100190, China.*

[5] *Huairou Division, Institute of Physics, Chinese Academy of Sciences, Beijing 101400, China.*

[6] *State Key Laboratory of Structural Chemistry, Fujian Institute of Research on the Structure of Matter, Chinese Academy of Sciences, Fuzhou 350002, China.*

[7] *Key Laboratory for Green Processing of Chemical Engineering of Xinjiang Bingtuan, School of Chemistry and Chemical Engineering, Shihezi University, Shihezi 832003, China.*

* Corresponding authors and E-mail address:
X.R., rong@iphy.ac.cn; B.L., byliu@hebut.edu.cn; Y.-S.H., yshu@iphy.ac.cn.

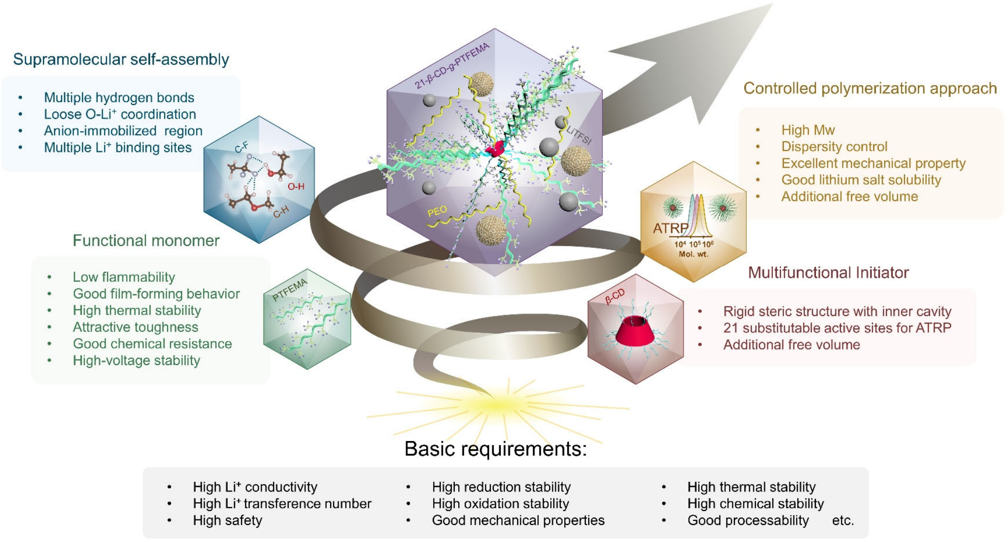

Supplementary Figure 1 The design principles of this work. The abbreviation lists are in Supplementary Note 1.

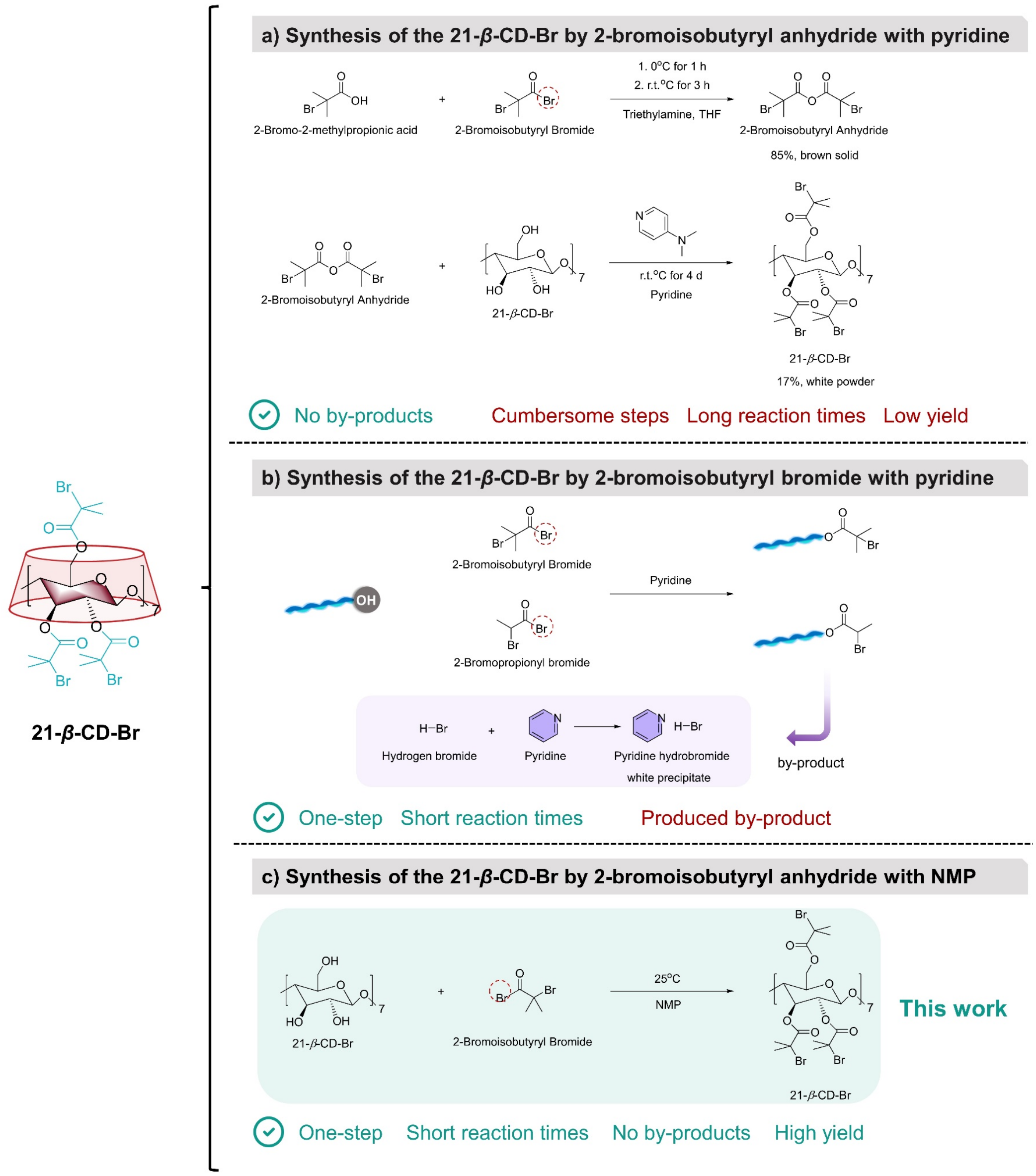

Supplementary Figure 2 Comparison of different synthesis routes to obtain 21-*β*-CD-Br. In light of Ohno et al. [1]. first reported the synthetic route of 21-*β*-CD-Br using a *β*-CD-based multifunctional macroinitiator, and up to 21 arms could be created in star polymers. Nevertheless, the cumbersome steps, long reaction times, and low yield only 17% have limited the large-scale preparation and application of *β*-CD-based star polymers with 21 arms. Compared with Ohno's method, the multifunctional macroinitiator containing 21 initiating sites obtained by using BIBB to directly modify the hydroxyl groups of *β*-CD showed a significant improvement in efficiency[2], while the challenge shifted to the choice of solvent for the synthesis. Since pyridine is a suitable solvent for β-CD, it is immiscible with BIBB, inducing white pyridine-HBr salt precipitation[3]. To address the weakness of solvent dissolution of BIBB and to inhibit the formation of byproducts, a critical solvent needs to be identified. In this work, the polar aprotic solvent NMP was selected as an optimized solvent, and 21-*β*-CD-Br with high efficiency, high yield, and high purity was synthesized through the esterification reaction of *β*-CD and BIBB in a one-step method[4].

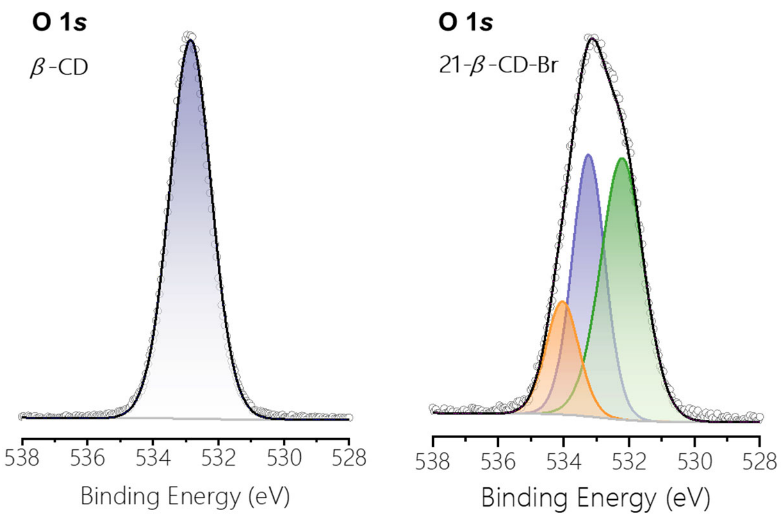

Supplementary Figure 3 The O 1*s* XPS spectra of *β*-CD and 21-*β*-CD-Br. The overall XPS spectra of *β*-CD and 21-*β*-CD-Br are shown in Figure 1b(I), and a new peak centre is identified at approximately 71.5 eV for Br 3*d*. Furthermore, the Br 3*d*-binding energies (BEs) of 21-*β*-CD-Br are 69.9 and 71.1 eV, corresponding to Br $3d_{5/2}$ and Br $3d_{3/2}$, respectively (Figure 1b(II)). As displayed in Figure .1b(III), the C 1*s* core level spectrum of *β*-CD can be deconvoluted by four peaks positioned at approximately 284.7, 286.4, and 287.9 eV, which are attributable to the C-H/C-C, C-O, and O-C-O bonds, respectively. The C 1s core level spectrum of 21-*β*-CD-Br can be curve-fitted by four peak components with BEs at approximately 285.0, 286.6, 287.9, and 288.9 eV, revealing the existence of C-H/C-C, C-O/C-Br, O-C-O, and O=C-O species, respectively, as shown in Figure 1b(IV). The conspicuous O=C-O peak is assigned to the ester bonds linking the *β*-CD ring and the bromoisobutyryl units, while the O-C-O peak is obviously weakened, which is attributed to *β*-CD. By comparing the integration between the two species, the degree of substitution can also be calculated.

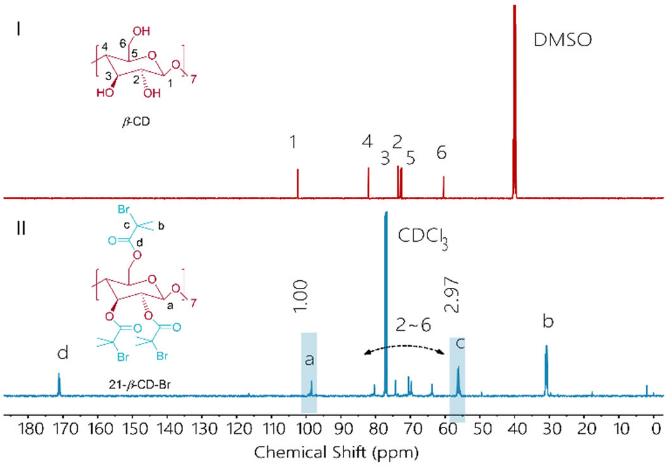

Supplementary Figure 4 ^{13}C NMR spectra of *β*-CD and 21-*β*-CD-Br. The peaks at δ = 98.48 ppm (a, 7C, -O-*CH*-O- of sugar carbons) mainly associate with the inner carbon between the oxygen moieties and oxygen moieties on the glucose units of *β*-CD, and the peaks at δ = 63.79, 69.70-74.25 and 80.25 ppm should be attributed to the residual carbons of sugar. The peak at δ = 29.66-31.25 ppm (b, 42C, α-CH_3) demonstrates the presence of 2-bromoisobutyryl groups, the peak at 56.6 ppm is associated with the carbon adjacent to the bromine (c, 21C, -*C*$(CH_3)_2$Br), and the peak located at 171.2 ppm is attributed to the carbonyl groups (d, 21C, -*C*=*O*-). The hydroxyl group conversion can also be calculated by using the following equation (1):

$$C_{\mathrm{OH}}=\frac{I\mathrm{A_c}}{3I\mathrm{A_a}}\times 100\% \qquad (1)$$

where C_{OH} is the conversion efficiency of hydroxyl groups on *β*-CD; $I\mathrm{A_c}$ and $I\mathrm{A_a}$ represent the integral area of the carbon adjacent to bromine (c, 21C, -*C*$(CH_3)_2$Br) and the integral area of the carbon associated with the inner carbon between the oxygen moieties and oxygen moieties on the glucose units of *β*-CD (a, 7C, -O-*CH*-O- of sugar carbons), respectively. By calculating the area ratio of peak C_c and peak C_a, the degree of substitution of the hydroxyl groups on the outside surface per *β*-CD unit was determined to be approximately 21. The ^{13}C NMR results further indicate that a *β*-CD core with 21 alkyl halide initiation sites was precisely synthesized.

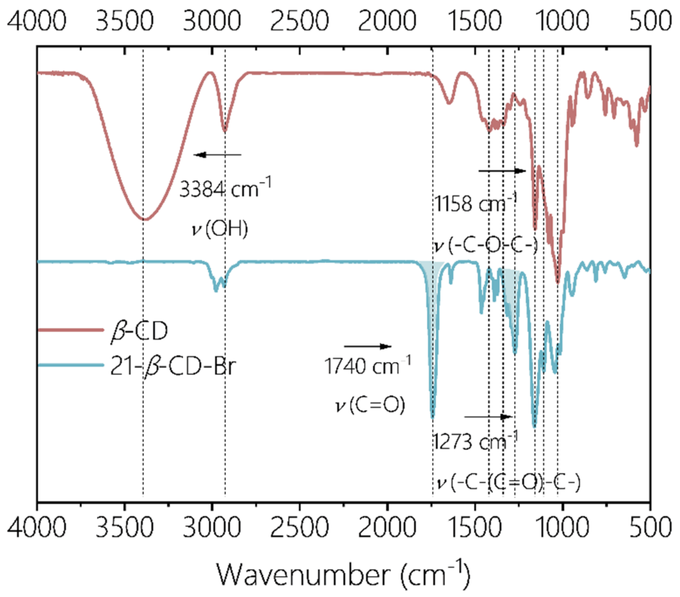

Supplementary Figure 5 FTIR spectra of *β*-CD and 21-*β*-CD-Br. The signals at 3384 cm^{-1} and 1158 cm^{-1} in the spectrum of *β*-CD are assigned to the stretching frequency of hydroxyl groups (*ν*O-H) and deformation of C-O-C groups, respectively. The signals at 1419 cm^{-1} and 1339 cm^{-1} come from O-H deformation of primary and secondary alcohols. The characteristic peak at 2930 cm^{-1} is attributed to the asymmetric C-H stretching vibration of CH_2 groups, and the absorptions at approximately 1465 cm^{-1} and 1385 cm^{-1} originate from asymmetric C-H and symmetric C-H bending of -CH_2 groups, respectively. The appearance of the absorption peaks at 1740 cm^{-1} and 1274 cm^{-1} in the spectrum of 21-*β*-CD-Br corresponded to the stretching frequency of carbonyl groups (*ν*C=O) and asymmetric vibration of C-(C=O)-O, which further indicated the formation of the star-shaped multifunctional macroinitiator 21-*β*-CD-Br.

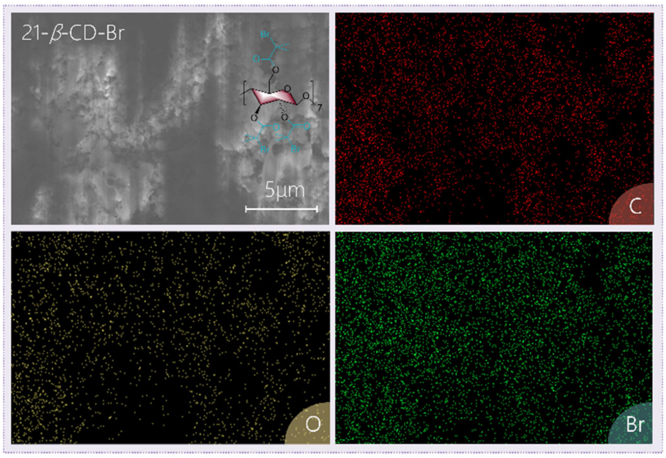

Supplementary Figure 6 SEM-EDS of 21-*β*-CD-Br.

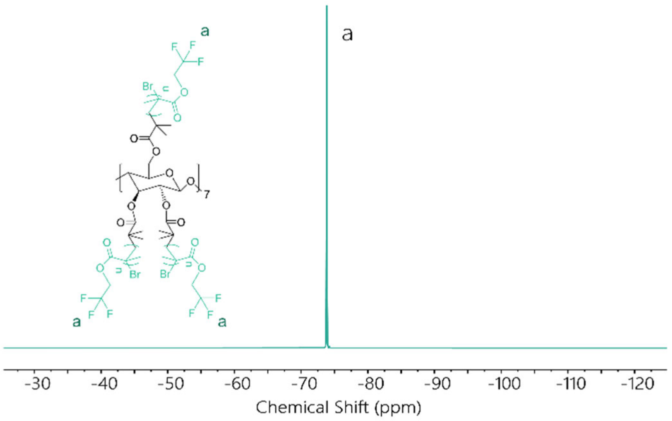

Supplementary Figure 7 ^{19}**F NMR spectrum of 21-*β*-CD-*g*-PTFEMA.**

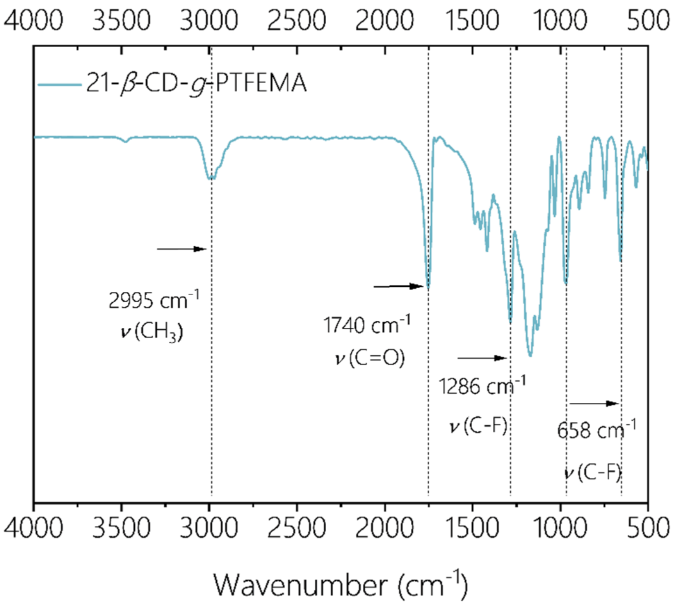

Supplementary Figure 8 FTIR spectrum of 21-*β*-CD-*g*-PTFEMA.

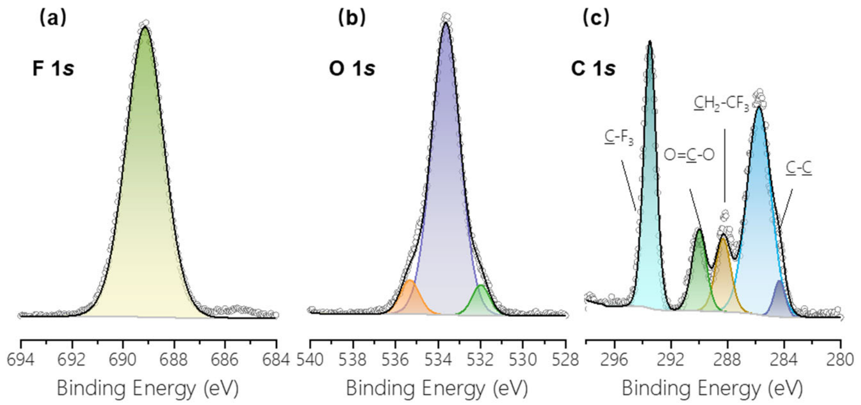

Supplementary Figure 9 F 1*s*, O 1*s* and C 1*s* XPS spectra of 21-*β*-CD-*g*-PTFEMA.

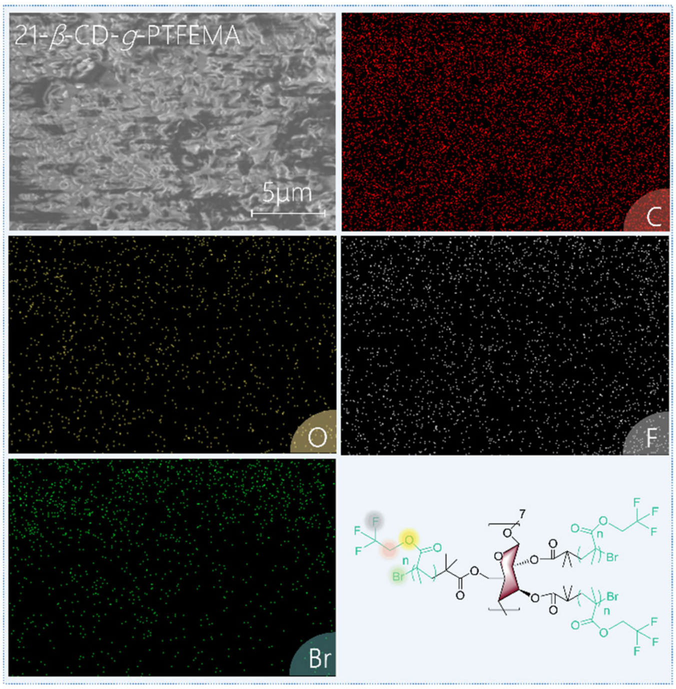

Supplementary Figure 10 SEM-EDS of 21-*β*-CD-*g*-PTFEMA.

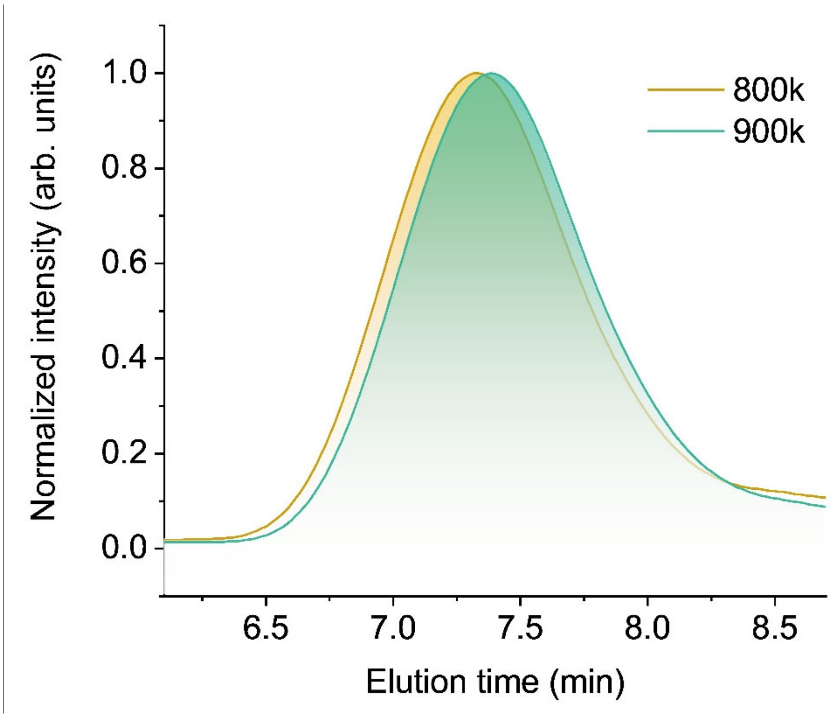

Supplementary Figure 11 GPC results of 21-*β*-CD-*g*-PTFEMA.

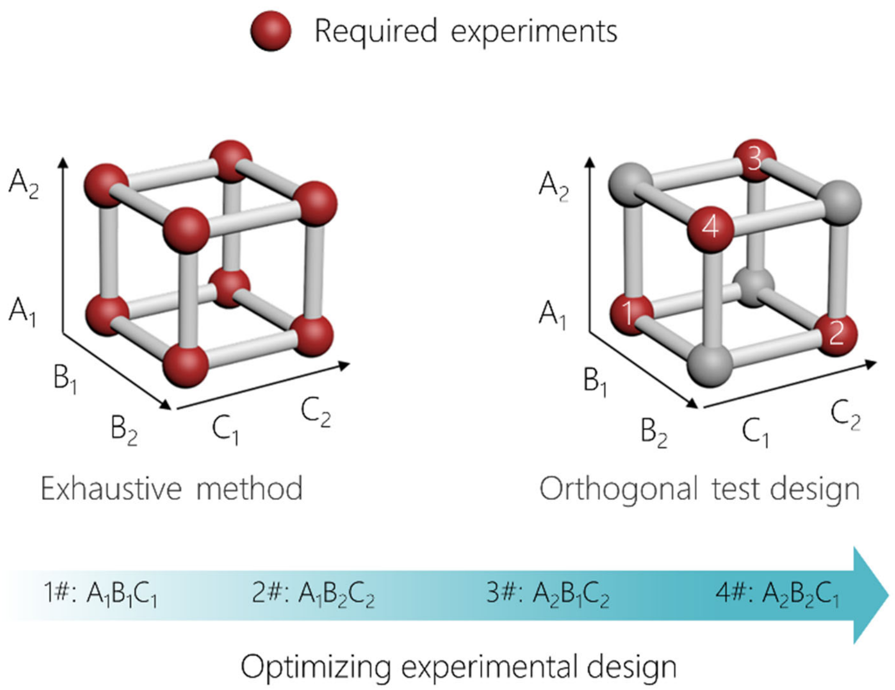

Supplementary Figure 12 The orthogonal test method used for FMC-ASPE-Li with an L4 (2^3) orthogonal array.

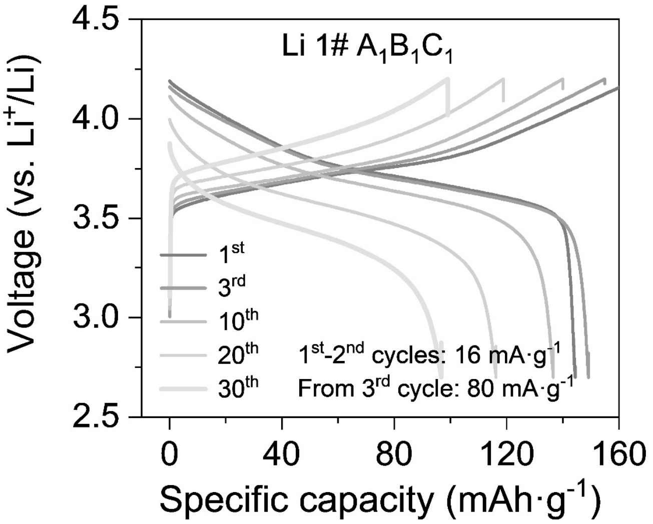

Supplementary Figure 13 Charge/discharge voltage profiles of the experiment 1 selected by the orthogonal experimental design (L_4 (2^3)) for FMC-ASPE-Li (CR2032 coin cell, 70°C, 16 mA·g^{-1} for the first two cycles, 80 mA·g^{-1} from the 3rd cycle, 2.7-4.2 V, FMC-ASPE-Li composition: m_{LiTFSI} : m_{PEO} : $m_{21\text{-}\beta\text{-CD-}g\text{-PTFEMA}}$ = 1 : 4 : 1, $M_{n,\ GPC\ (21\text{-}\beta\text{-CD-}g\text{-PTFEMA})}$ = 800k, M_{PEO} = 600k).

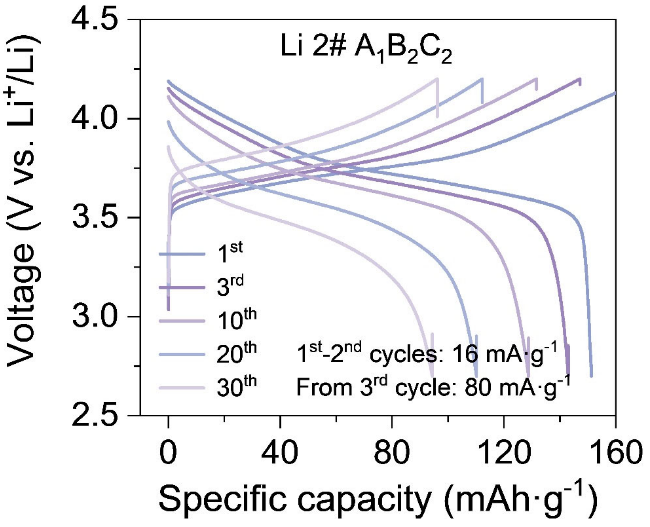

Supplementary Figure 14 Charge/discharge voltage profiles of the experiment 2 selected by the orthogonal experimental design (L_4 (2^3)) for FMC-ASPE-Li (CR2032 coin cell, 70°C, 16 mA·g^{-1} for the first two cycles, 80 mA·g^{-1} from the 3rd cycle, 2.7-4.2 V, FMC-ASPE-Li composition: m_{LiTFSI} : m_{PEO} : $m_{21\text{-}\beta\text{-CD-}g\text{-PTFEMA}}$ = 0.9 : 2 : 1, $M_{n,\ GPC\ (21\text{-}\beta\text{-CD-}g\text{-PTFEMA})}$ = 800k, M_{PEO} = 600k).

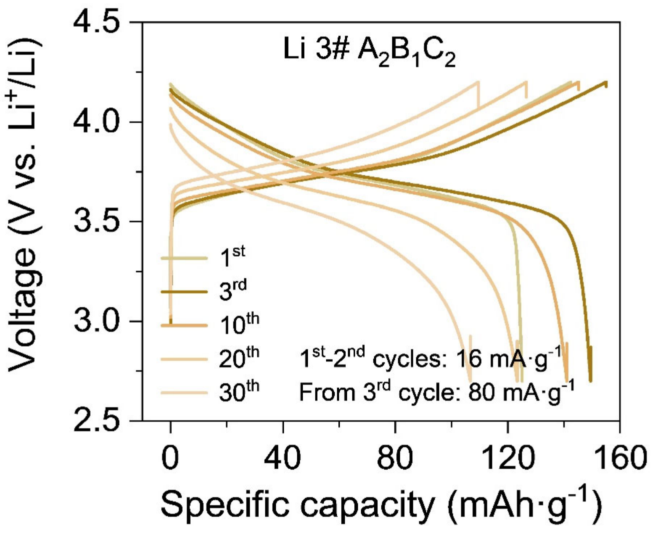

Supplementary Figure 15 Charge/discharge voltage profiles of the experiment 3 selected by the orthogonal experimental design (L_4 (2^3)) for FMC-ASPE-Li (CR2032 coin cell, 70°C, 16 mA·g^{-1} for the first two cycles, 80 mA·g^{-1} from the 3rd cycle, 2.7-4.2 V, FMC-ASPE-Li composition: m_{LiTFSI} : m_{PEO} : $m_{21\text{-}\beta\text{-CD-}g\text{-PTFEMA}}$ = 1.5 : 4 : 1, $M_{n,\ GPC\ (21\text{-}\beta\text{-CD-}g\text{-PTFEMA})}$ = 900k, M_{PEO} = 600k).

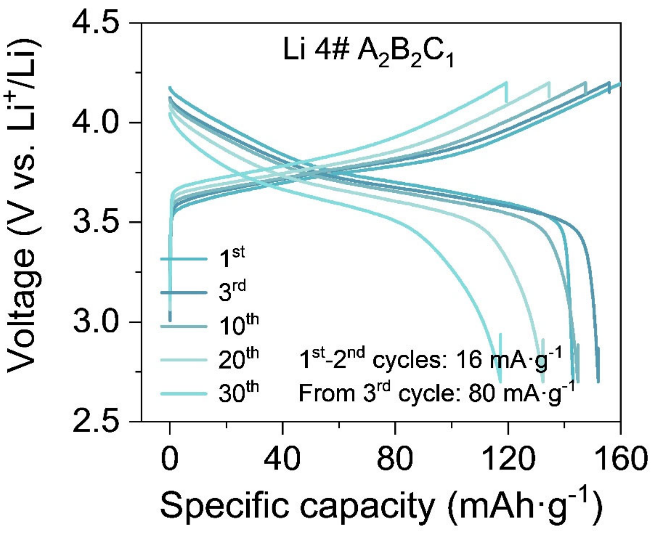

Supplementary Figure 16 Charge/discharge voltage profiles of the experiment 4 selected by the orthogonal experimental design (L_4 (2^3)) for FMC-ASPE-Li (CR2032 coin cell, 70°C, 16 mA·g^{-1} for the first two cycles, 80 mA·g^{-1} from the 3rd cycle, 2.7-4.2 V, FMC-ASPE-Li composition: m_{LiTFSI} : m_{PEO} : $m_{21\text{-}\beta\text{-CD-}g\text{-PTFEMA}}$ = 0.6 : 2 : 1, $M_{n,\ GPC\ (21\text{-}\beta\text{-CD-}g\text{-PTFEMA})}$ = 900k, M_{PEO} = 600k).

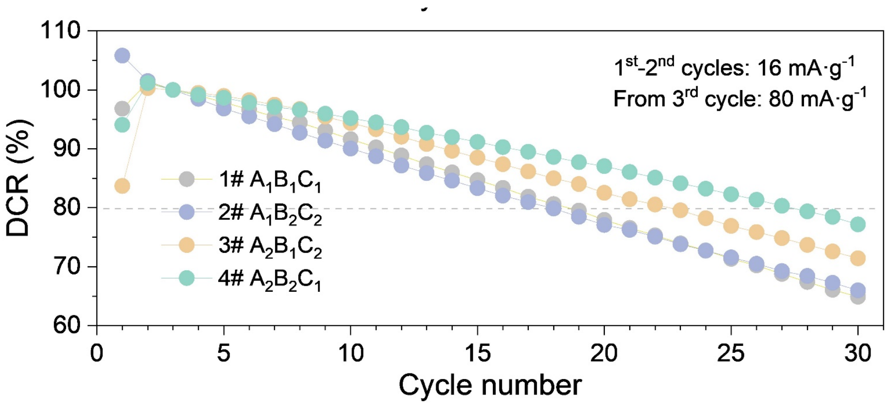

Supplementary Figure 17 Discharge capacity retention (DCR) comparison of the 4 experiments selected by the orthogonal experimental design (L_4 (2^3)) for FMC-ASPE-Li (CR2032 coin cell, 70°C, 16 $mA\cdot g^{-1}$ for the first two cycles, 80 $mA\cdot g^{-1}$ from the 3rd cycle, 2.7-4.2 V).

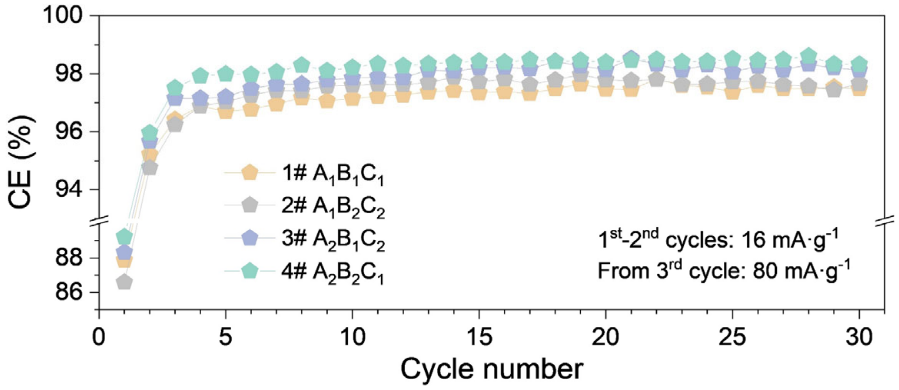

Supplementary Figure 18 Coulombic efficiency (CE) comparison of the 4 experiments selected by the orthogonal experimental design (L_4 (2^3)) for FMC-ASPE-Li (CR2032 coin cell, 70°C, 16 $mA\cdot g^{-1}$ for the first two cycles, 80 $mA\cdot g^{-1}$ from the 3rd cycle, 2.7-4.2 V).

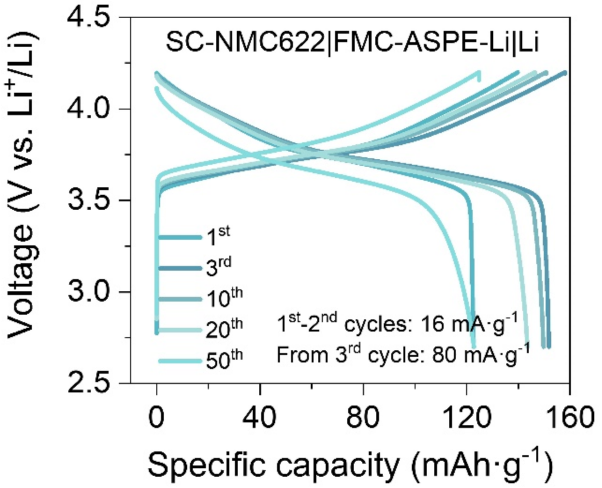

Supplementary Figure 19 Charge/discharge voltage profiles of SC-NMC622|FMC-ASPE-Li|Li cell with the optimal FMC-ASPE-Li selected by the orthogonal experimental design (L_4 (2^3)) (CR2032 coin cell, 70°C, 16 mA·g^{-1} for the first two cycles, 80 mA·g^{-1} from the 3rd cycle, 2.7-4.2 V, FMC-ASPE-Li composition: m_{LiTFSI} : m_{PEO} : $m_{21\text{-}\beta\text{-CD-}g\text{-PTFEMA}}$ = 0.6 : 2 : 1, $M_{n,\ GPC\ (21\text{-}\beta\text{-CD-}g\text{-PTFEMA})}$ = 900k, M_{PEO} = 600k).

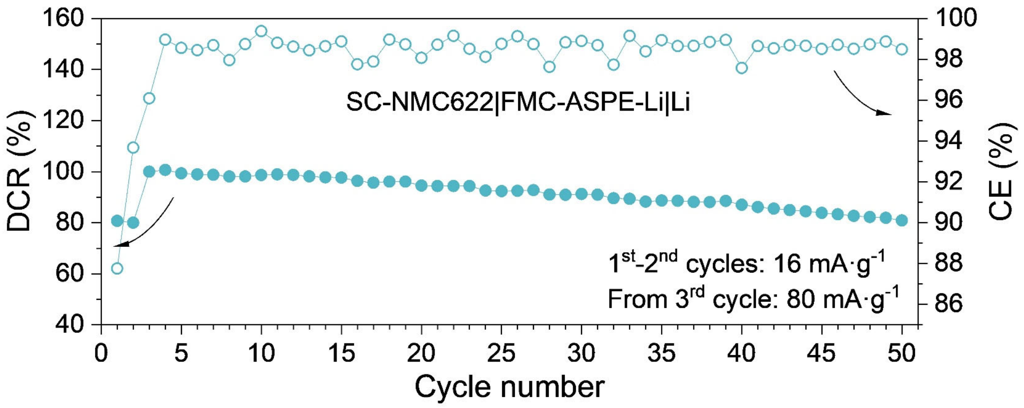

Supplementary Figure 20 Coulombic efficiency (CE) and discharge capacity retention (DCR) of SC-NMC622|FMC-ASPE-Li|Li cell with the optimal FMC-ASPE-Li selected by the orthogonal experimental design (L_4 (2^3)) (CR2032 coin cell, 70°C, 16 mA·g^{-1} for the first two cycles, 80 mA·g^{-1} from the 3rd cycle, 2.7-4.2 V, FMC-ASPE-Li composition: m_{LiTFSI} : m_{PEO} : $m_{21\text{-}\beta\text{-CD-}g\text{-PTFEMA}}$ = 0.6 : 2 : 1, $M_{n,\ GPC\ (21\text{-}\beta\text{-CD-}g\text{-PTFEMA})}$ = 900k, M_{PEO} = 600k).

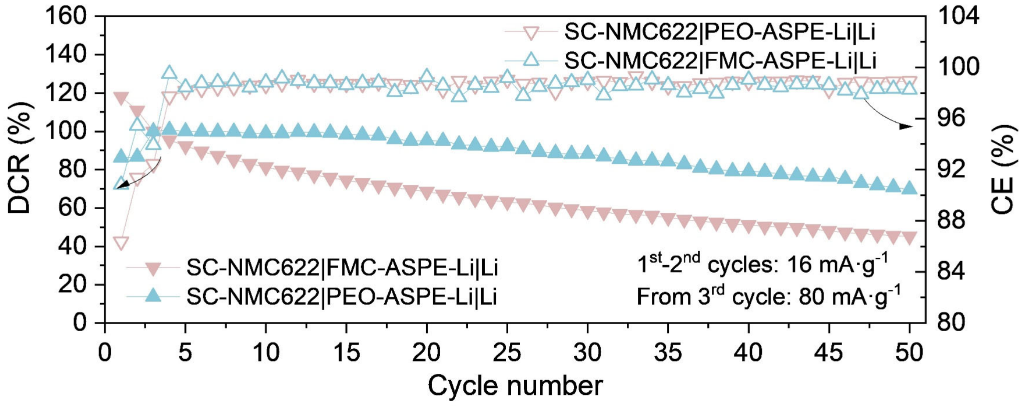

Supplementary Figure 21 Comparing coulombic efficiency (CE) and discharge capacity retention (DCR) of SC-NMC622|FMC-ASPE-Li|Li cell and SC-NMC622|PEO-ASPE-Li|Li cell with SC-NMC622 loading mass of 3-4 mg·cm^{-2}, (CR2032 coin cell, 70°C, 16 mA·g^{-1} for the first two cycles, 80 mA·g^{-1} from the 3rd cycle, 2.7-4.2 V, FMC-ASPE-Li composition: m_{LiTFSI} : m_{PEO} : $m_{21\text{-}\beta\text{-CD-}g\text{-PTFEMA}}$ = 0.6 : 2 : 1, PEO-ASPE-Li composition: m_{LiTFSI} : m_{PEO} = 1 : 5, $M_{n,\,GPC\,(21\text{-}\beta\text{-CD-}g\text{-PTFEMA})}$ = 900k, M_{PEO} = 600k).

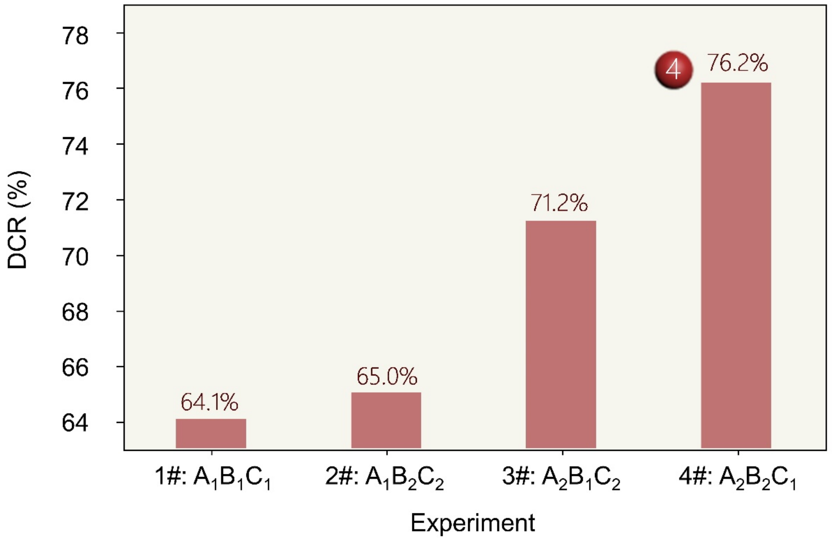

Supplementary Figure 22 The comparison of discharge capacity retention (DCR) of the 4 experiments selected by the orthogonal experimental design (L_4 (2^3)) for FMC-ASPE-Li (CR2032 coin cell, 70°C, 16 mA·g^{-1} for the first two cycles, 80 mA·g^{-1} from the 3rd cycle, 2.7-4.2 V).

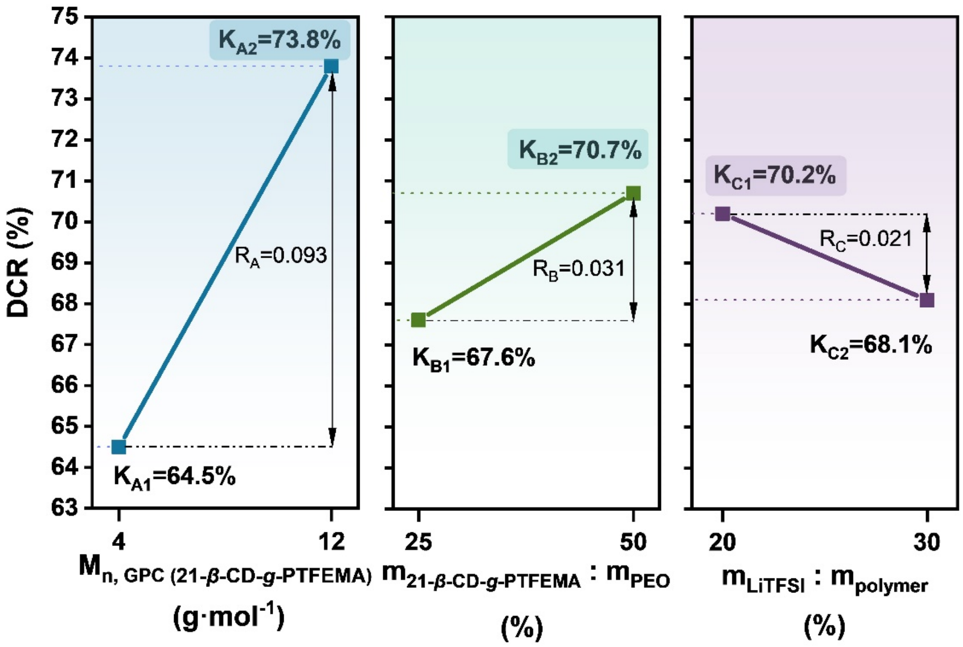

Supplementary Figure 23 The orthogonal test result analysis of each factor. K_i^F is the mean value of discharge capacity retention (DCR, %) of level i of factor F, and the R_F is the sum of the square of deviation, which reflects the influence of factor F on the DCR. The best composition of the FMC-ASPE-Li could be confirmed by combining the levels with the highest K value of each factor ($A_2B_2C_1$).

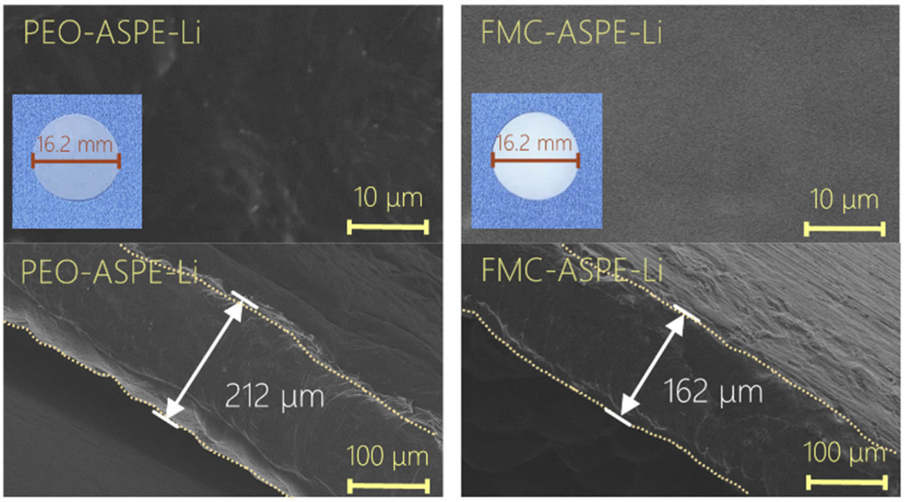

Supplementary Figure 24 Top and cross-section SEM images and optical photographs of the PEO-ASPE-Li and FMC-ASPE-Li membranes.

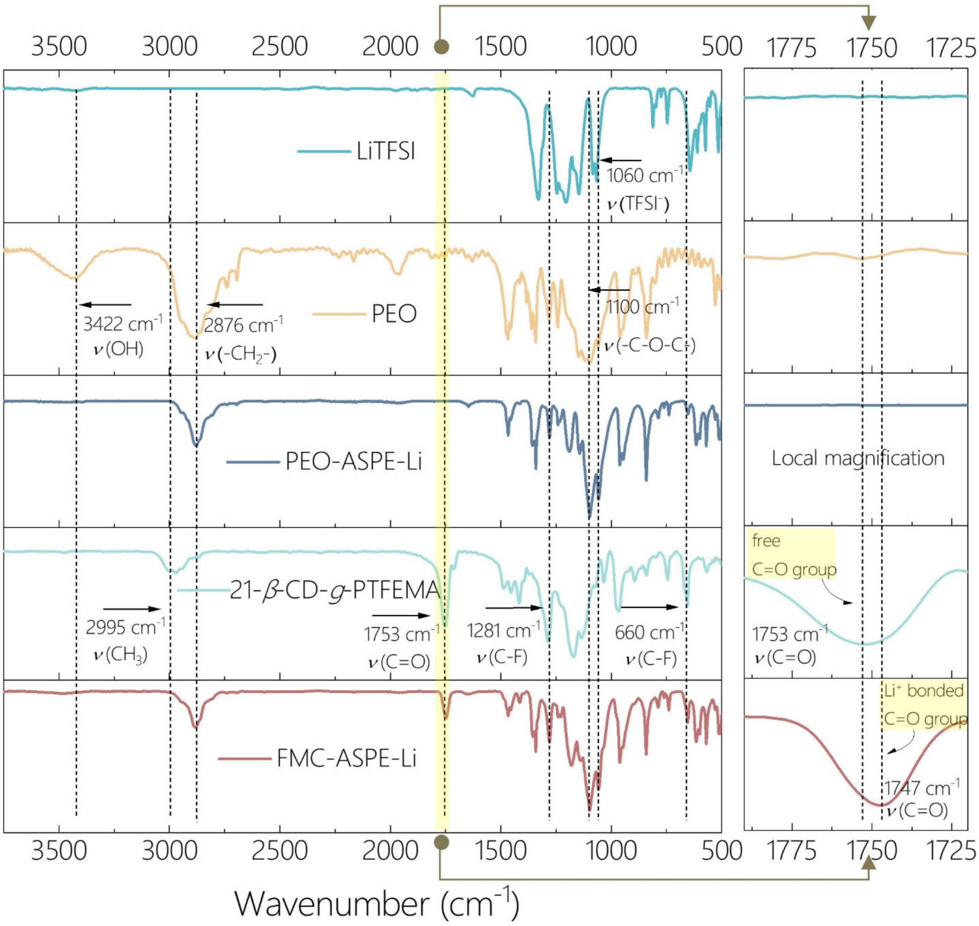

Supplementary Figure 25 FTIR spectra of LiTFSI, PEO, PEO-ASPE-Li, 21-*β*-CD-*g*-PTFEMA, and FMC-ASPE-Li. The testing temperature was 25 °C.

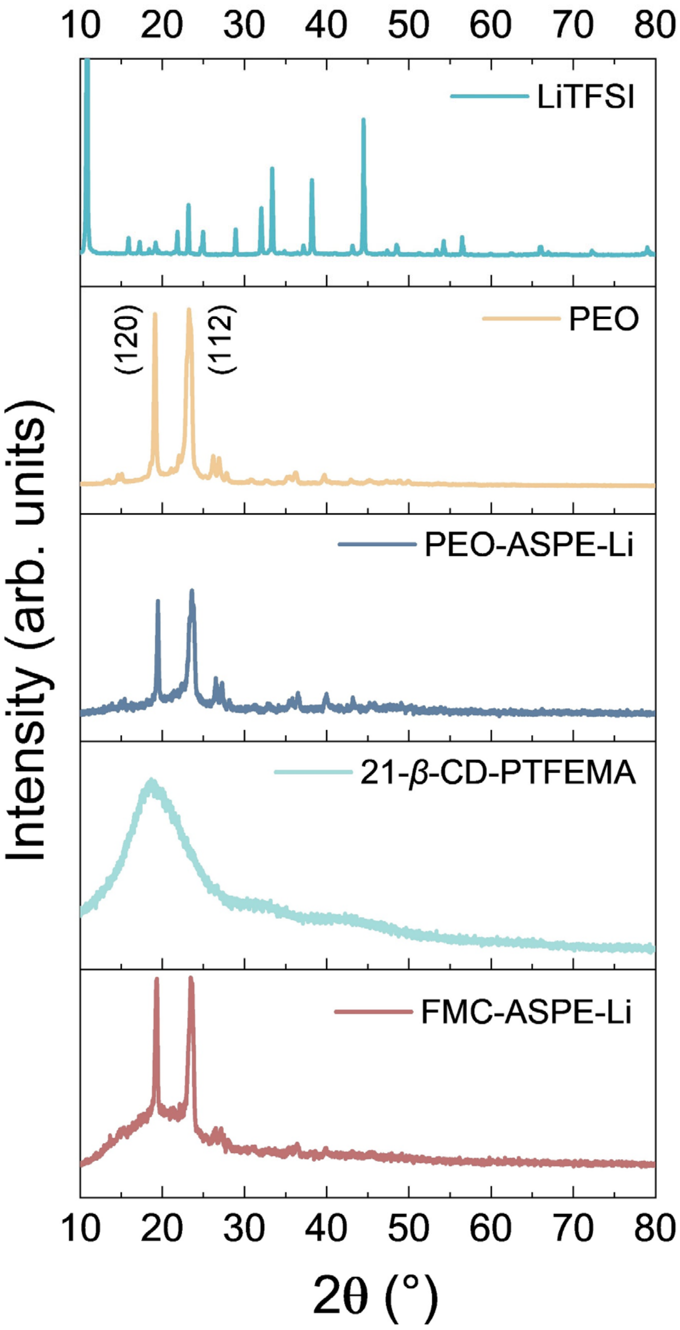

Supplementary Figure 26 **XRD patterns of LiTFSI, PEO, PEO-ASPE-Li, 21-*β*-CD-*g*-PTFEMA, and FMC-ASPE-Li.**

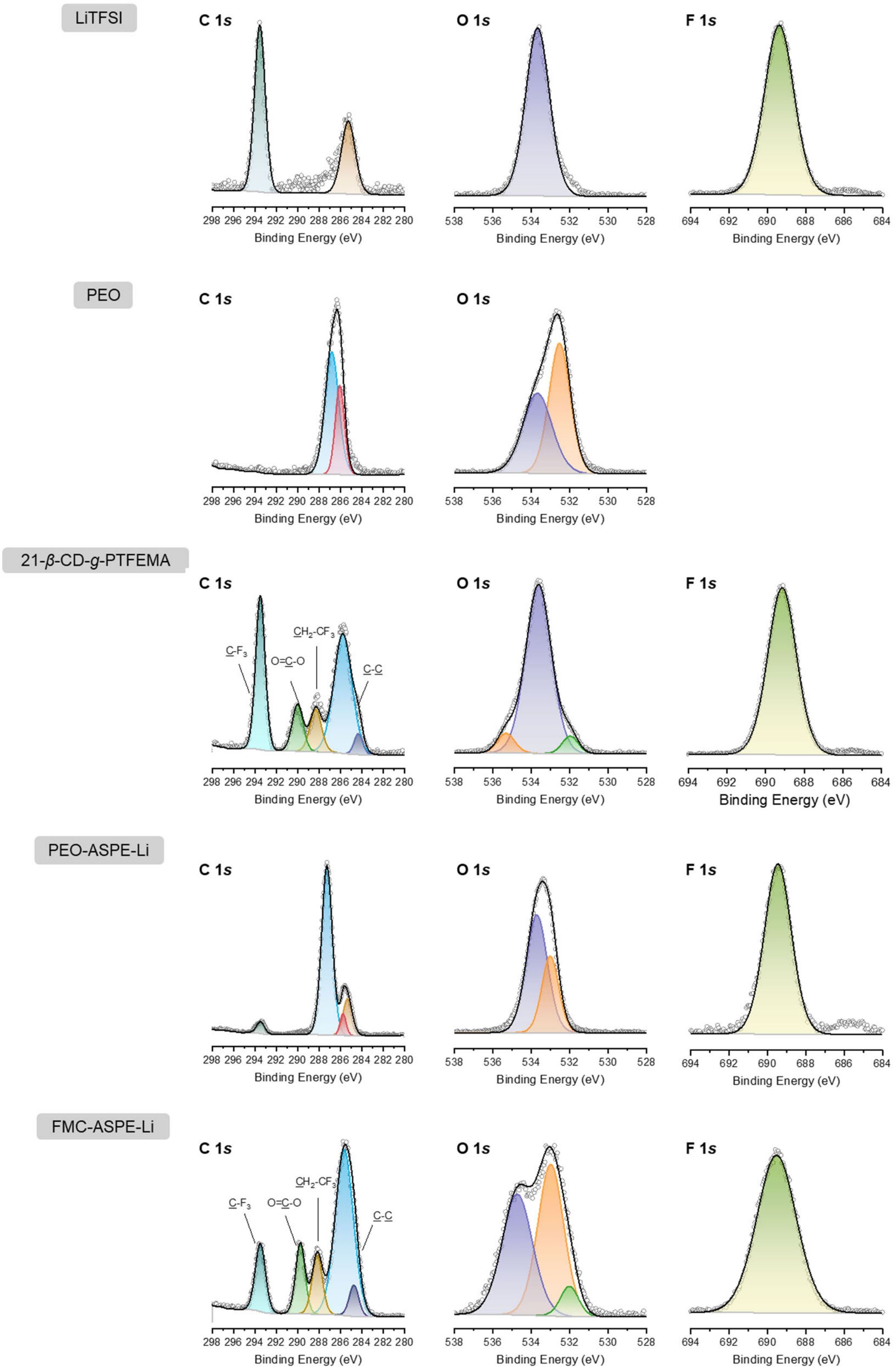

Supplementary Figure 27 **The XPS results of LiTFSI, PEO, 21-*β*-CD-*g*-PTFEMA, PEO-ASPE-Li, and FMC-ASPE-Li.**

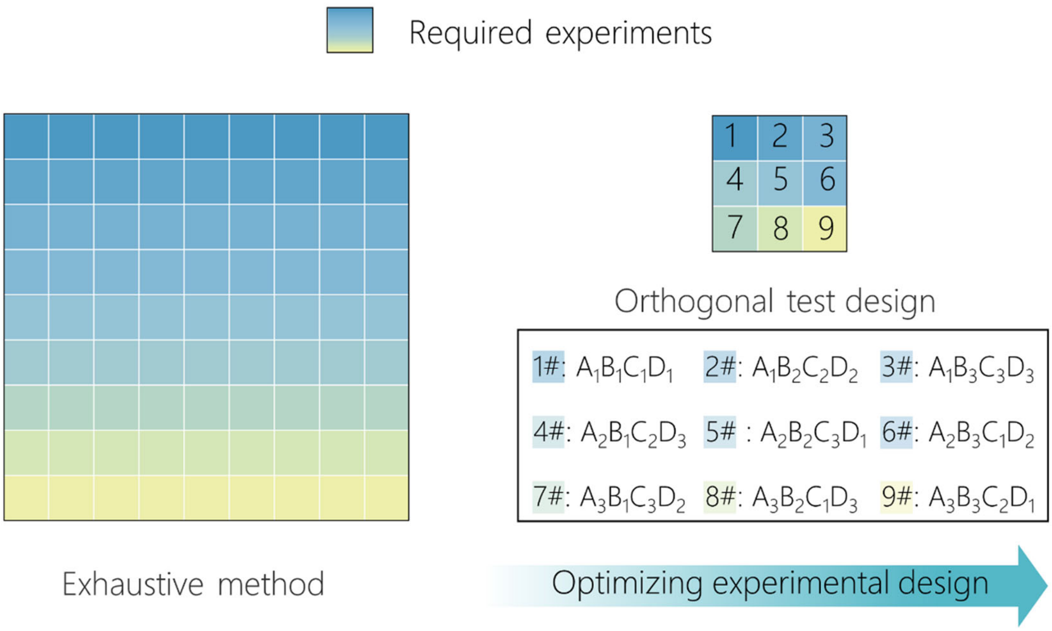

Supplementary Figure 28 **The orthogonal test method used for FMC-ASPE-Na with an L9 (3^4) orthogonal array.**

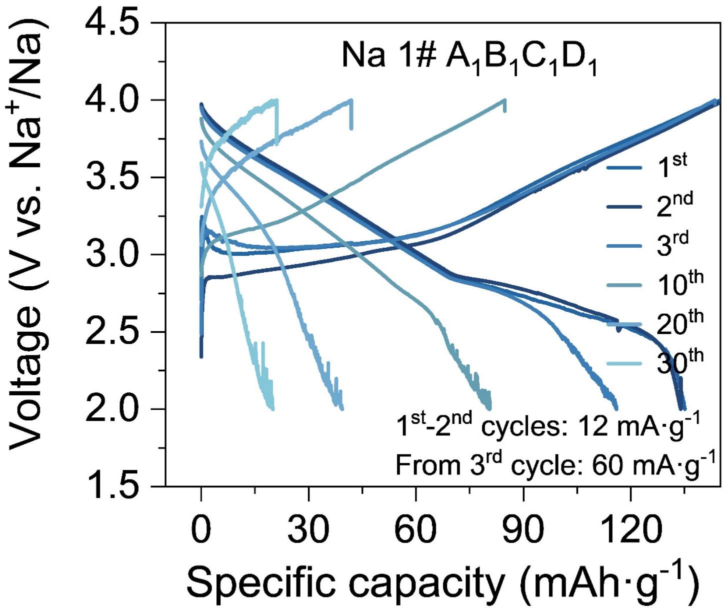

Supplementary Figure 29 **Charge/discharge voltage profiles of the experiment 1 selected by the orthogonal experimental design (L_9 (3^4)) for FMC-ASPE-Na (CR2032 coin cell, 80°C, 12 mA·g^{-1} for the first two cycles, 60 mA·g^{-1} from the 3rd cycle, 2.0-4.0 V, FMC-ASPE-Na composition: m_{NaPF6} : m_{PEO} : $m_{21\text{-}\beta\text{-CD-}g\text{-PTFEMA}}$ = 0.3 : 2 : 1, $M_{n,\ GPC\ (21\text{-}\beta\text{-CD-}g\text{-PTFEMA})}$ = 100k, M_{PEO} = 600k).**

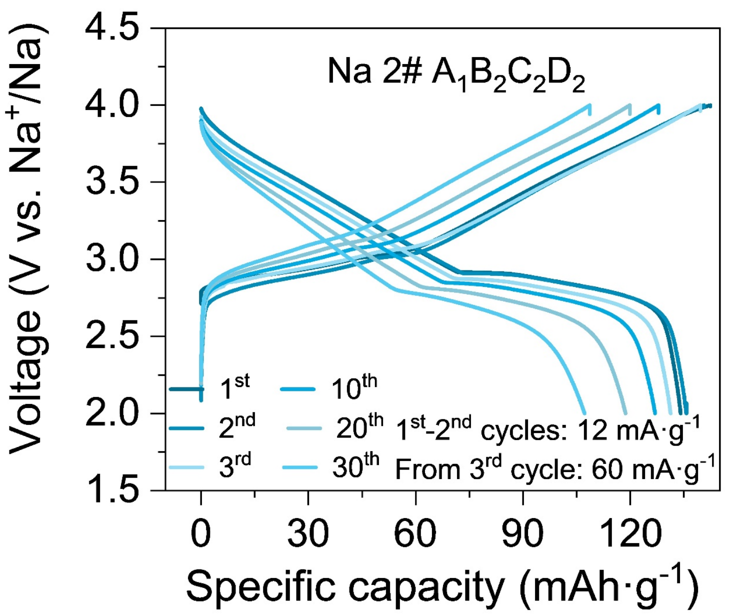

Supplementary Figure 30 **Charge/discharge voltage profiles of the experiment 2 selected by the orthogonal experimental design (L_9 (3^4)) for FMC-ASPE-Na (CR2032 coin cell, 80°C, 12 mA·g^{-1} for the first two cycles, 60 mA·g^{-1} from the 3rd cycle, 2.0-4.0 V, FMC-ASPE-Na composition: m_{NaPF6} : m_{PEO} : $m_{21\text{-}\beta\text{-CD-}g\text{-PTFEMA}}$ = 0.6 : 3 : 1, $M_{n, GPC\ (21\text{-}\beta\text{-CD-}g\text{-PTFEMA})}$ = 800k, M_{PEO} = 600k).**

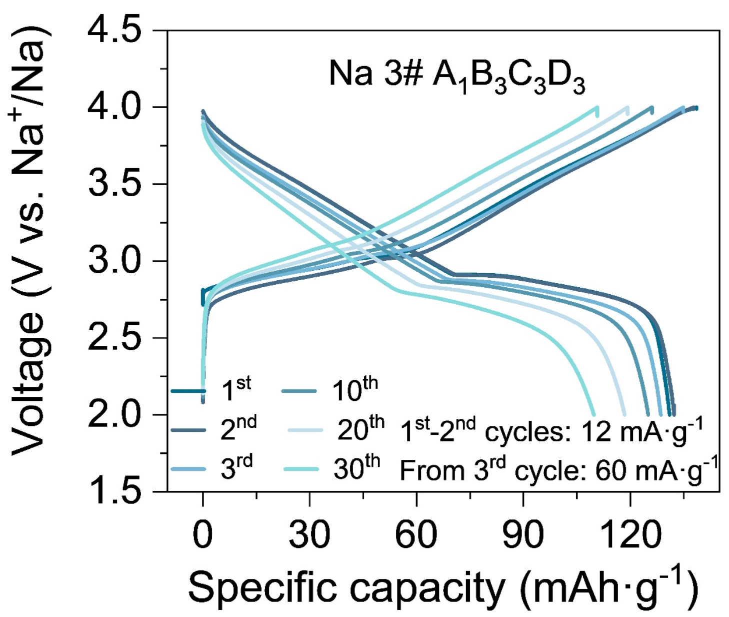

Supplementary Figure 31 **Charge/discharge voltage profiles of the experiment 3 selected by the orthogonal experimental design (L_9 (3^4)) for FMC-ASPE-Na (CR2032 coin cell, 80°C, 12 mA·g^{-1} for the first two cycles, 60 mA·g^{-1} from the 3rd cycle, 2.0-4.0 V, FMC-ASPE-Na composition: m_{NaPF6} : m_{PEO} : $m_{21\text{-}\beta\text{-CD-}g\text{-PTFEMA}}$ = 1 : 4 : 1, $M_{n, GPC\ (21\text{-}\beta\text{-CD-}g\text{-PTFEMA})}$ = 900k, M_{PEO} = 600k).**

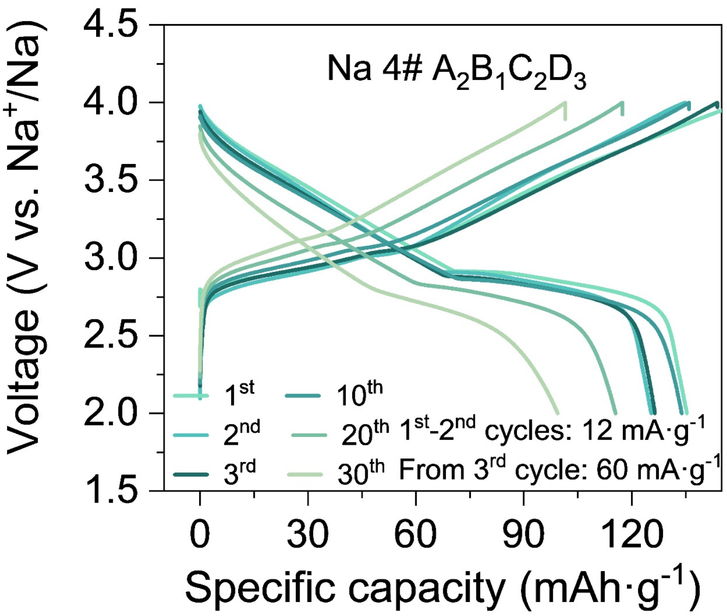

Supplementary Figure 32 Charge/discharge voltage profiles of the experiment 4 selected by the orthogonal experimental design (L_9 (3^4)) for FMC-ASPE-Na (CR2032 coin cell, 80°C, 12 mA·g^{-1} for the first two cycles, 60 mA·g^{-1} from the 3rd cycle, 2.0-4.0 V, FMC-ASPE-Na composition: m_{NaClO4} : m_{PEO} : $m_{21\text{-}\beta\text{-CD-}g\text{-PTFEMA}}$ = 0.4 : 3 : 1, $M_{n,\ GPC\ (21\text{-}\beta\text{-CD-}g\text{-PTFEMA})}$ = 900k, M_{PEO} = 600k).

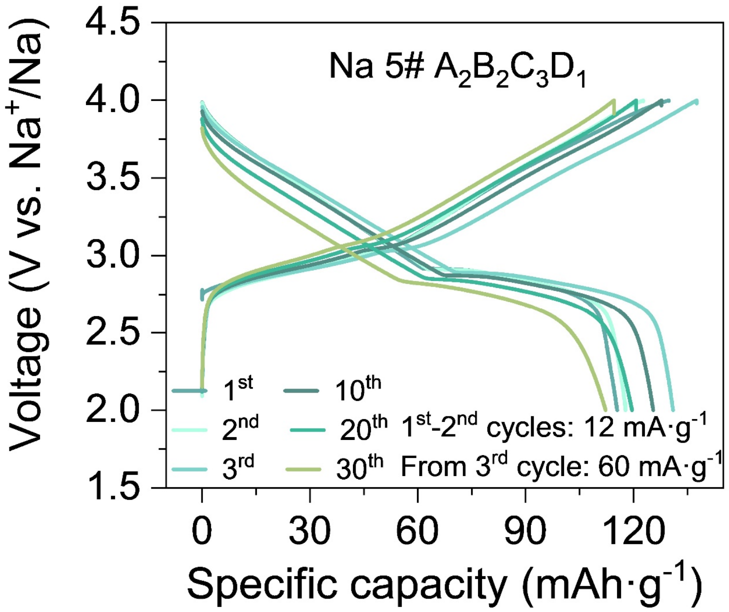

Supplementary Figure 33 Charge/discharge voltage profiles of the experiment 5 selected by the orthogonal experimental design (L_9 (3^4)) for FMC-ASPE-Na (CR2032 coin cell, 80°C, 12 mA·g^{-1} for the first two cycles, 60 mA·g^{-1} from the 3rd cycle, 2.0-4.0 V, FMC-ASPE-Na composition: m_{NaClO4} : m_{PEO} : $m_{21\text{-}\beta\text{-CD-}g\text{-PTFEMA}}$ = 0.75 : 4 : 1, $M_{n,\ GPC\ (21\text{-}\beta\text{-CD-}g\text{-PTFEMA})}$ = 100k, M_{PEO} = 600k).

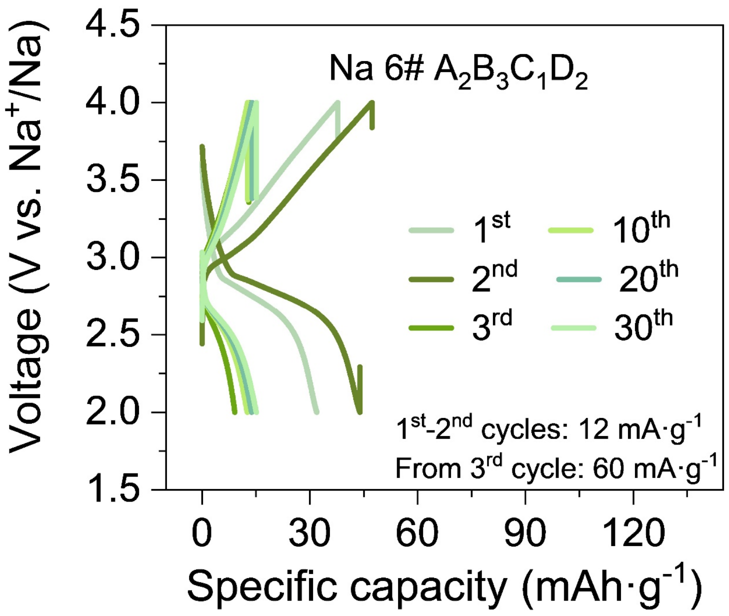

Supplementary Figure 34 Charge/discharge voltage profiles of the experiment 6 selected by the orthogonal experimental design (L_9 (3^4)) for FMC-ASPE-Na (CR2032 coin cell, 80°C, 12 mA·g^{-1} for the first two cycles, 60 mA·g^{-1} from the 3rd cycle, 2.0-4.0 V, FMC-ASPE-Na composition: m_{NaClO4} : m_{PEO} : $m_{21\text{-}\beta\text{-CD-}g\text{-PTFEMA}}$ = 0.6 : 2 : 1, $M_{n,\ GPC\ (21\text{-}\beta\text{-CD-}g\text{-PTFEMA})}$ = 800k, M_{PEO} = 600k).

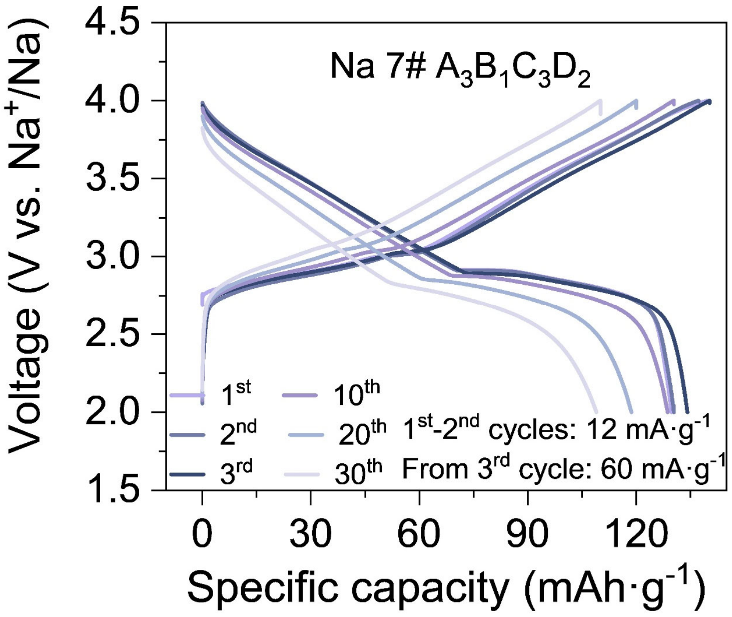

Supplementary Figure 35 Charge/discharge voltage profiles of the experiment 7 selected by the orthogonal experimental design (L_9 (3^4)) for FMC-ASPE-Na (CR2032 coin cell, 80°C, 12 mA·g^{-1} for the first two cycles, 60 mA·g^{-1} from the 3rd cycle, 2.0-4.0 V, FMC-ASPE-Na composition: m_{NaTFSI} : m_{PEO} : $m_{21\text{-}\beta\text{-CD-}g\text{-PTFEMA}}$ = 0.5 : 4 : 1, $M_{n,\ GPC\ (21\text{-}\beta\text{-CD-}g\text{-PTFEMA})}$ = 800k, M_{PEO} = 600k).

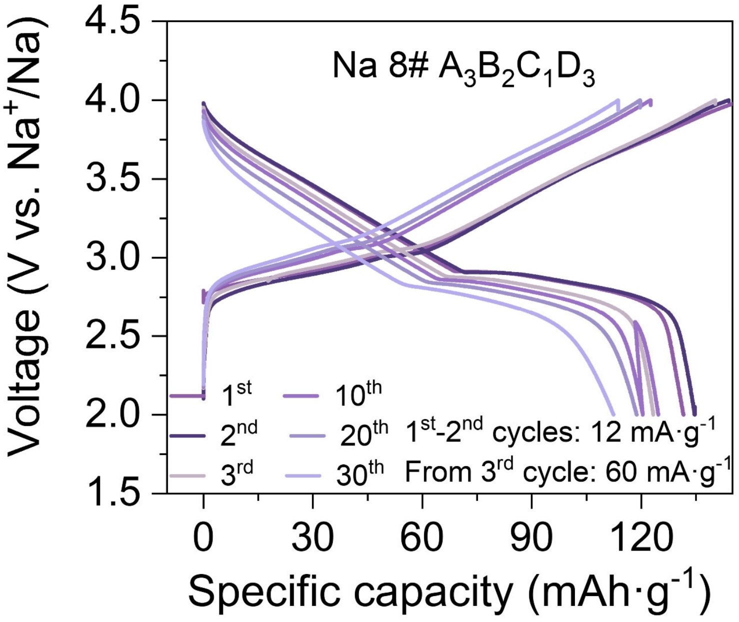

Supplementary Figure 36 **Charge/discharge voltage profiles of the experiment 8 selected by the orthogonal experimental design (L_9 (3^4)) for FMC-ASPE-Na (CR2032 coin cell, 80°C, 12 mA·g^{-1} for the first two cycles, 60 mA·g^{-1} from the 3rd cycle, 2.0-4.0 V, FMC-ASPE-Na composition: m_{NaTFSI} : m_{PEO} : $m_{21\text{-}\beta\text{-CD-}g\text{-PTFEMA}}$ = 0.45 : 2 : 1, $M_{n,\ GPC\ (21\text{-}\beta\text{-CD-}g\text{-PTFEMA})}$ = 900k, M_{PEO} = 600k).**

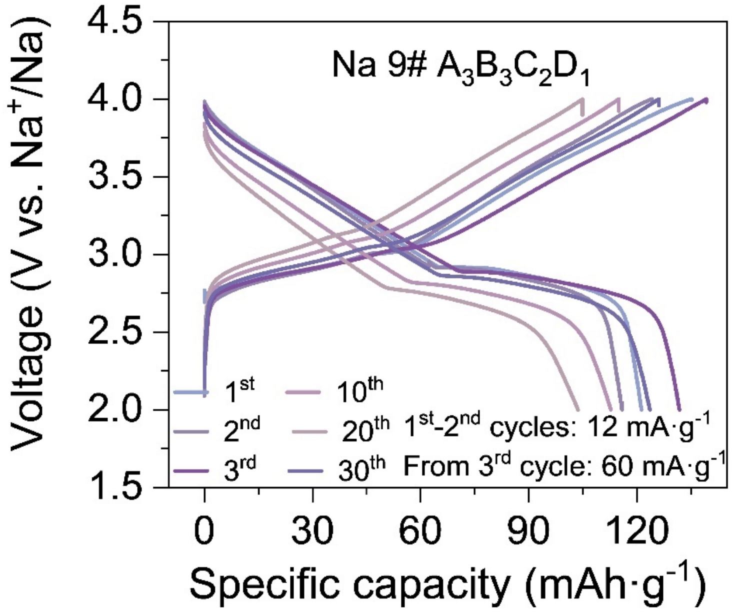

Supplementary Figure 37 **Charge/discharge voltage profiles of the experiment 9 selected by the orthogonal experimental design (L_9 (3^4)) for FMC-ASPE-Na (CR2032 coin cell, 80°C, 12 mA·g^{-1} for the first two cycles, 60 mA·g^{-1} from the 3rd cycle, 2.0-4.0 V, FMC-ASPE-Na composition: m_{NaTFSI} : m_{PEO} : $m_{21\text{-}\beta\text{-CD-}g\text{-PTFEMA}}$ = 0.8 : 3 : 1, $M_{n,\ GPC\ (21\text{-}\beta\text{-CD-}g\text{-PTFEMA})}$ = 900k, M_{PEO} = 600k).**

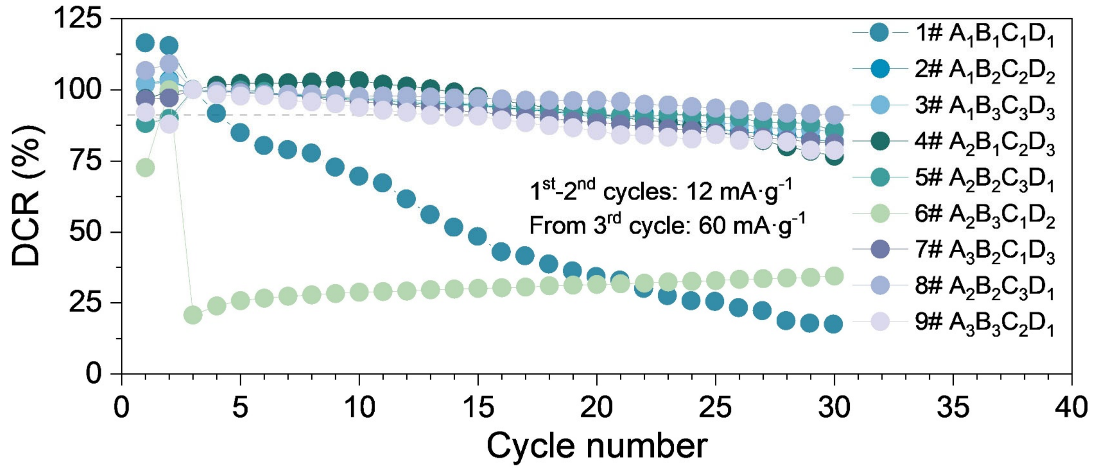

Supplementary Figure 38 Discharge capacity retention (DCR) comparison of the 9 experiments selected by the orthogonal experimental design (L_9 (3^4)) for FMC-ASPE-Na (CR2032 coin cell, 80°C, 12 mA·g^{-1} for the first two cycles, 60 mA·g^{-1} from the 3rd cycle, 2.0-4.0 V).

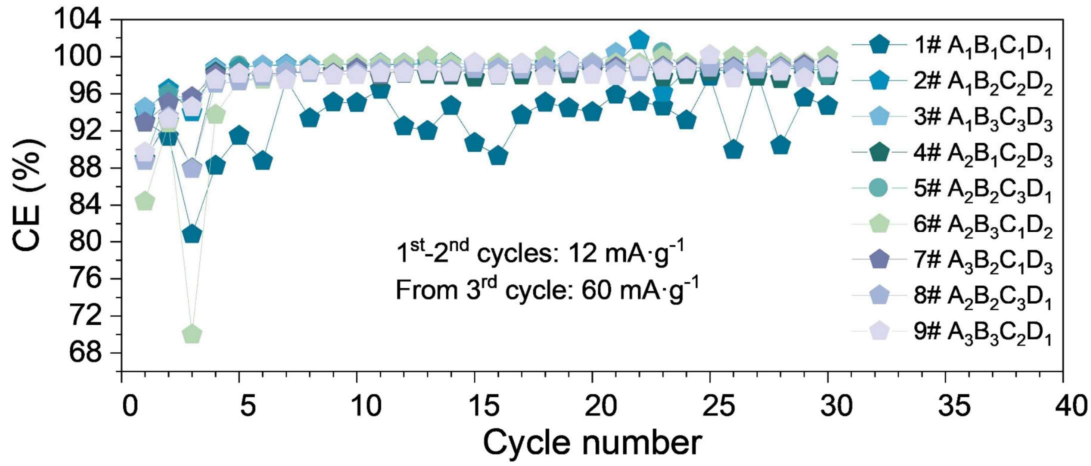

Supplementary Figure 39 Coulombic efficiency (CE) comparison of the 9 experiments selected by the orthogonal experimental design (L_9 (3^4)) for FMC-ASPE-Na (CR2032 coin cell, 80°C, 12 mA·g^{-1} for the first two cycles, 60 mA·g^{-1} from the 3rd cycle, 2.0-4.0 V).

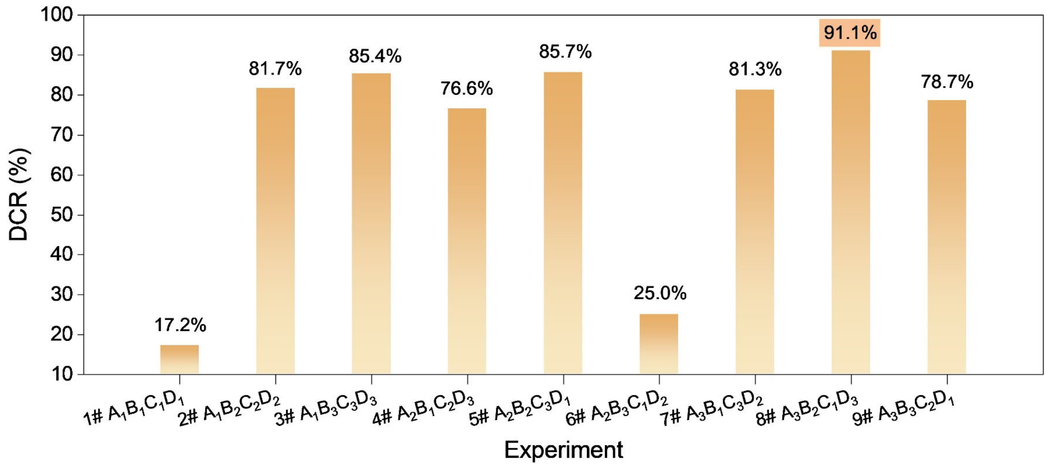

Supplementary Figure 40 The comparison of discharge capacity retention (DCR) of the 9 experiments selected by the orthogonal experimental design (L_9 (3^4)) for FMC-ASPE-Na (CR2032 coin cell, 80°C, 12 mA·g^{-1} for the first two cycles, 60 mA·g^{-1} from the 3rd cycle, 2.0-4.0 V).

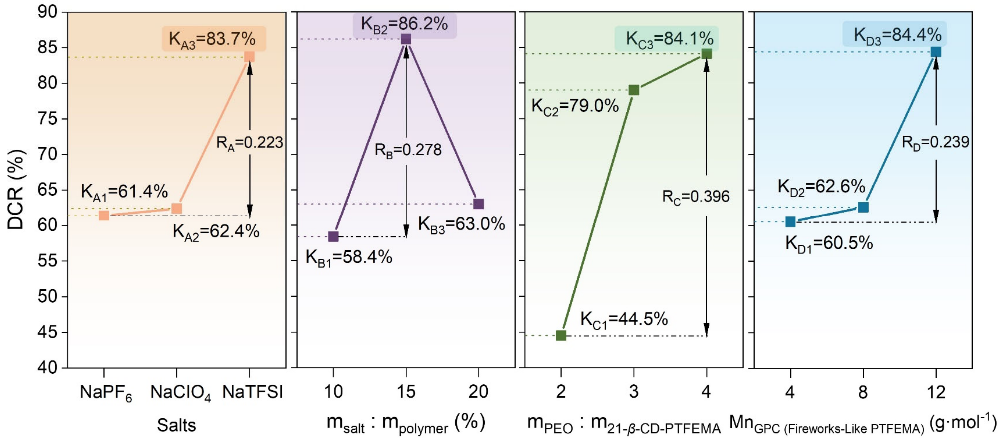

Supplementary Figure 41 Analysis of each factor and level of the orthogonal experimental design (L_9 (3^4)) for FMC-ASPE-Na. K_i^F is the mean value of discharge capacity retention (DCR, %) of level i of factor F, and the R_F is the sum of square of deviation, which reflects the influence of factor F upon the DCR. The best compostion of the FMC-ASPE-Na could be confirmed by combining the levels with highest K value of each factor ($A_3B_2C_3D_3$).

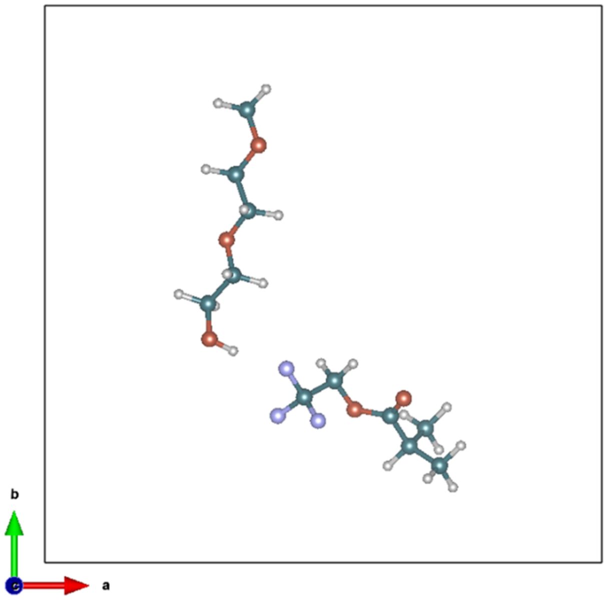

Supplementary Figure 42 Relaxed PEO ($C_5H_{12}O_3$) and PTFEMA ($C_6H_9O_2F_3$) models for DFT calculations to calculate the O-H···F-C bond energy.

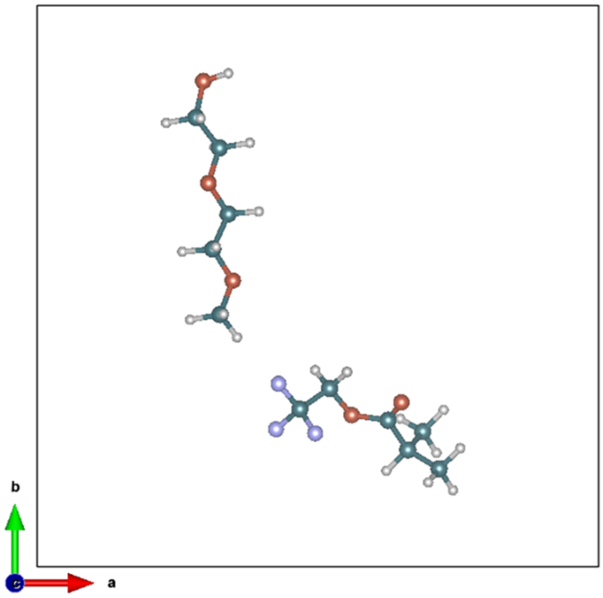

Supplementary Figure 43 Relaxed PEO ($C_5H_{12}O_3$) and PTFEMA ($C_6H_9O_2F_3$) models for DFT calculations to calculate the C-H···F-C bond energy.

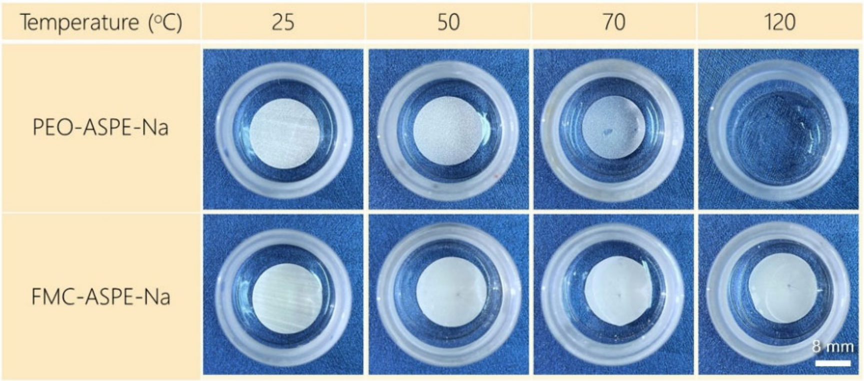

Supplementary Figure 44 Photographic pictures of the thermal dimensional stability of PEO-ASPE-Na and FMC-ASPE-Na after heat treatment for 120 min at different temperatures.

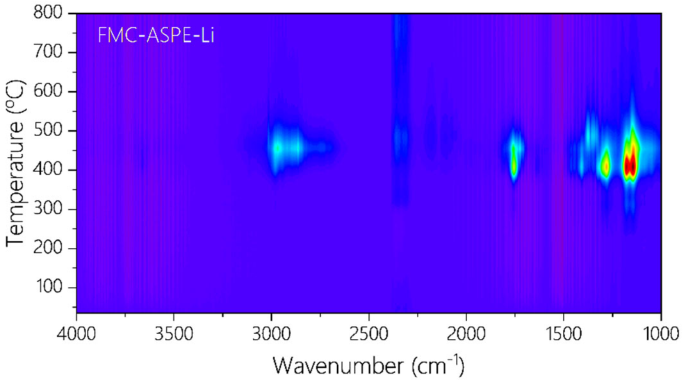

Supplementary Figure 45 TG-FTIR results of the FMC-ASPE-Li membrane. With the addition of 21-β-CD-*g*-PTFEMA, FMC-ASPEs-Li underwent complete decomposition starting at 400°C.

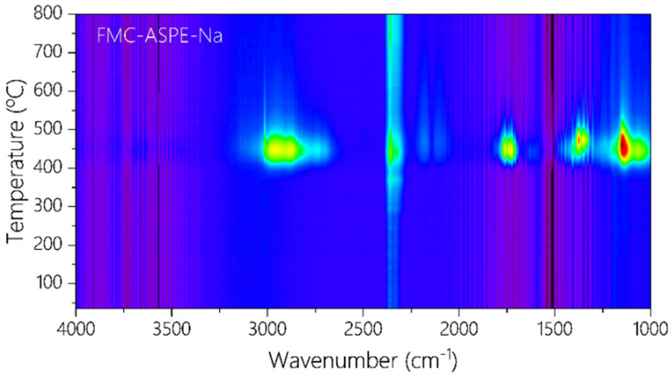

Supplementary Figure 46 TG-FTIR results of the FMC-ASPE-Na membrane. With the addition of 21-β-CD-*g*-PTFEMA, FMC-ASPEs-Na underwent complete decomposition starting at 400°C.

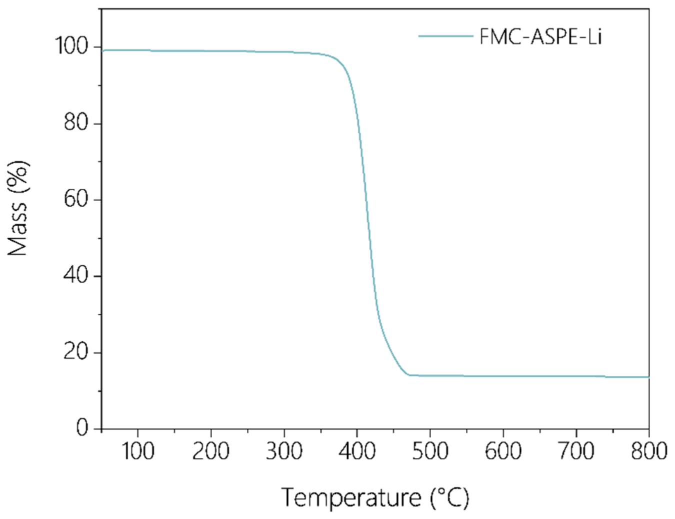

Supplementary Figure 47 TGA curve of the FMC-ASPE-Li.

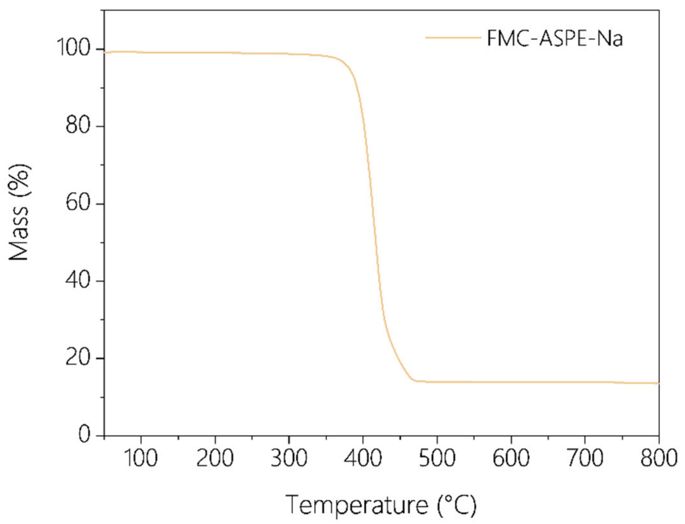

Supplementary Figure 48 TGA curve of the FMC-ASPE-Na.

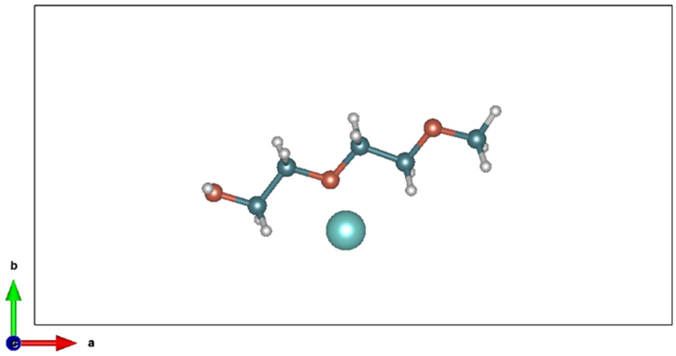

Supplementary Figure 49 **Relaxed Li^+ and PEO ($C_5H_{12}O_3$) models for DFT calculations to calculate the C-O-Li^+ adsorption energy.**

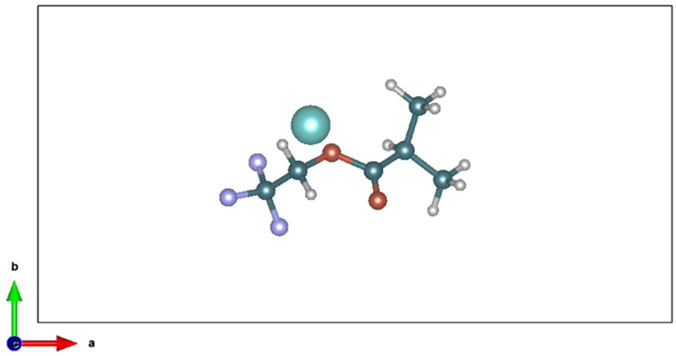

Supplementary Figure 50 **Relaxed Li^+ and PTFEMA ($C_6H_9O_2F_3$) models for DFT calculations to calculate the C-O-Li^+ adsorption energy.**

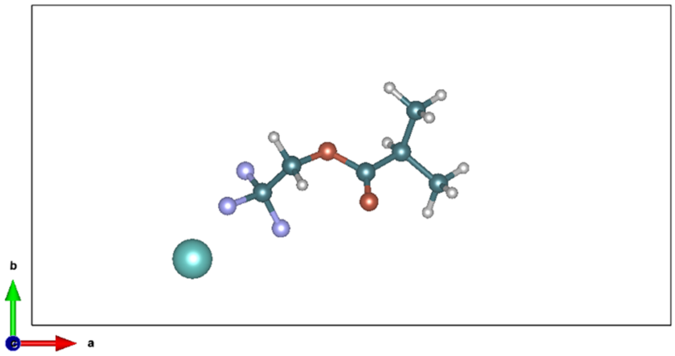

Supplementary Figure 51 Relaxed Li^+ and PTFEMA ($C_6H_9O_2F_3$) models for DFT calculations to calculate the C-F-Li^+ adsorption energy.

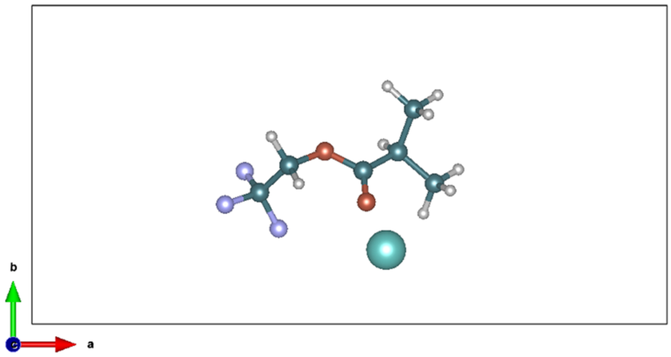

Supplementary Figure 52 Relaxed Li^+ and PTFEMA ($C_6H_9O_2F_3$) models for DFT calculations to calculate the C=O-Li^+ adsorption energy.

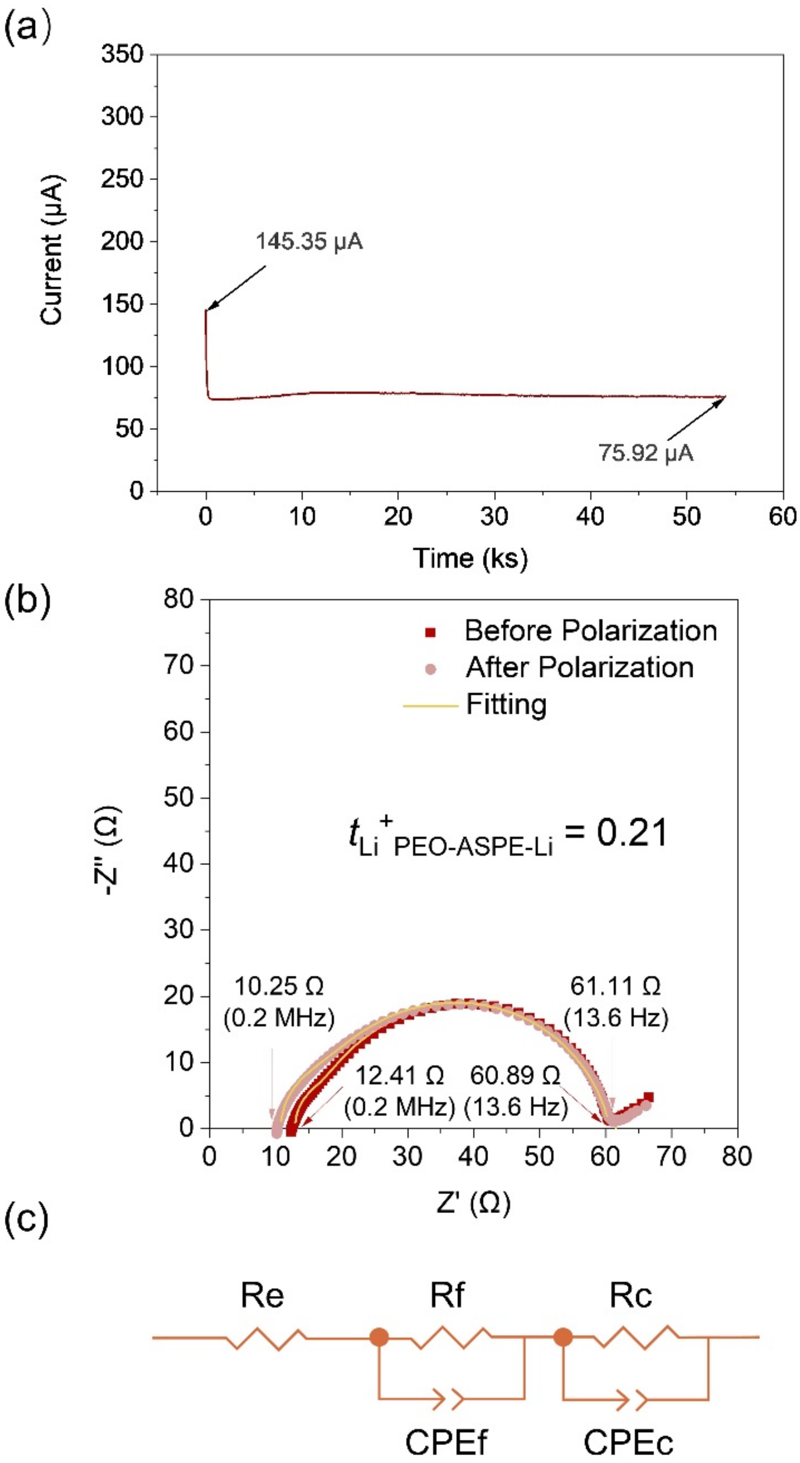

Supplementary Figure 53 Transference number of PEO-ASPE-Li (a) AC impedance spectra before and after polarization, (b) Current variation with the polarization of a Li|PEO-ASPE-Li|Li cell with a potential of 10 mV (c) The equivalent circuit model used for fitting. EIS before and after polarization was acquired at the open circuit potential using a perturbation signal of 10 mV in the frequency range of 4 MHz to 100 mHz at 70°C, with the same procedure as the ionic conductivity test. t_{Li}^{+} was calculated according to the equation (2) in the manuscript methods section.

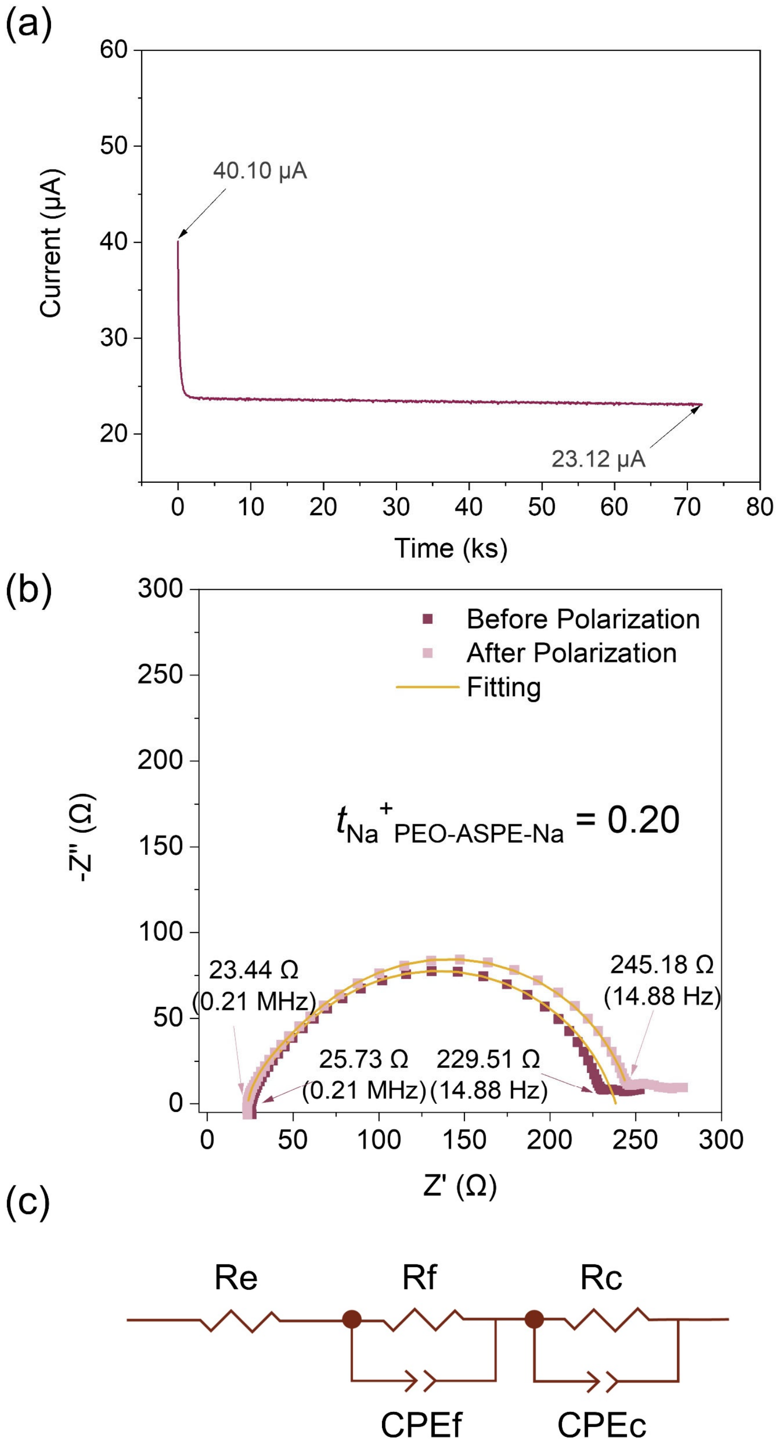

Supplementary Figure 54 Transference number of PEO-ASPE-Na (a) AC impedance spectra before and after polarization, (b) Current variation with the polarization of a Na|PEO-ASPE-Na|Na cell with a potential of 10 mV. (c) The equivalent circuit model used for fitting. EIS before and after polarization was acquired at the open circuit potential using a perturbation signal of 10 mV in the frequency range of 4 MHz to 100 mHz at 80°C, with the same procedure as the ionic conductivity test. t_{Na}^{+} was calculated according to the equation (2) in the manuscript methods section.

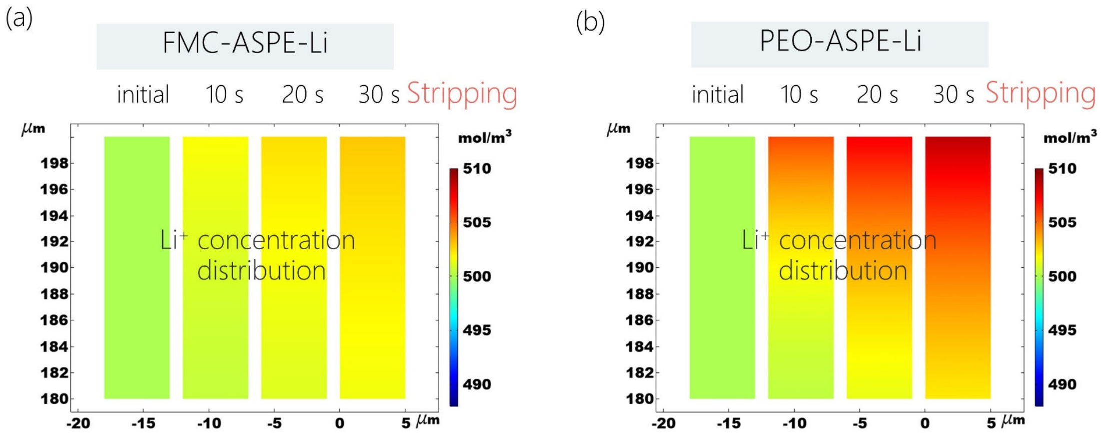

Supplementary Figure 55 Li^+ concentration distribution ascends along with the cathode interface at the initial 10 s, 20 s, and 30 s sequentially during charging derived from the COMSOL simulation results. (a) Li^+ for FMC-ASPE-Li, (b) Li^+ for PEO-ASPE-Li.

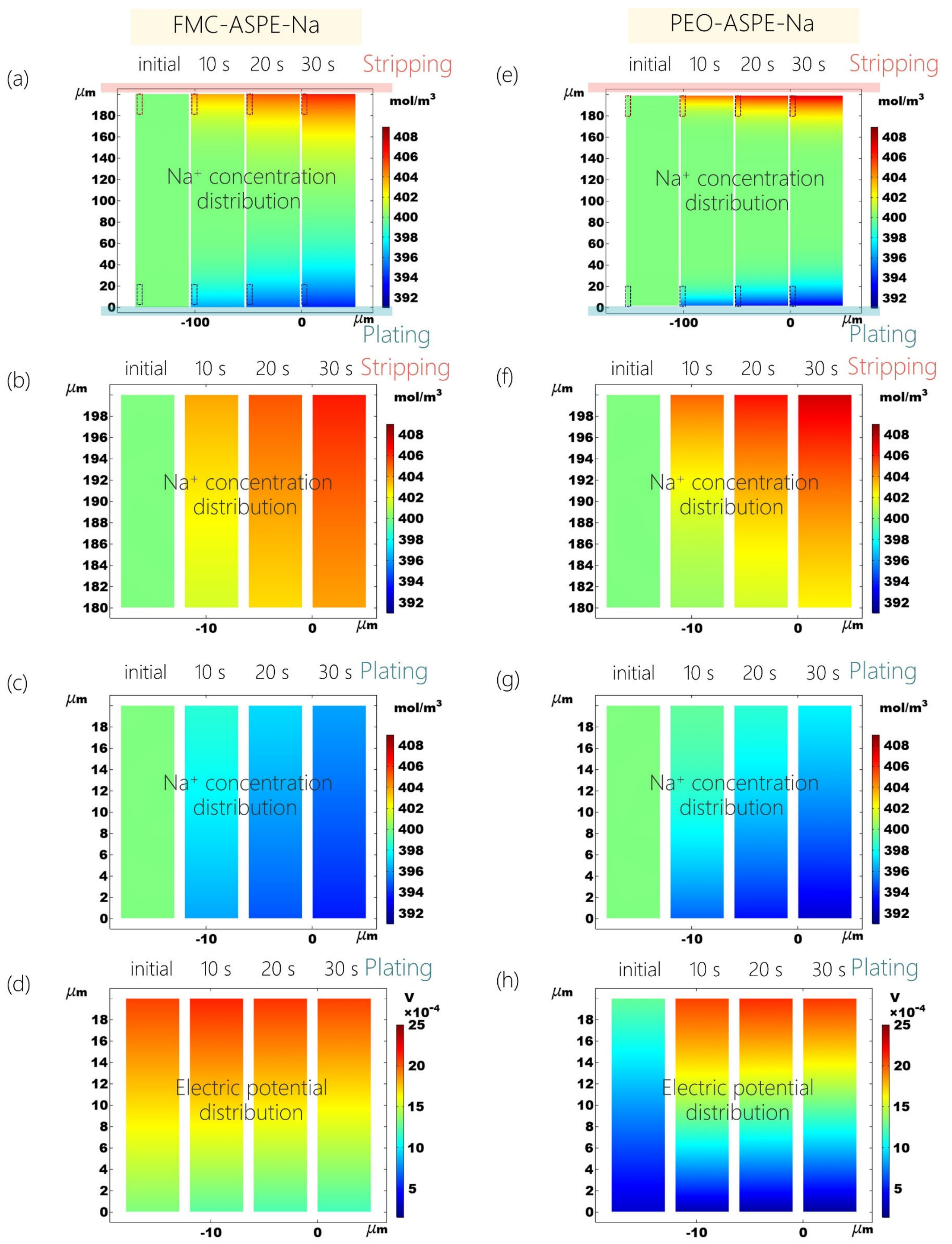

Supplementary Figure 56 Concentration distribution of Na^+ and electric potential in (a-d) FMC-ASPE-Na and (e-h) PEO-ASPE-Na initially and at 10 s, 20 s, and 30 s during Na stripping and Na plating. The Na^+ concentration distribution obtained from FEMS results of the (a) whole, (b) near-region of the Na-stripping side, and (c) near-region of the Na-plating side in the FMC-ASPE-Na membrane and the (e) whole, (f) near-region of the Na-striping side, and (g) near-region of the Na-plating side in the PEO-ASPE-Na membrane. The electric potential distribution near the region of the Na-plating side in the (d) FMC-ASPE-Na and (h) PEO-ASPE-Na membranes.

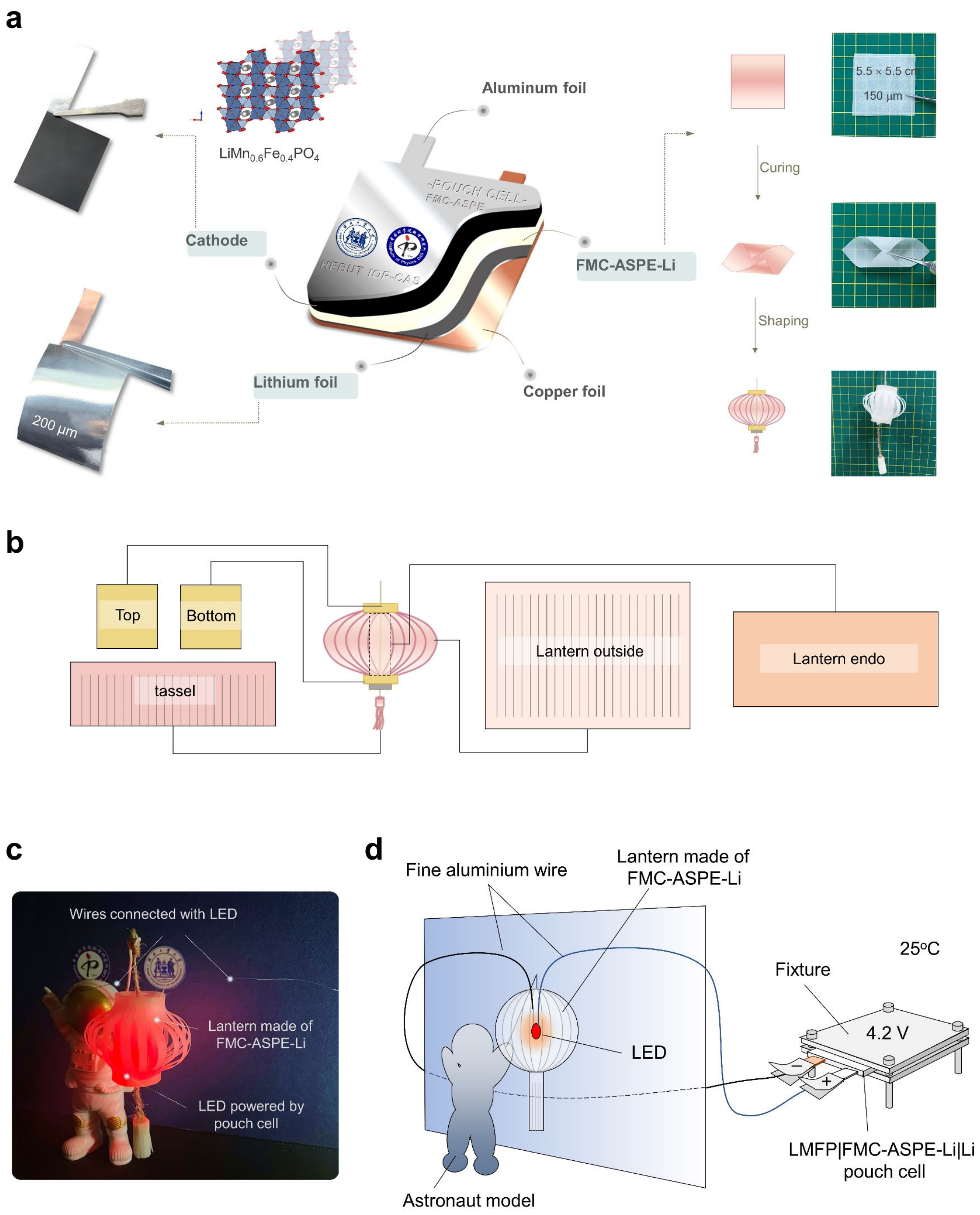

Supplementary Figure 57 a, Schematic diagram of the LMFP|FMC-ASPE-Li|Li pouch cell, optical photographs of the LMFP cathode material, and the lithium metal electrode. b, The exploded view of the lantern. c, The photograph of an astronaut model lifting the lantern (made by FMC-ASPE-Li membrane) with LED powered by LMFP|FMC-ASPE-Li|Li pouch cell. d, The circuit diagram of (c).

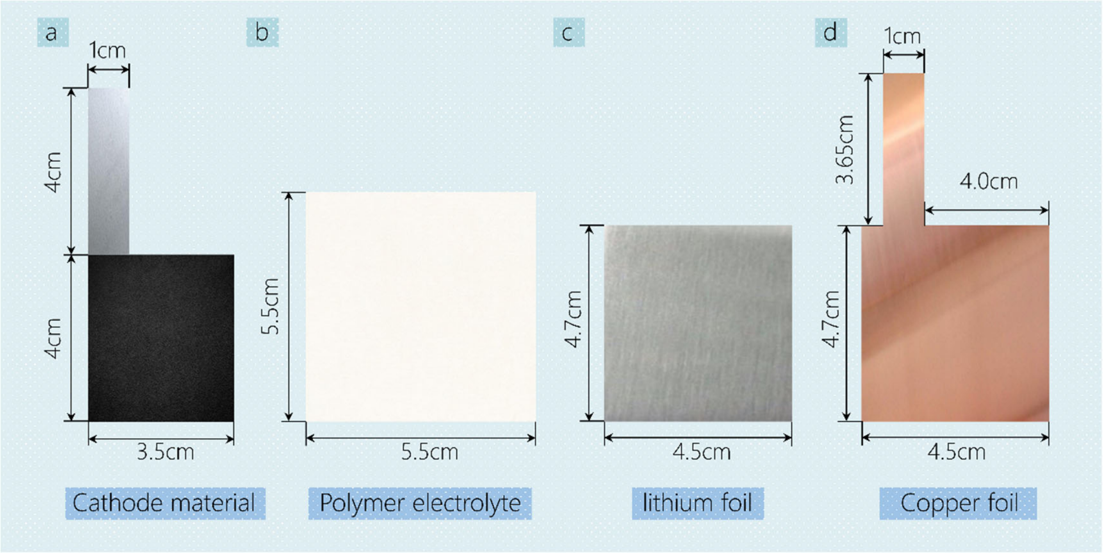

Supplementary Figure 58 The composition of the pouch cell and the size of each part.

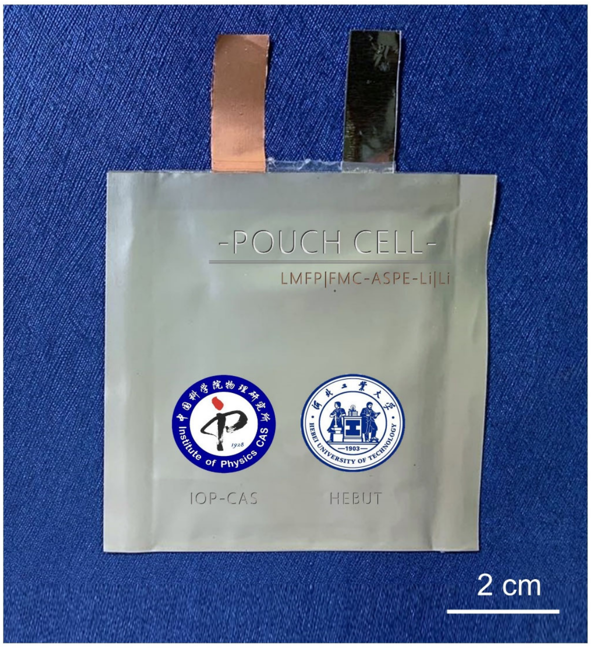

Supplementary Figure 59 Optical photograph of the pouch cell.

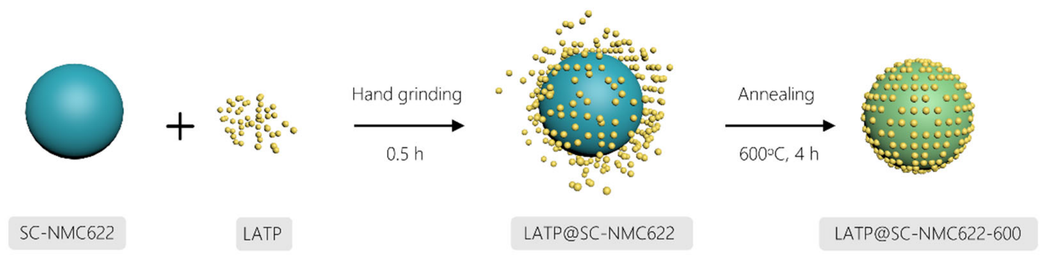

Supplementary Figure 60 **Schematic diagram of the preparation of LATP-coated SC-NMC622.**

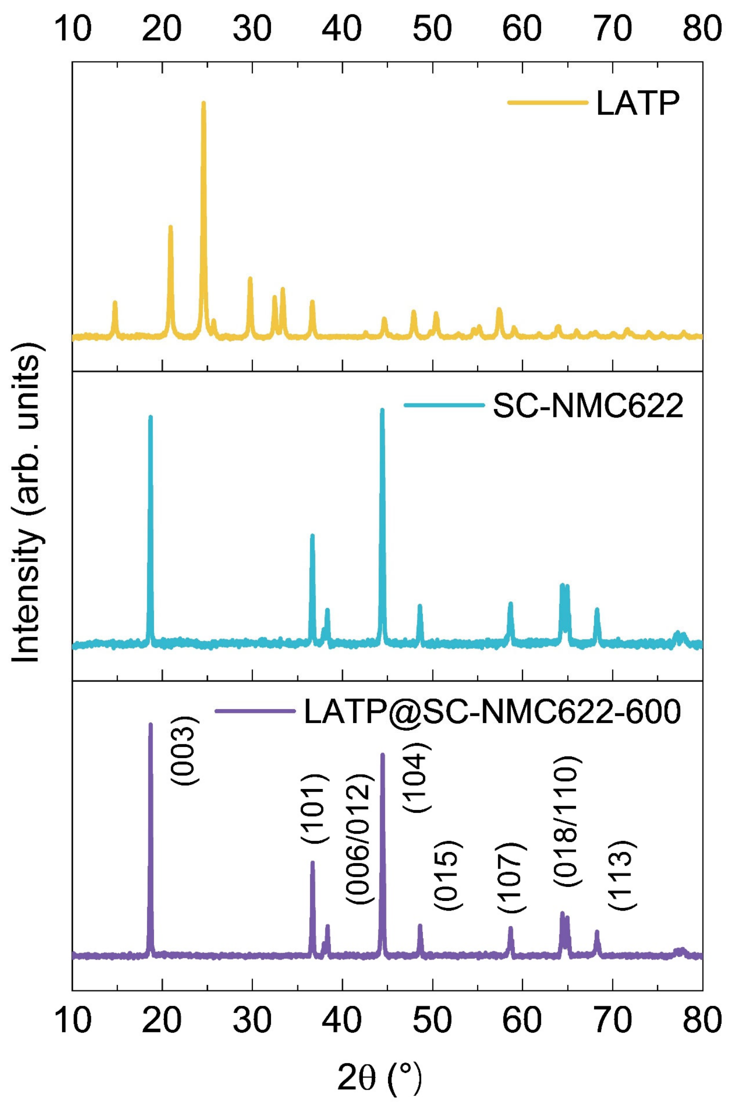

Supplementary Figure 61 **XRD patterns of LATP powder, bare SC-NMC622, and LATP-modified SC-NMC622-600 materials.**

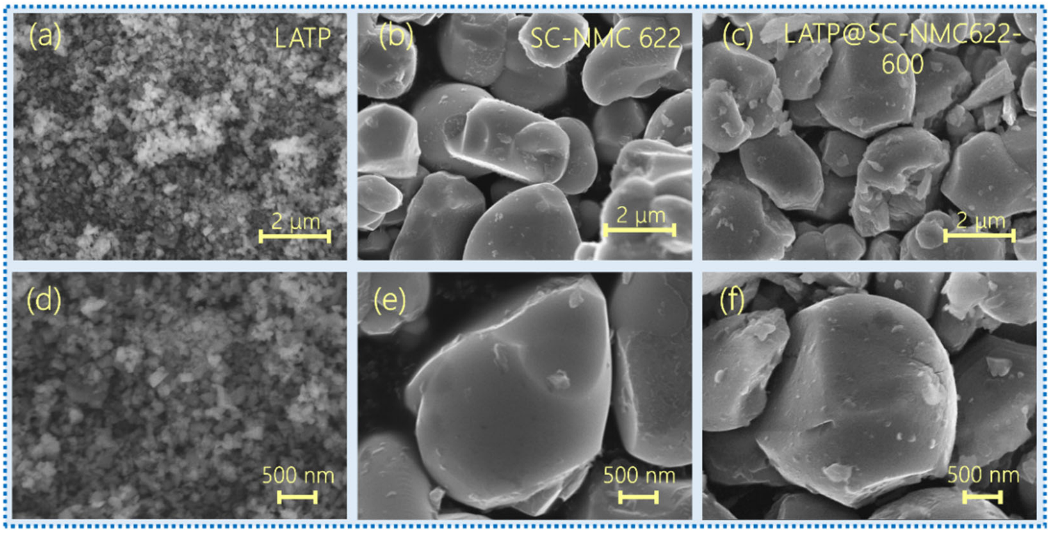

Supplementary Figure 62 **SEM images of (a, d) LATP powder, (b,e) bare SC-NMC622, and (c,f) the LATP@SC-NMC622-600 sample annealed at 600°C.**

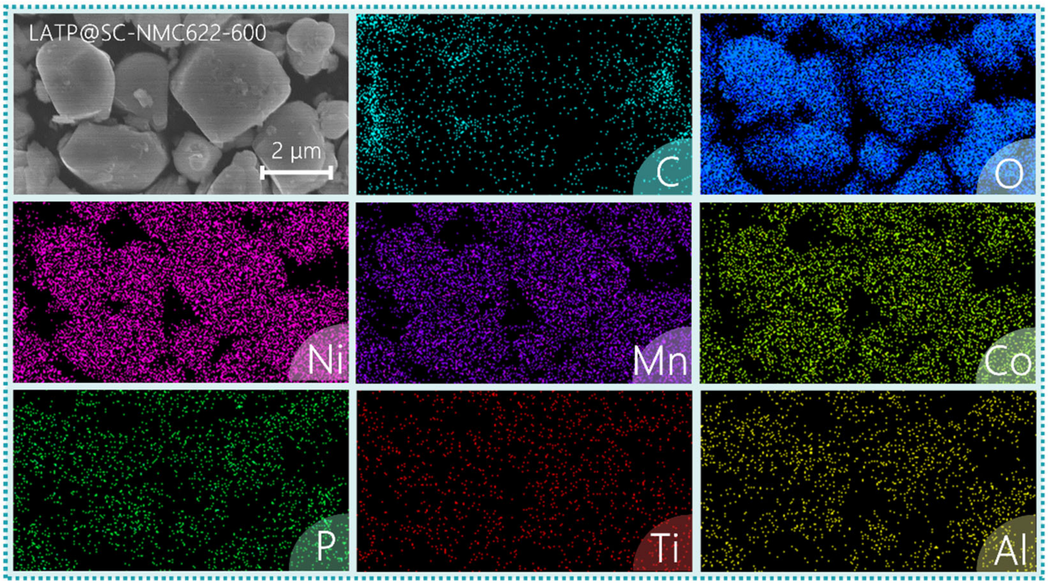

Supplementary Figure 63 **Element mapping of the LATP@SC-NMC622-600.**

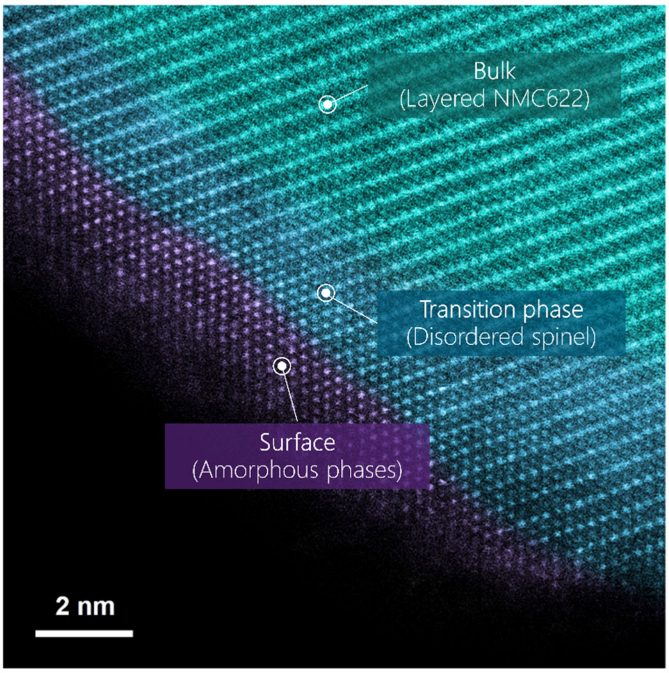

Supplementary Figure 64 **HADDF-STEM image of LATP@SC-NMC622-600 and structural evolution from surface to bulk.**

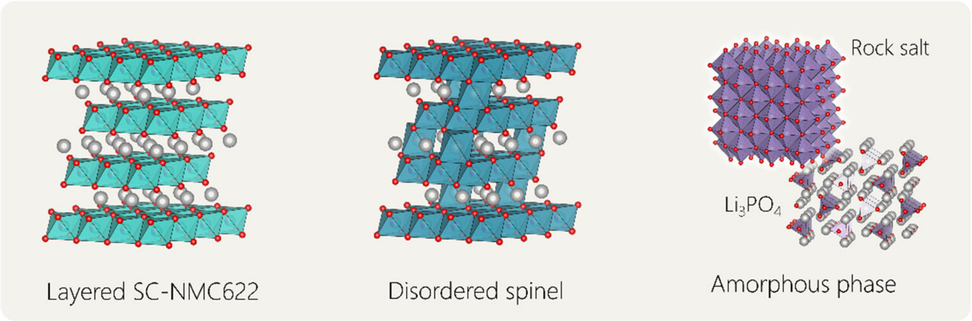

Supplementary Figure 65 **Schematic diagram of crystal structures identified in the STEM results.**

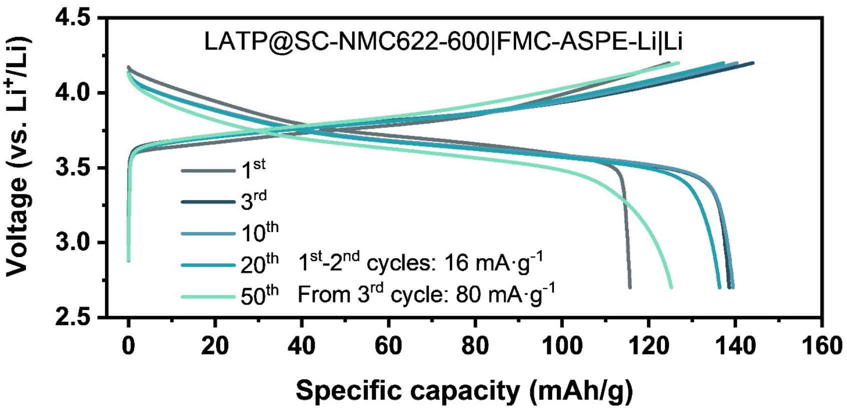

Supplementary Figure 66 **Charge/discharge curves of the LATP@SC-NMC622-600|FMC-ASPE-Li|Li coin cell (CR2032 coin cell, 70°C, 16 mA·g^{-1} for the first two cycles, 80 mA·g^{-1} from the 3rd cycle, 2.7-4.2 V, FMC-ASPE-Li composition: m_{LiTFSI} : m_{PEO} : $m_{21\text{-}\beta\text{-CD-}g\text{-PTFEMA}}$ = 0.6 : 2 : 1, $M_{n,\ GPC\ (21\text{-}\beta\text{-CD-}g\text{-PTFEMA})}$ = 900k, M_{PEO} = 600k).**

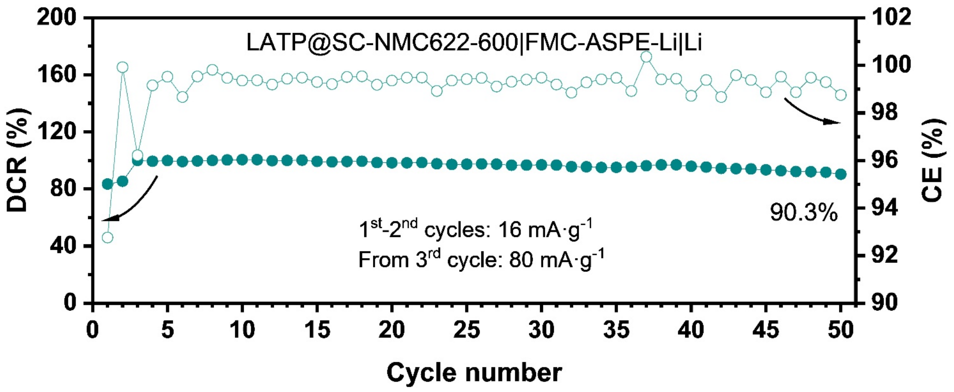

Supplementary Figure 67 **Capacity retention of the LATP@SC-NMC622-600|FMC-ASPE-Li|Li coin cell (CR2032 coin cell, 70°C, 16 mA·g^{-1} for the first two cycles, 80 mA·g^{-1} from the 3rd cycle, 2.7-4.2 V, FMC-ASPE-Li composition: m_{LiTFSI} : m_{PEO} : $m_{21\text{-}\beta\text{-CD-}g\text{-PTFEMA}}$ = 0.6 : 2 : 1, $M_{n,\ GPC\ (21\text{-}\beta\text{-CD-}g\text{-PTFEMA})}$ = 900k, M_{PEO} = 600k).**

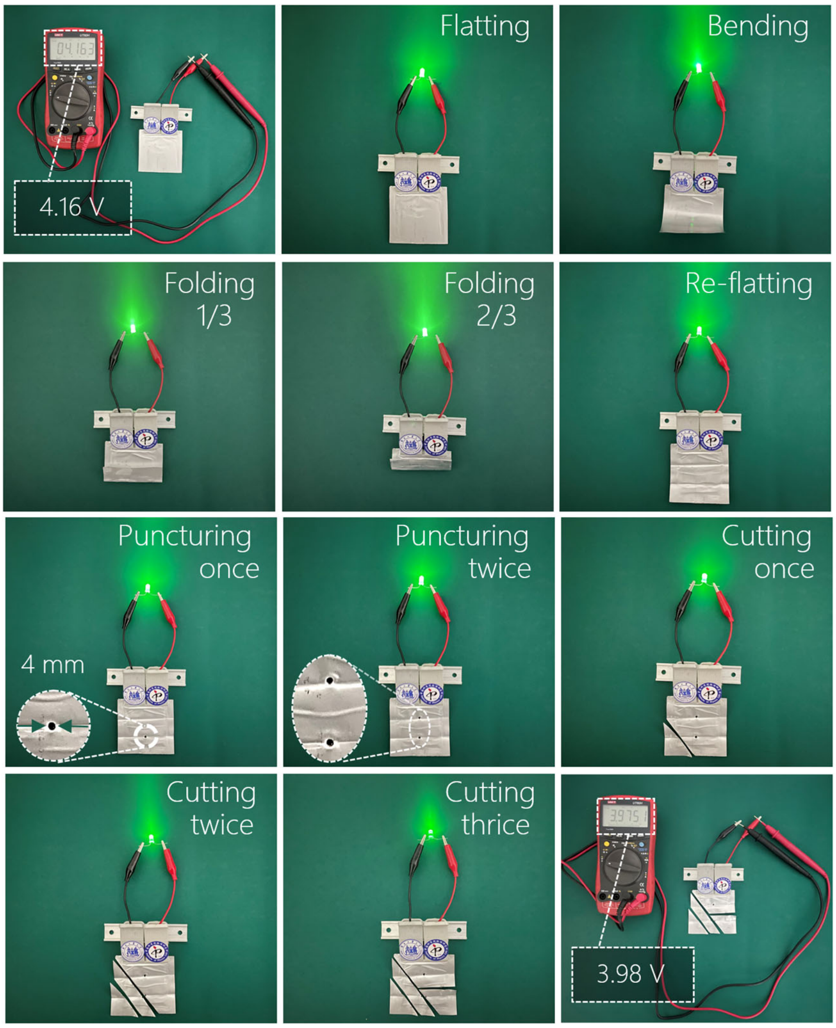

Supplementary Figure 68 The safety test results of the pouch cell (pouch cell, 70°C, 16 mA·g^{-1} charged to 4.2 V, , FMC-ASPE-Li composition: m_{LiTFSI} : m_{PEO} : $m_{21\text{-}\beta\text{-}CD\text{-}g\text{-}PTFEMA}$ = 0.6 : 2 : 1, $M_{n,\ GPC\ (21\text{-}\beta\text{-}CD\text{-}g\text{-}PTFEMA)}$ = 900k, M_{PEO} = 600k). SC-NMC622 was selected as the cathode material with relatively worse safety than LMFP. No abnormalities, such as smoking or fire evolution, occurred during such abusive operations, and the open-circuit voltage did not show a significant drop after all the safety tests.

Supplementary Table 1 The results of elemental analyses for the multifunctional macroinitiator 21-*β*-CD-Br.

Molecular formula	Elemental mass fractions (%)	
$C_{126}H_{175}O_{56}Br_{21}$	C	H
Theoretical value [(a)]	35.49	4.14
Found 1 [(b)]	36.11	4.12
Found 2 [(b)]	35.98	4.02
Mean values [(c)]	36.05	4.07

(a) The theoretical value of elemental mass fractions (%), (b) The estimated values of elemental mass fractions (%), (c) The estimated mean values of elemental mass fractions (%) for $C_{126}H_{175}O_{56}Br_{21}$. The results indicated that the C content was 36.05%, and the H content was 4.07%, which agreed well with the theoretical value of $C_{126}H_{175}O_{56}Br_{21}$ (C: 35.49%, H: 4.14%).

Supplementary Table 2 Analysis and results of the orthogonal L_4 (2^3) experimental design for lithium metal batteries.

Experiment	Factors			Result
	A	B	C	Capacity retention (%)
1	A1	B1	C1	64.06
2	A1	B2	C2	65.01
3	A2	B1	C2	71.20
4	A2	B2	C1	76.20

Supplementary Table 3 Analysis and results of orthogonal L_9 (3^4) sodium metal battery experimental design.

Experiment	Factors				Result
	A	B	C	D	Capacity retention (%)
1	A1	B1	C1	D1	17.2
2	A1	B2	C2	D2	81.7
3	A1	B3	C3	D3	85.4
4	A2	B1	C2	D3	76.6
5	A2	B2	C3	D1	85.7
6	A2	B3	C1	D2	25.0
7	A3	B1	C3	D2	81.3
8	A3	B2	C1	D3	91.1
9	A3	B3	C2	D1	78.7

Supplementary Table 4 Calculated bond energy and bond length for different hydrogen bonds.

Type of hydrogen bond	Bond energy		The bond length of F···H
	(eV)	($KJ\cdot mol^{-1}$)	(Å)
F···H-C	-0.0173	-1.672	2.36
F···H-O	-0.0535	-5.160	2.16

Supplementary Table 5 Mechanical property parameters of the composite SPE and pure PEO-based SPE for AS-LMBs and AS-SMBs.

Parameters	FMC-ASPE-Li	PEO-ASPE-Li	FMC-ASPE-Na	PEO-ASPE-Na	Unit
Tensile strength	2.0	1.6	1.7	1.3	MPa
Elongation at break	2198	649	1678	456	%
Toughness	3.36	0.91	1.85	0.55	10^4 $KJ\cdot m^{-4}$

The toughness of ASPEs can be calculated by integrating the area under tensile curves, as expressed by equation (2):

$$T = \int_0^{\varepsilon_b} s d\varepsilon \qquad (2)$$

where T is the toughness of ASPEs, ε is the strain, ε_b is the elongation at break, and s is stress.

Supplementary Table 6 **Ionic conductivity of FMC-ASPE-Li and PEO-ASPE-Li.**

Temperature (ºC)	σ (S·cm^{-1})	
	FMC-ASPE-Li	PEO-ASPE-Li
25	3.28±0.01E-06	4.49±0.05E-07
30	6.57±0.03E-06	8.44±0.10E-07
35	1.29±0.01E-05	1.49±0.01E-06
40	2.45±0.02E-05	2.79±0.02E-06
45	5.14±0.23E-05	4.92±0.05E-06
50	1.43±0.19E-04	9.55±0.11E-06
55	2.58±0.03E-04	2.37±0.03E-05
60	3.20±0.03E-04	1.02±0.01E-04
65	3.89±0.04E-04	1.07±0.01E-04
70	4.66±0.05E-04	1.22±0.01E-04
75	5.52±0.05E-04	1.39±0.02E-04
80	6.43±0.06E-04	1.49±0.02E-04

Supplementary Table 7 **Ionic conductivity of FMC-ASPE-Na and PEO-ASPE-Na.**

Temperature (ºC)	σ (S·cm^{-1})	
	FMC-ASPE-Na	PEO-ASPE-Na
25	2.59±0.13E-06	5.73±0.25E-07
30	5.07±0.21E-06	1.10±0.07E-06
35	9.51±0.41E-06	2.11±0.05E-06
40	1.68±0.06E-05	3.73±0.22E-06
45	2.98±0.11E-05	6.89±0.10E-06
50	5.28±0.18E-05	1.34±0.03E-05
55	1.34±0.07E-04	3.16±0.07E-05
60	4.54±0.07E-04	9.53±0.12E-05
65	5.36±0.08E-04	1.09±0.03E-04
70	6.31±0.09E-04	1.25±0.03E-04
75	7.34±0.08E-04	1.46±0.03E-04
80	8.44±0.09E-04	1.62±0.01E-04

Supplementary Table 8 Calculated adsorption energy for Li^+ with different adsorption sites.

Polymer	Adsorption site	Adsorption energy (eV)
PEO	-O-	-0.457
	-F-	-0.079
21-*β*-CD-*g*-PTFEMA	-O-	-0.323
	O=	-0.683

Supplementary Table 9 EIS fitting results of Li|PEO-ASPE-Li|Li for the transference number test

EIS fitting result of Li|PEO-ASPE-Li|Li before POL (70°C)				
Element	Definition	Value	Unit	Error (%)
Re	Resistance of electrolyte	12.85	Ω	0.52
Rf	Resistance of the passivating layer at the electrode/electrolyte interface	2.38	Ω	5.06
CPEf-T	Constant phase element of the passivating layer at the electrode/electrolyte interface	2.70E-7	$S \cdot s^{n} \cdot cm^{-2}$	5.53
CPEf-P		1.19	/	0.42
Rc	Resistance of charge transfer	46.35	Ω	0.38
CPEc-T	Constant phase element of charge transfer	7.05E-6	$S \cdot s^{n} \cdot cm^{-2}$	1.20
CPEc-P		0.87	/	0.10
χ^2	0.0045287			

EIS fitting result of Li|PEO-ASPE-Li|Li after POL (70°C)				
Element	Definition	Value	Unit	Error (%)
Re	Resistance of electrolyte	10.66	Ω	0.67
Rf	Resistance of the passivating layer at the electrode/electrolyte interface	4.34	Ω	4.19
CPEf-T	Constant phase element of the passivating layer at the electrode/electrolyte interface	4.94E-7	$S \cdot s^{n} \cdot cm^{-2}$	3.60
CPEf-P		1.11	/	5.45
Rc	Resistance of charge transfer	45.65	Ω	2.82
CPEc-T	Constant phase element of charge transfer	5.64E-6	$S \cdot s^{n} \cdot cm^{-2}$	1.47
CPEc-P		0.87	/	1.77
χ^2	0.0015106			

Supplementary Table 10 EIS fitting results of Li|FMC-ASPE-Li|Li for the transference number test

EIS fitting result of Li|FMC-ASPE-Li|Li before POL (70°C)				
Element	Definition	Value	Unit	Error (%)
Re	Resistance of electrolyte	44.01	Ω	0.38
Rf	Resistance of the passivating layer at the electrode/electrolyte interface	3.12	Ω	3.84
CPEf-T	Constant phase element of the passivating layer at the electrode/electrolyte interface	2.06E-7	$S \cdot s^n \cdot cm^{-2}$	7.18
CPEf-P		1.20	/	4.39
Rc	Resistances of charge transfer	61.69	Ω	2.63
CPEc-T	Constant phase element of charge transfer	3.48E-6	$S \cdot s^n \cdot cm^{-2}$	1.49
CPEc-P		0.87	/	1.67
χ^2	0.0004648			

EIS fitting result of Li|FMC-ASPE-Li|Li after POL (70°C)				
Element	Definition	Value	Unit	Error (%)
Re	Resistance of electrolyte	43.93	Ω	0.44
Rf	Resistance of the passivating layer at the electrode/electrolyte interface	3.60	Ω	4.10
CPEf-T	Constant phase element of the passivating layer at the electrode/electrolyte interface	2.32E-7	$S \cdot s^n \cdot cm^{-2}$	7.77
CPEf-P		1.17	/	4.84
Rc	Resistance of charge transfer	66.00	Ω	2.94
CPEc-T	Constant phase element of charge transfer	3.71	$S \cdot s^n \cdot cm^{-2}$	1.72
CPEc-P		0.86	/	1.92
χ^2	0.00069125			

Supplementary Table 11 Li-ion transference number for FMC-ASPE-Li and PEO-ASPE-Li.

Parameters	FMC-ASPE-Li	PEO-ASPE-Li	Unit
Li-ion transference number (t_{Li}^{+})	0.88	0.21	-
Polarization voltage (ΔV)	0.01	0.01	V
Initial current (I_o)	2.56E-05	1.45E-04	A
Steady state current (I_s)	2.27E-05	7.59E-05	A
The initial resistance of the electrolyte (Rb_o)	43.107	12.41	Ω
Final resistance of the electrolyte (Rb_s)	43.201	10.25	Ω
Initial interfacial resistance (R_o)	65.743	48.48	Ω
Steady-state interfacial resistance (R_s)	70.339	50.86	Ω

Supplementary Table 12 EIS fitting results of Na|PEO-ASPE-Na|Na for transference number test

EIS fitting result of Na|PEO-ASPE-Na|Na before POL (80°C)				
Element	Definition	Value	Unit	Error (%)
Re	Resistance of electrolyte	25.63	Ω	0.81
Rf	Resistance of the passivating layer at the electrode/electrolyte interface	6.19	Ω	6.24
CPEf-T	Constant phase element of the passivating layer at the electrode/electrolyte interface	1.01E-6	$S\cdot s^{n}\cdot cm^{-2}$	8.46
CPEf-P		1.10	/	5.74
Rc	Resistance of charge transfer	206.50	Ω	2.24
CPEc-T	Constant phase element of charge transfer	5.46E-6	$S\cdot s^{n}\cdot cm^{-2}$	1.65
CPEc-P		0.82	/	1.78
χ^2	0.0040925			
EIS fitting result of Na|PEO-ASPE-Na|Na after POL (80°C)				
Element	Definition	Value	Unit	Error (%)
Re	Resistance of electrolyte	23.55	Ω	0.56
Rf	Resistance of the passivating layer at the electrode/electrolyte interface	15.89	Ω	5.71
CPEf-T	Constant phase element of the passivating layer at the electrode/electrolyte interface	2.67E-6	$S\cdot s^{n}\cdot cm^{-2}$	2.34
CPEf-P		0.95	/	1.81
Rc	Resistance of charge transfer	208.30	Ω	2.69
CPEc-T	Constant phase element of charge transfer	4.33E-6	$S\cdot s^{n}\cdot cm^{-2}$	1.34
CPEc-P		0.86	/	1.57
χ^2	0.00052911			

Supplementary Table 13 EIS fitting results of Na|FMC-ASPE-Na|Na for transference number test

EIS fitting result of Na|FMC-ASPE-Na|Na before POL (80°C)				
Element	Definition	Value	Unit	Error (%)
Re	Resistance of electrolyte	21.62	Ω	1.06
Rf	Resistance of the passivating layer at the electrode/electrolyte interface	28.09	Ω	2.60
CPEf-T	Constant phase element of the passivating layer at the electrode/electrolyte interface	1.23E-6	$S\cdot s^n\cdot cm^{-2}$	2.79
CPEf-P		0.94	/	3.07
Rc	Resistance of charge transfer	273.10	Ω	1.51
CPEc-T	Constant phase element of charge transfer	3.09E-6	$S\cdot s^n\cdot cm^{-2}$	4.07
CPEc-P		0.83	/	2.80
χ^2	0.0017349			

EIS fitting result of Na|FMC-ASPE-Na|Na after POL (80°C)				
Element	Definition	Value	Unit	Error (%)
Re	Resistance of electrolyte	19.87	Ω	1.17
Rf	Resistance of the passivating layer at the electrode/electrolyte interface	30.13	Ω	2.72
CPEf-T	Constant phase element of the passivating layer at the electrode/electrolyte interface	1.23E-6	$S\cdot s^n\cdot cm^{-2}$	2.82
CPEf-P		0.94	/	3.03
Rc	Resistance of charge transfer	291.10	Ω	1.52
CPEc-T	Constant phase element of charge transfer	3.23E-6	$S\cdot s^n\cdot cm^{-2}$	4.16
CPEc-P		0.822	/	3.00
χ^2	0.0018654			

Supplementary Table 14 **Na-ion transference number for FMC-ASPE-Na and PEO-ASPE-Na.**

Parameters	FMC-ASPE-Na	PEO-ASPE-Na	Unit
Na-ion transference number (t_{Na^+})	0.71	0.20	-
Polarization voltage (ΔV)	0.01	0.01	V
Initial current (I_o)	2.25E-05	4.01E-05	A
Steady state current (I_s)	1.97E-05	2.31E-05	A
The initial resistance of the electrolyte (Rb_o)	21.54	25.727	Ω
Final resistance of the electrolyte (Rb_s)	19.793	23.444	Ω
Initial interfacial resistance (R_o)	301.29	203.783	Ω
Steady-state interfacial resistance (R_s)	322.157	221.736	Ω

Supplementary Table 15 Simulation parameters of the composite SPE and pure PEO-based SPE for AS-LMBs.

Parameters	FMC-ASPE-Li	PEO-ASPE-Li	Unit
Ionic conductivity (σ)	4.65E-04	1.22E-04	$S·cm^{-1}$
Salt concentration (c)	500	500	$mol·m^{-3}$
Transference number (t_{Li}^{+})	0.88	0.21	-
Cation diffusion coefficient (D^{+})	2.52E-11	1.58E-12	$m^2·s^{-1}$
Anion diffusion coefficient (D^{-})	3.43E-12	5.93E-12	$m^2·s^{-1}$

Supplementary Table 16 Simulation conditions of the composite SPE and pure PEO-based SPE for AS-LMBs.

Parameters	FMC-ASPE-Li	PEO-ASPE-Li	Unit
Width of solid electrolyte (w)	50		μm
The thickness of solid electrolyte (H)	200		μm
Operating temperature (T)	70		°C
Applied voltage (U)	0.01		V
Time (t)	300		s

Supplementary Table 17 Simulation parameters of the composite SPE and pure PEO-based SPE for AS-SMBs.

Parameters	FMC-ASPE-Na	PEO-ASPE-Na	Unit
Ionic conductivity (σ)	6.30E-04	1.25E-04	$S\cdot cm^{-1}$
Salt concentration (c)	400	400	$mol\cdot m^{-3}$
Transference number ($t_{Na}{}^{+}$)	0.71	0.2	-
Cation diffusion coefficient (D^{+})	3.54E-11	1.98E-12	$m^2\cdot s^{-1}$
Anion diffusion coefficient (D^{-})	1.45E-11	7.91E-12	$m^2\cdot s^{-1}$

Supplementary Table 18 Simulation conditions of the composite SPE and pure PEO-based SPE for AS-SMBs.

Parameters	FMC-ASPE-Na	PEO-ASPE-Na	Unit
Width of solid electrolyte (w)	50		μm
The thickness of solid electrolyte (H)	200		μm
Operating temperature (T)	80		°C
Applied voltage (U)	0.01		V
Time (t)	300		s

Supplementary Table 19 **Comparison of cycling performance of different all-solid-state lithium batteries (specific current applied during cycling, voltage range, thickness of solid-state electrolyte (SSE), test temperature, cell type, external pressure are listed in the "Test details" column)**

Cathode|Electrolyte| Anode	Cycle number	Capacity retention (%)	Test details	Ref.
NMC111|LSPS|Li	75	84	10 $mA\cdot g^{-1}$, 2.5-4.3 V, SSE thickness 1.5 mm, 25°C, Swagelok cell, 75 MPa	5
LCO|LGPS|Li	50	91	10 $mA\cdot g^{-1}$, 3.0-4.2 V, 25°C, Swagelok cell, 250 MPa	6
LCO|LGPS+LS+PS|Li	25	90	16 $mA\cdot g^{-1}$, 3.3-4.3 V, SSE thickness 0.9 mm, 30°C, Swagelok cell	7
LCO|LGPS+LPSSO|Li	50	78.6	12 $mA\cdot g^{-1}$, 3.0-4.3 V, SSE thickness 1.0 mm, 25°C, Swagelok cell, 240 MPa	8
NCA|LS+PS|Li	20	100	3 $mA\cdot g^{-1}$, 2.0-4.0 V, 25°C, Swagelok cell	9
NMC622|LPSI|Li	40	74.3	17.5 $mA\cdot g^{-1}$, 2.5-4.3 V, SSE thickness 0.5 mm, 55°C, Swagelok cell, 30 MPa	10
LCO|LLZTO|Li	75	42	0.1 $mA\cdot cm^{-2}$, 2.5-4.3 V, SSE thickness 0.5 mm, 100°C, Swagelok cell	11
LFP|PEO-LiTFSI+LLZTAO|Li	10	100	0.1 $mA\cdot cm^{-2}$, 2.3-3.8 V, SSE thickness ~70 μm, 60°C, LIR 2032 coin cell	12
NMC622|PBA-LClO+LAGP|Li	100	78	34 $mA\cdot g^{-1}$, 3.0-4.3 V, SSE thickness 1.0 mm, 55°C, CR2032 coin cell	13

LCO|LLZO+PE-LiTFSIO|Li	20	95.6	0.05 C, 3.0-4.2 V, SSE thickness 0.2 mm, 50°C, Swagelok cell	14
NMC622|LPS+PEO-LiTFSI|Li	50	90	7.5 $mA\cdot g^{-1}$, 3.0-4.2 V, SSE thickness <0.4 mm, 60°C, Swagelok cell	15
LFP|PEO-LiTFSI|Li	1400	80	C/3, 2.5-4.0 V, SSE thickness 0.02 mm, 70°C, pouch cell, 0.24 MPa	16
LFP|SI+PEO-LiTFSI|Li	300	77.8	85 $mA\cdot g^{-1}$, 2.5-3.8 V, SSE thickness 15-20 μm, 70°C, ECC-Std cell	17
LCO|PEO-LiTFSI+CO|Li	40	80	35 $mA\cdot g^{-1}$, 2.5-4.3 V, SSE thickness 120 μm, 60°C, CR2032 coin cell	18
NMC622|MCF-SPE|Li	15	91	0.1C, 3.0-4.2 V, 70°C, CR2032 coin cell	19
LMFP|PEO+CMOF-LiTFSI|Li	100	81.2	0.1C, 2.8-3.8 V, SSE thickness 40 μm, 60°C, CR2025 coin cell	20
**NMC811|FMC-ASPE-Li|Li**	**100**	**87**	80 $mA\cdot g^{-1}$, 2.7-4.2 V, ASPE thickness ~160 μm, 70°C, CR2032 coin cell, 6 MPa	**This work**
**LMFP|FMC-ASPE-Li|Li**	**200**	**91**	70 $mA\cdot g^{-1}$, 2.5-4.2 V, ASPE thickness ~160 μm, 70°C, CR2032 coin cell, 6 MPa	**This work**
**LFP|FMC-ASPE-Li|Li**	**400**	**98**	70 $mA\cdot g^{-1}$, 2.5-3.8 V, ASPE thickness ~160 μm, 70°C, CR2032 coin cell, 6 MPa	

Note: LCO, $LiCoO_2$; NCM622, $Li(Ni_{0.6}Co_{0.2}Mn_{0.2})O_2$; NCM111, $Li(Ni_{0.3}Co_{0.3}Mn_{0.3})O_2$; NCA, $Li(Ni_{0.8}Co_{0.15}Al_{0.05})O_2$; PEO, poly(ethylene oxide); LFP, $LiFePO_4$; LMFP, $LiMn_{0.6}Fe_{0.4}PO_4$ or $LiMn_{0.85}Fe_{0.15}PO_4$; PBA, poly(1,4-butylene adipate); SI, single-ion; LiTFSI, lithium bis(trifluoromethanesulfonyl)imide ($LiC_2F_6NO_4S_2$); LClO, $LiClO_4$; LSPS, $Li_{10}SiP_2S_{12}$; LGPS, $Li_{10}GeP_2S_{12}$ or $Li_{3.15}Ge_{0.15}P_{0.85}S_4$; LS, Li_2S; PS, P_2S_5; LPSSO, $Li_3P_{1-x}Sb_xS_{4-2.5x}O_{2.5x}$; LPSI, $Li_7P_2S_8I$;

LLZTO, $Li_{1.3}Al_{0.3}Ti_{1.7}(PO_4)_3$; LLZTAO, Al-$Li_{6.75}La_3Zr_{1.75}Ta_{0.25}O_{12}$; LPS, β-$Li_3PS_4$; MCF-SPE, main-chain fluorinated solid polymer electrolytes; CMOF; D-UiO-66-NH_2; CO; CeO_2.

Supplementary Table 20 **Comparison of some typical strategies to improve the PEO-based solid-state electrolytes**

Strategies	Li^+ conductivity	Li^+ transference number	Cathode materialss	Tensile strength	Ref.
Blending nano-SiO_2	3E-4 $S\cdot cm^{-1}$ (60°C)	0.19 (100°C)	/	0.5-2.5 MPa	21
In Situ blending nano-SiO_2	1.2E-3 $S\cdot cm^{-1}$ (60°C)	/	$LiFePO_4$	/	22
Blending $Li_7La_3Zr_2O_{12}$	4.42E-4 $S\cdot cm^{-1}$ (55°C)	/	$LiNi_{0.6}Mn_{0.2}Co_{0.2}O_2$	/	23
Blending polysulfide-bridged copolymers	2.13E-4 $S\cdot cm^{-1}$ (50°C)	0.58 (50°C)	$LiFePO_4$	/	24
Blending $Pyr_{1,(2O)7}TFSI$ (ionic liquid)	1.4E-3 $S\cdot cm^{-1}$ (60°C)	0.1 (40°C)	$LiFePO_4$	/	25
Designing new salt, LiPSTFSI	~E-4 $S\cdot cm^{-1}$ (70°C)	0.63	$LiFePO_4$	/	26
Blending D-UiO-66-NH_2 (CMOF)	6.3E-4 $S\cdot cm^{-1}$ (60°C)	0.72 (60°C)	$LiFePO_4$, $LiFe_{0.15}Mn_{0.85}PO_4$ (81.2% @100th cycle)	2.5-9.7 MPa	20
In situ polymerization of PEGdMA in PEO	2.24E-5 $S\cdot cm^{-1}$ (60°C)	/	$LiNi_{0.6}Mn_{0.2}Co_{0.2}O_2$, (~70% @100th cycle)	0.28 MPa	27
Blending Ca-doped CeO_2	1.3E-4 $S\cdot cm^{-1}$ (60°C)	0.45 (60°C)	$LiFePO_4$, $LiCoO_2$ (80% @40th cycle)	10.2 MPa	18
**Blending 21-*β*-CD-*g*-PTFEMA**	**1.34E-4 $S\cdot cm^{-1}$ (60°C)**	**0.88 (70°C)**	**$LiFePO_4$ (98% @400th cycle), $LiNi_{0.8}Mn_{0.1}Co_{0.1}O_2$ (87% @100th cycle), $LiMn_{0.6}Fe_{0.4}PO_4$ (91% @200th cycle)**	**2 MPa**	**This work**

Supplementary Note 1: the abbreviation list

The abbreviation list for details about the full name of technical terms in the main text

Numbers [(a)]	Abbreviations	Full names
1	21-*β*-CD-Br	heptakis [2,3,6-tri-*O*-(2-bromo-2-methylpropionyl]-*β*-cyclodextrin
2	21-*β*-CD-*g*-PTFEMA	21-arm poly(2,2,2-trifluoroethyl methacrylate)
3	AS-LMBs	all-solid-state lithium metal batteries
4	AS-SMBs	all-solid-state sodium metal batteries
5	ATRP	atom transfer radical polymerization
6	BIBB	2-bromoisobutyryl bromide
7	Đ	polydispersity index
8	DC	direct-current
9	DFT	density functional theory
10	EIS	electrochemical impedance spectrum
11	FEMS	finite element method simulations
12	FMC-ASPE	fluorine-rich macromolecule-containing all-solid-state polymer electrolyte
13	FMC-ASPE-Li	fluorine-rich macromolecule-containing all-solid-state polymer electrolyte for LMBs
14	FMC-ASPE-Na	fluorine-rich macromolecule-containing all-solid-state polymer electrolyte for SMBs
15	FTIR	Fourier transform infrared spectroscopy
16	GPC	gel permeation chromatography
17	HOMO	highest occupied molecular orbital
18	ISEs	inorganic solid-state electrolytes
19	LFP	$LiFePO_4$
20	LMFP	$LiMn_{0.6}Fe_{0.4}PO_4$
21	LIBs	lithium-ion batteries
22	LSV	linear sweep voltammetry
23	NMR	nuclear magnetic resonance
24	NVP	$Na_3V_2(PO_4)_3$
25	NVOPF	$Na_3(VOPO_4)_2F$
26	NCNFM	$NaCu_{1/9}Ni_{2/9}Fe_{1/3}Mn_{1/3}O_2$
27	PEO-ASPE-Li	pure PEO-based ASPEs for LMBs
28	PEO-ASPE-Na	pure PEO-based ASPEs for SMBs
29	PEO-SPEs	poly(ethylene oxide)-based solid polymer electrolytes
30	POL	polarization
31	PTFEMA	poly(2,2,2-trifluoro-ethyl methacrylate)
32	PVC	poly(vinylene carbonate)
33	*s*	tensile strength
34	SC-NMC622	single-crystal $LiNi_{0.6}Mn_{0.2}Co_{0.2}O_2$
35	SC-NMC811	single-crystal $LiNi_{0.8}Mn_{0.1}Co_{0.1}O_2$

36	SEM	scanning electron microscope
37	SPEs	solid-state polymer electrolytes
38	SSBs	solid-state batteries
39	TFEMA	2,2,2-trifluoroethyl methacrylate
40	TG-FTIR	thermogravimetry-Fourier transform infrared spectroscopy
41	t_{Li^+}	lithium-ion transference number
42	t_{Na^+}	sodium-ion transference number
43	XPS	X-ray photoelectron spectroscopy
44	XRD	X-ray diffraction
45	β-CD	β-Cyclodextrin
46	ε_b	elongation at break
47	σ	ion conductivity

(a) The abbreviations were coded numerically and alphabetically in ascending order.

The abbreviation list for details about the full name of technical terms in the supplementary information

Numbers [(a)]	Abbreviations	Full names
1	*β*-CD	*β*-cyclodextrin
2	^{1}H NMR	proton nuclear magnetic resonance
3	^{13}C NMR	carbon nuclear magnetic resonance
4	^{19}F NMR	fluorine nuclear magnetic resonance
5	21-Br-*β*-CD	heptakis [2,3,6-tri-*O*-(2-bromo-2-methylpropionyl]-*β*-cyclodextrin
6	21-*β*-CD-*g*-PTFEMA	21-arm poly(2,2,2-trifluoroethyl methacrylate)
7	ATR-FTIR	attenuated total reflection-Fourier transform infrared spectroscopy
8	BIBB	2-bromoisobutyryl bromide
9	bpy	2, 2’ -bipyridyl
10	CaH_2	calcium hydride
11	CH_2Cl_2	methylene chloride
12	CuCl	copper (I) chloride
13	EDX	energy-dispersive X-ray
14	EIS	electrochemical impedance spectroscopy
15	FE-SEM	field-emission scanning electron microscopy
16	FIB	focused ion beam
17	FTIR	Fourier transform infrared spectra
18	GPC	gel permeation chromatography
19	HAADF-STEM	high-angle annular dark-field scanning transmission electron microscopy
20	LATP	$Li_{1.5}Al_{0.5}Ti_{1.5}(PO_4)_3$
21	LATP@SC-NMC622	LATP modified SC-NMC622 material
22	LFP	$LiFePO_4$
23	LiTFSI	bis(trifluoromethanesulfonyl)imide lithium salt
24	LSV	linear sweep voltammetry
25	$NaClO_4$	sodium perchlorate
26	$NaHCO_3$	sodium bicarbonate
27	$NaPF_6$	sodium hexafluorophosphate
28	NaTFSI	bis(trifluoromethanesulfonyl)imide sodium salt
29	NMP	anhydrous 1-methyl-2-pirrolidone
30	NCNFM	$NaCu_{1/9}Ni_{2/9}Fe_{1/3}Mn_{1/3}O_2$
31	NVP	$Na_3V_2(PO_4)_3$
32	PC-NMC622	polycrystalline $LiNi_{0.6}Mn_{0.2}Co_{0.2}O_2$
33	PEO	polyethylene oxide
34	PVDF	poly(vinylidene fluoride)

35	SC-NMC622	single-crystal $LiNi_{0.6}Mn_{0.2}Co_{0.2}O_2$
36	SS	stainless steel
37	Super-P	conductive carbon black
38	TFEMA	2,2,2-trifluoroethyl methacrylate
39	TGA	thermogravimetric analysis
40	TG-FTIR	thermogravimetry-Fourier transform infrared spectroscopy
41	THF	tetrahydrofuran
42	XPS	X-ray photoelectron spectroscopy
43	XRD	X-ray powder diffraction

(a) The abbreviations were coded numerically and alphabetically in ascending order.

Supplementary Note 2: Design principles for FMC-ASPEs

The following design principles, shown in Supplementary Figure 1, can be considered to address the challenges of poor high-voltage stability, low transference number, low ionic conductivity, and low mechanical strength of PEO-ASPEs. First, we chose to add a topological homopolymer to PEO-ASPEs based on considering the wide selection of functional monomers and preserving the existing advantages of PEO-ASPEs. Moreover, supramolecular self-assembly triggered by specific functional groups could introduce additional benefits, which have often been neglected[28]. As a potentially suitable monomer, 2,2,2-trifluoroethyl methacrylate (TFEMA) can control the macromolecular structure and multifaceted tuning of the material properties. In addition to its intrinsic properties, such as low flammability, good film-forming behaviour, high thermal stability, attractive toughness, and desired chemical resistance[29], the following three properties of TFEMA are highly beneficial to the resulting electrochemical properties: the low highest occupied molecular orbital (HOMO) level, which endows the high-voltage stability of ASPEs[30], the multiple hydrogen bonds formed between C-F and PEO, and the loose O-Li^+ coordination between O=C and lithium ions, which improves σ_{Li^+} and t_{Li^+}[31]. To obtain a well-defined fluoropolymer with an accurately controllable molecular weight and narrow polydispersity index (Đ), atom transfer radical polymerization (ATRP), one of the most efficient and widely used controlled/"living" radical polymerization techniques[32], is the optimal approach for polymerization. The rational design and control over a topological structure can stimulate fluoropolymers to achieve valuable performance beyond their inherent composition. Owing to their multi-arm and three-dimensional spherical structure, fluoropolymers exhibit unique physical properties and electrochemical behaviours that are unattainable with simple linear homopolymers[33]. Such behaviours include the feasibility of generating high-molecular-weight poly(2,2,2-trifluoroethyl methacrylate) (PTFEMA), inhibition of polymer crystallization, additional free volume for segment motion, excellent mechanical properties, high ionic conductivity, and good lithium salt solubility for ASPEs. To obtain high-yield fluoropolymers through ATRP, the "core-first" approach outperforms other existing approaches. Nevertheless, current studies on multifunctional initiators (i.e., the "core") have significant limitations, such as small molecular sizes and low functionalities (typically ranging from 3 to 8)[34], restricting both the core dimension and the arm numbers of the resulting fluoropolymers. One modification of particular interest is the complete esterification of 21 hydroxyl groups for *β*-cyclodextrin (*β*-CD), which possesses the rigid steric structure of a truncated conical shape with an inner cavity, and each molecule contains 21 substitutable active sites for ATRP[35].

Supplementary Note 3: analysis of ^{1}H NMR and XPS results for 21-β-CD-Br

The ^{1}H NMR spectra of β-CD and 21-β-CD-Br are shown in Figure 1f; a new peak appears in the signal corresponding to the ethyl protons (b, 126H, -C$(CH_3)_2$Br) of the 2-bromoisobutyryl groups, which are located at a chemical shift of δ = 1.75-2.25 ppm. The signals located at the broad chemical shifts in the region of δ = 3.35-5.55 ppm are mainly associated with the inner methylidyne and methylene protons (1-6, 49H, residues of β-CD) between the carbon moieties and/or oxygen moieties on the glucose units of β-CD. The hydroxyl group conversion was calculated using the following equation (3):

$$C_{\mathrm{OH}}=\frac{IA_{\mathrm{b}}}{18IA_{\mathrm{a}}}\times 100\% \tag{3}$$

where C_{OH} is the conversion efficiency of the hydroxyl groups on β-CD, and IA_{b} and IA_{a} represent the integral area of the methyl protons of 21-β-CD-Br (b, 126H, the peaks at δ = 1.75-2.25) and the integral area of the protons (a, 7H, the peaks at δ = 5.15-5.35), respectively. The obtained C_{OH} value of 100% indicated that the hydroxyl groups were completely converted into 21 bromoisobutyryl units. Further confirmation of the C_{OH} value was also calculated based on ^{13}C NMR and found to be 100% (Supplementary Figure 4).

In addition, XPS analysis was carried out to monitor the change in the elemental composition of modified β-CD. The existence of bromine atoms in 21-β-CD-Br was confirmed based on the overall XPS spectra shown in Figure 1b and the Br 3*d* core-level spectrum shown in Figure 1c. Furthermore, by comparing the C 1*s* core-level spectra of β-CD and 21-β-CD-Br displayed in Figures 1d and 1e, respectively, a conspicuous O=C-O peak assigned to the ester bonds linking the β-CD ring and the bromoisobutyryl units was found, while the O-C-O peak attributed to β-CD was clearly weakened, indicating the successful synthesis of 21-β-CD-Br. Additional O 1*s* core-level spectra of β-CD and 21-β-CD-Br and a more detailed analysis can be found in Supplementary Figure 3. Beyond the above results, the FTIR spectra, elemental analysis, and SEM-EDS results also robustly demonstrated that the multifunctional macroinitiator 21-β-CD-Br was accurately synthesized; these detailed descriptions are summarized in Supplementary Figures 5 and 6, and Supplementary Table 1.

Supplementary Note 4: analysis ^{1}H NMR, ^{19}F NMR, FTIR, XPS, SEM-EDS and GPC results for 21-*β*-CD-*g*-PTFEMA

As shown in Figure 1f, the characteristic broad peak at δ = 0.75 to 1.41 ppm is attributed to the methyl protons in the alpha position of 21-*β*-CD-*g*-PTFEMA (e, -CH-C*(CH₃)*-), the signal at δ = 1.78 to 2.30 ppm is assigned to the methylidyne protons of PTFEMA (d, -*CH*-C(CH_3)-) and the feature peak at δ = 4.18-4.50 ppm is associated with the methylene protons of the 21-*β*-CD-*g*-PTFEMA ester side chain (c, -O$CH_2$$CF_3$). Additionally, the narrow and sharp ^{19}F NMR signals at 73.8 ppm corresponded to the -CF_3 group (Supplementary Figure 7). The characteristic band in the FTIR spectrum at 1740 cm^{-1} corresponds to ester carbonyl (C=O) bonds, and the peaks at 660 cm^{-1} and 1281 cm^{-1} are assigned to the stretching and bending vibrations of the C-F bonds, respectively (Supplementary Figure 8). The XPS F 1*s* spectrum illustrates a strong fluorine signal at 689.0 eV, where the detailed spectra of C 1*s* and O 1*s* can be found in Supplementary Figure 9. In addition, SEM-EDS mapping confirms a uniform distribution of fluorine in the 21-*β*-CD-*g*-PTFEMA particle, along with other elements (Supplementary Figure 10). The well-defined structure of 21-*β*-CD-*g*-PTFEMA was further evidenced based on GPC. The Đ remains low (< 1.25) for all 21-*β*-CD-*g*-PTFEMA products with high molecular weights (up to 900 kDa) (Supplementary Figure 11), indicating a controlled molecular architecture polymerized by ATRP.

Supplementary Note 5: Analysis of the orthogonal experimental design for FMC-ASPE-Li

To understand the properties of the FMC-ASPEs, reference samples of the pure PEO-based ASPEs for AS-LMBs and AS-SMBs (PEO-ASPE-Li and PEO-ASPE-Na) were also prepared with the same salt/polymer mass ratio as the optimal one. These two ASPEs for AS-LMBs were fully characterized based on scanning electron microscopy (SEM), FTIR, and X-ray diffraction (XRD). SEM micrographs of the two ASPE membranes are shown in Supplementary Figure 24. In contrast to the PEO-ASPE-Li membrane, the FMC-ASPE-Li membrane showed a much smoother surface and higher bulk density, indicating that the addition of 21-*β*-CD-*g*-PTFEMA to PEO-ASPE-Li was conducive to promoting the film-forming ability of SPE; this promotion could restrain dendrite growth on the Li anodes. The FTIR spectra are shown in Supplementary Figure 25. In addition to the characteristic peaks produced for the PEO-ASPE-Li membrane, a new peak at ~1753 cm^{-1} appears in the spectrum of the FMC-ASPE-Li membrane; this peak is consistent with the ester carbonyl (C=O) bonds. Additionally, the peaks at 660 cm^{-1} and 1281 cm^{-1} gradually become more intense as the addition of 21-*β*-CD-*g*-PTFEMA to PEO-ASPE-Li is assigned to the stretching and bending vibrations of C-F bonds, respectively. Notably, the C=O (free) absorption peak (~1753 cm^{-1}) in the 21-*β*-CD-*g*-PTFEMA FTIR spectra shifts to a relatively low wavenumber position (~1747 cm^{-1}) after composition with LiTFSI salt and PEO, showing typical C=O-Li^+ bonding characteristics with nearly all C=O bonds[36-38]. This result indicates the contribution of the C=O group to the Li^+ conductivity. In Supplementary Figure 26, a broad diffraction peak at approximately 20° was observed in the XRD pattern of 21-*β*-CD-*g*-PTFEMA, indicating an amorphous structure. Moreover, a similar broad diffraction peak can also be maintained in the FMC-ASPE-Li membrane, which reduces the crystallinity upon 21-*β*-CD-*g*-PTFEMA confinement. Furthermore, the XPS results further confirm the successful preparation of the FMC-ASPE-Li membrane (Supplementary Figure 27).

Supplementary Note 6: Introductions of the Chazalviel's model and "Sand's Time"

Driven by a more profound understanding, Chazalviel's model was chosen to explain the impact of the cation transfer number on the growth of lithium dendrites[39]. This theory suggests that cation depletion near the Li anode region could induce an inhomogeneous electric field, which will cause an uneven distribution of Li^+ flux and lead to the growth of ramified metallic electrodeposits. Quantitative analysis was explored by "Sand's time" [40], which can predict the appearance time of dendrites. As shown in the following equation (4), dendrites appear at the moment when the Li^+ concentration at the electrode interface drops to zero.

$$\tau = \pi D \left(\frac{C_0 e}{2J}\right)^2 \left(\frac{\mu_a + \mu_{Li^+}}{\mu_a}\right)^2 \tag{4}$$

where τ is the initial time of dendrite growth, D is the ambipolar diffusion coefficient, C_0 is the initial ionic concentration, e is the electronic charge, J is the local current density, and μ_a and μ_{Li^+} are the mobilities of the anion and Li^+, respectively. According to this equation, τ could be delayed by increasing μ_{Li^+} or decreasing μ_a, and the growth of dendrites could be suppressed. Thus, FMC-ASPE-Li with a high cation transfer number can prolong the time of Li dendrite nucleation, address dendrite growth and avoid internal short circuits.

Supplementary Note 7: Coated LATP SC-NMC622 for better interface stability

The overall performance of the all-solid-state, high-voltage-resistant AS-LMBs assembled based on FMC-ASPE can be further improved by modifying each component of the battery, including surface coating of the cathode material to form a high-voltage stable layer[41], adding other functional macromolecules with a particular topological structure to regulate the ASPEs and constructing a 3D rigid framework to protect the lithium metal negative electrode[42,43], etc. Here, to further improve the high-voltage stability of FMC-ASPE, the improvement of the cathode material was specifically investigated through the surface coating method. Supplementary Figure 60 schematically illustrates the two-step process employed in interface engineering for SC-NMC622. The detailed synthesis method is summarized in the Supporting Information. The X-ray diffraction (XRD) profiles of SC-NMC622, the sample surface treated with nano $Li_{1.4}Al_{0.4}Ti_{1.6}(PO_4)_3$ (LATP) particles (LATP@SC-NMC622-600), and LATP are compared and shown in Supplementary Figure 61, which indicates that the bulk structures of SC-NMC622 and LATP@SC-NMC622-600 are almost the same. The morphology change of the as-prepared LATP@SC-NMC622-600 was studied by scanning electron microscopy (SEM), as shown in Supplementary Figure 62. The LATP coating layer diffused into the interior of SC-NMC622 after annealing at 600°C, and a relatively rough layer with small uniform particles was observed on the surface of LATP@SC-NMC622-600. The energy dispersive X-ray spectrometry (EDS) elemental mapping results (Supplementary Figure 63) showed that P, Ti, Al, and Li elements were distributed uniformly in the selected area, demonstrating a homogenous coating layer on the particle surface.

High-angle annular dark-field scanning transmission electron microscope (HAADF-STEM) imaging, which is a powerful tool to distinguish the local structure from the bulk to the surface, was conducted to obtain more insights into the overall structure distribution of LATP@SC-NMC622-600, combined with the focused ion beam (FIB) sample preparation method. As shown in Supplementary Figures 64 and 65, the structural characteristics gradually changed from the bulk to the very surface, showing a three-layer composite structure (layered, disordered spinel, and amorphous phases) without hard borders. The main distinctions among the three structures were about the occupying rate of the transition metal atoms in the Li layer, and the randomly distributed bright spots on the very surface as shown in HAADF-STEM (mauve area) combined with the EDS result indicating the formation of rock-salt and the Li_3PO_4 on the very surface, which are of highly chemically stable and have appropriate Li^+ conductivity.

Cycling tests of the coin cell with the modified LATP@SC-NMC622-600 cathode, FMC-ASPE-Li membrane, and lithium metal anode were carried out in the voltage range of 2.7-4.2 V (**80 mA·g^{-1}**, 70°C), as shown in Supplementary Figures 66 and 67. As expected, the cycling performance was significantly improved, and a high capacity retention of 90.3% (50th cycle) was achieved, which was even higher than that of the SC-NMC622|FMC-ASPE-Li|Li full cell (80.9%). This result shows robust resistance to high voltage and confirms the upgraded capacity of the FMC-ASPE-Li membrane.

Supplementary Note 8: Comparison of the typical strategies to improve the PEO-ASPE performance

Here we compare some typical strategies to improve the PEO-ASPE performance, including blending inorganic fillers, blending organic materials, adding topological polymers, modifying the PEO matrix, framework engineering, designing new salts, and constructing stable structure interphases.

As shown in Supplementary Table 20, some of the performance of electrolytes could be significantly improved. Nevertheless, there are non-negligible limitations to the techniques used above. Notably, blending inorganic fillers with PEO-SPEs is prone to agglomerate, which inhibits the formation of uniform and dense membranes, resulting in Li dendrite growth along with defective orientation. In addition, in engineering and synthesizing new materials, including topochemical polymerization, post-modification of PEO-based functional groups, and designing new lithium salts, the cumbersome multistep integration process and technical demands should often be taken into consideration. Furthermore, the construction of multifunctional architectures and interphases are susceptible to strategies for modification, leading to the presence of unreacted monomers during in situ polymerization and the abruptly changing composition in the structure of SPEs usually causes the formation of defects and grain boundaries. Although the proper combination of multiple strategies could be helpful for comprehensive performance, the increased technical difficulty of feasibility is detrimental to large-scale production. Therefore, there is an urgent need to explore a simple preparation strategy that can improve the performance of PEO-SPEs. As shown in Supplementary Table 20, our strategy of blending 21-*β*-CD-*g*-PTFEMA with PEO exhibits comprehensive and improvements. Beyond these, the assembled LMFP|FMC-ASPE-Li|Li pouch cell exhibits great potential for application, showing a high working voltage and long cycling life.

Supplementary References

1 Ohno, K., Wong, B. & Haddleton, D. M. Synthesis of well-defined cyclodextrin-core star polymers. *J. Polym. Sci., Part A: Polym. Chem.* **39**, 2206-2214 (2001).

2 H, Y. W., T, K. E. & G, N. K. Controlled grafting of well-defined polymers on hydrogen-terminated silicon substrates by surface-initiated atom transfer radical polymerization. *J. Phys. Chem. B* **107**, 10198-10205 (2003).

3 K, M., G, G. S. & A, K. Preparation of hyperbranched polyacrylates by atom transfer radical polymerization. 1. acrylic AB monomers in "living" radical polymerizations. *Macromolecules* **30**, 5192-5194 (1997).

4 Li, J. & Xiao, H. An efficient synthetic-route to prepare [2,3,6-tri-O-(2-bromo-2-methylpropionyl]-β-cyclodextrin). *Tetrahedron Lett.* **46**, 2227-2229 (2005).

5 Whiteley, J. M., Woo, J. H., Hu, E., Nam, K.-W. & Lee, S.-H. Empowering the lithium metal battery through a silicon-based superionic conductor. *J. Electrochem. Soc.* **161**, A1812-A1817 (2014).

6 Zhang, Z. *et al.* Interface re-engineering of $Li_{10}GeP_2S_{12}$ electrolyte and lithium anode for all-solid-state lithium batteries with ultralong cycle life. *ACS Appl. Mater. Interfaces* **10**, 2556-2565 (2018).

7 Woo, J. H. *et al.* Nanoscale interface modification of $LiCoO_2$ by Al_2O_3 atomic layer deposition for solid-state Li batteries. *J. Electrochem. Soc.* **159**, A1120-A1124 (2012).

8 Xie, D. *et al.* High ion conductive Sb_2O_5-doped β-Li_3PS_4 with excellent stability against Li for all-solid-state lithium batteries. *J. Power Sources* **389**, 140-147 (2018).

9 Ulissi, U., Agostini, M., Ito, S., Aihara, Y. & Hassoun, J. All solid-state battery using layered oxide cathode, lithium-carbon composite anode and thio-LISICON electrolyte. *Solid State Ionics* **296**, 13-17 (2016).

10 Choi, S. J. *et al.* LiI-doped sulfide solid electrolyte: enabling a high-capacity slurry-cast electrode by low-temperature post-sintering for practical all-solid-state lithium batteries. *ACS Appl. Mater. Interfaces* **10**, 31404-31412 (2018).

11 Finsterbusch, M. *et al.* High capacity garnet-based all-solid-state lithium batteries: fabrication and 3D-microstructure resolved modeling. *ACS Appl. Mater. Interfaces* **10**, 22329-22339 (2018).

12 Chen, R. J. *et al.* Addressing the interface Issues in all-solid-state bulk-type lithium ion battery via an all-composite approach. *ACS Appl. Mater. Interfaces* **9**, 9654-9661 (2017).

13 Park, M.-S., Jung, Y.-C. & Kim, D.-W. Hybrid solid electrolytes composed of poly(1,4-butylene adipate) and lithium aluminum germanium phosphate for all-solid-state $Li/LiNi_{0.6}Co_{0.2}Mn_{0.2}O_2$ cells. *Solid State Ionics* **315**, 65-70 (2018).

14 Wakayama, H., Yonekura, H. & Kawai, Y. Three-dimensional bicontinuous nanocomposite from a self-assembled block copolymer for a high-capacity all-solid-state lithium battery cathode. *Chem. Mater.* **28**, 4453-4459 (2016).

15 Ates, T., Keller, M., Kulisch, J., Adermann, T. & Passerini, S. Development of an all-solid-state lithium battery by slurry-coating procedures using a sulfidic electrolyte. *Energy Storage Mater.* **17**, 204-210 (2019).

16 Hovington, P. *et al.* New lithium metal polymer solid state battery for an ultrahigh energy: nano C-$LiFePO_4$ versus nano $Li_{1.2}V_3O_8$. *Nano Lett.* **15**, 2671-2678 (2015).

17 Porcarelli, L. *et al.* Single-ion triblock copolymer electrolytes based on poly(ethylene oxide)

and methacrylic sulfonamide blocks for lithium metal batteries. *J. Power Sources* **364**, 191-199 (2017).

18 Chen, H. *et al.* Stable seamless interfaces and rapid ionic conductivity of Ca-CeO_2/LiTFSI/PEO composite electrolyte for high-rate and high-voltage all-solid-state battery. *Adv. Energy Mater.* **10**, 2000049 (2020).

19 Ma, M. *et al.* Designing weakly solvating solid main-chain fluoropolymer electrolytes: synergistically enhancing stability toward Li anodes and high-voltage cathodes. *ACS Energy Lett.* **6**, 4255-4264 (2021).

20 Huo, H. *et al.* Anion-immobilized polymer electrolyte achieved by cationic metal-organic framework filler for dendrite-free solid-state batteries. *Energy Storage Mater.* **18**, 59-67 (2019).

21 Fan, L. Effect of modified SiO_2 on the properties of PEO-based polymer electrolytes. *Solid State Ionics* **164**, 81-86 (2003).

22 Lin, D. *et al.* High ionic conductivity of composite solid polymer electrolyte via in situ synthesis of monodispersed SiO_2 nanospheres in poly(ethylene oxide). *Nano Lett.* **16**, 459-465 (2016).

23 Choi, J.-H., Lee, C.-H., Yu, J.-H., Doh, C.-H. & Lee, S.-M. Enhancement of ionic conductivity of composite membranes for all-solid-state lithium rechargeable batteries incorporating tetragonal $Li_7La_3Zr_2O_{12}$ into a polyethylene oxide matrix. *J. Power Sources* **274**, 458-463 (2015).

24 Sun, C. *et al.* Fast lithium ion transport in solid polymer electrolytes from polysulfide-bridged copolymers. *Nano Energy* **75**, 104976 (2020).

25 Atik, J. *et al.* Cation-assisted lithium-ion transport for high-performance PEO-based ternary solid polymer electrolytes. *Angew. Chem., Int. Ed. Engl.* **60**, 11919-11927 (2021).

26 Martinez-Ibañez, M. *et al.* Unprecedented improvement of single Li-ion conductive solid polymer electrolyte through salt additive. *Adv. Funct. Mater.* **30**, 2000455 (2020).

27 Homann, G., Stolz, L., Neuhaus, K., Winter, M. & Kasnatscheew, J. Effective optimization of high voltage solid-state lithium batteries by using poly(ethylene oxide)-based polymer electrolyte with semi-interpenetrating network. *Adv. Funct. Mater.* **30**, 2006289 (2020).

28 Mackanic, D. G. *et al.* Decoupling of mechanical properties and ionic conductivity in supramolecular lithium ion conductors. *Nat. Commun.* **10**, 5384 (2019).

29 Yao, W., Li, Y. & Huang, X. Fluorinated poly(meth)acrylate: synthesis and properties. *Polymer* **55**, 6197-6211 (2014).

30 Liu, J. *et al.* Nonflammable and high-voltage-tolerated polymer electrolyte achieving high stability and safety in 4.9 V-class lithium metal battery. *ACS Appl. Mater. Interfaces* **11**, 45048-45056 (2019).

31 Xu, H., Xie, J., Liu, Z., Wang, J. & Deng, Y. Carbonyl-coordinating polymers for high-voltage solid-state lithium batteries: solid polymer electrolytes. *MRS Energy Sustain.* **7**, 1-25 (2020).

32 Matyjaszewski., K. & Xia., J. Atom transfer radical polymerization. *Chem. Rev.* **101**, 2921-2990 (2001).

33 Ren, J. M. *et al.* Star polymers. *Chem. Rev.* **116**, 6743-6836 (2016).

34 N.H. Aloorkar, A.S. Kulkarni, Patil, R. A. & Ingale, D. J. Star polymers: an overview. *Inter. J. Pharm. Sci. Nanotechnol.* **5**, 1675-1684 (2012).

35 Davis, M. E. & Brewster, M. E. Cyclodextrin-based pharmaceutics: past, present and future. *Nat. Rev. Drug Discovery* **3**, 1023-1035 (2004).
36 Sun, B. *et al.* Ion transport in polycarbonate based solid polymer electrolytes: experimental and computational investigations. *Phys. Chem. Chem. Phys.* **18**, 9504-9513 (2016).
37 Wu, I. D. & Chang, F.-C. Determination of the interaction within polyester-based solid polymer electrolyte using FTIR spectroscopy. *Polymer* **48**, 989-996 (2007).
38 Tominaga, Y. Ion-conductive polymer electrolytes based on poly(ethylene carbonate) and its derivatives. *Polym. J.* **49**, 291-299 (2016).
39 Chazalviel, J. Electrochemical aspects of the generation of ramified metallic electrodeposits. *Phys. Rev. A* **42**, 7355-7367 (1990).
40 Jana, A., Woo, S. I., Vikrant, K. S. N. & García, R. E. Electrochemomechanics of lithium dendrite growth. *Energ. Environ. Sci.* **12**, 3595-3607 (2019).
41 Wang, Y. *et al.* An in situ formed surface coating layer enabling $LiCoO_2$ with stable 4.6 V high-voltage cycle performances. *Adv. Energy Mater.* **10**, 2001413 (2020).
42 Wang, Y. *et al.* Solid-state rigid-rod polymer composite electrolytes with nanocrystalline lithium ion pathways. *Nat. Mater.* **20**, 1255-1263 (2021).
43 Liu, W. *et al.* Enhancing ionic conductivity in composite polymer electrolytes with well-aligned ceramic nanowires. *Nat. Energy* **2**, 17035 (2017).
